# NLRP3 Deficiency Protects Against Intermittent Hypoxia-Induced Neuroinflammation and Mitochondrial ROS by Promoting the PINK1-Parkin Pathway of Mitophagy in a Murine Model of Sleep Apnea

**DOI:** 10.3389/fimmu.2021.628168

**Published:** 2021-02-24

**Authors:** Xu Wu, Linjing Gong, Liang Xie, Wenyu Gu, Xinyuan Wang, Zilong Liu, Shanqun Li

**Affiliations:** ^1^ Department of Pulmonary Medicine, Zhongshan Hospital, Fudan University, Shanghai, China; ^2^ Clinical Centre for Sleep Breathing Disorder and Snoring, Zhongshan Hospital, Fudan University, Shanghai, China; ^3^ Department of Urology, Shanghai Tenth People’s Hospital, Tongji University School of Medicine, Shanghai, China; ^4^ Department of Orthopaedics, Zhongshan Hospital, Fudan University, Shanghai, China

**Keywords:** obstructive sleep apnea (OSA), neuroinflammation, nucleotide‐binding domain like receptor protein 3 (NLRP3), reactive oxygen species (ROS), parkin-mediated mitophagy

## Abstract

Obstructive sleep apnea (OSA) associated neurocognitive impairment is mainly caused by chronic intermittent hypoxia (CIH)-triggered neuroinflammation and oxidative stress. Previous study has demonstrated that mitochondrial reactive oxygen species (mtROS) was pivotal for hypoxia-related tissue injury. As a cytosolic multiprotein complex that participates in various inflammatory and neurodegenerative diseases, NLRP3 inflammasome could be activated by mtROS and thereby affected by the mitochondria-selective autophagy. However, the role of NLRP3 and possible mitophagy mechanism in CIH-elicited neuroinflammation remain to be elucidated. Compared with wild‐type mice, NLRP3 deficiency protected them from CIH-induced neuronal damage, as indicated by the restoration of fear-conditioning test results and amelioration of neuron apoptosis. In addition, NLRP3 knockout mice displayed the mitigated microglia activation that elicited by CIH, concomitantly with elimination of damaged mitochondria and reduction of oxidative stress levels (malondialdehyde and superoxide dismutase). Elevated LC3 and beclin1 expressions were remarkably observed in CIH group. *In vitro* experiments, intermittent hypoxia (IH) significantly facilitated mitophagy induction and NLRP3 inflammasome activation in microglial (BV2) cells. Moreover, IH enhanced the accumulation of damaged mitochondria, increased mitochondrial depolarization and augmented mtROS release. Consistently, NLRP3 deletion elicited a protective phenotype against IH through enhancement of Parkin-mediated mitophagy. Furthermore, Parkin deletion or pretreated with 3MA (autophagy inhibitor) exacerbated these detrimental actions of IH, which was accompanied with NLRP3 inflammasome activation. These results revealed NLRP3 deficiency acted as a protective promotor through enhancing Parkin-depended mitophagy in CIH-induced neuroinflammation. Thus, NLRP3 gene knockout or pharmacological blockage could be as a potential therapeutic strategy for OSA-associated neurocognitive impairment.

## Introduction

Obstructive sleep apnea (OSA) is characterized by the repetitive narrowed or collapsible upper airway, resulting in recurrent hypoxia during sleep (1). As the foremost pathophysiological process of OSA, nocturnal chronic intermittent hypoxia (CIH), causes structural neuron damage and dysfunction in the CNS that are most likely hippocampal-dependent and persistent (2). Clinically, it manifests as neurocognitive and behavioral deficits, or memory and learning impairments (3, 4). Emerging evidence showed that the deleterious effect eliciting by hypoxia in cognitive impairments may be related to ion-channel alterations, glutamate excitotoxicity release (5), oxidative stress overactivation (6), and upregulation of proinflammatory mediators (7). However, the precise mechanisms of neuroinflammation and oxidative stress in cognitive impairment induced by CIH exposure from OSA needs to be further explored.

Sustained hypoxia leads to the activation of microglia, thereby inducing a robust source of oxidative stress mainly through damaged mitochondria, NADPH oxidase and nitric oxide (NO) overproduction (6, 8). In response to cellular danger signals, the nucleotide‐binding domain like receptor protein 3 (NLRP3) is recognized as a multiprotein complex sensor to interact with ASC (adapter apoptosis-associated speck-like protein containing a caspase recruitment domain) and procaspase-1, and then form the NLRP3 inflammasome, ultimately leading to the cleavage of caspase-1 and the release of pro-inflammatory interleukin (IL)-1β (9). Moreover, NLRP3 inflammasome has garnered much attention in a variety of neuroinflammatory and neurodegenerative diseases (10, 11). Given that microglia are dominant pro-inflammatory cells in CNS, caspase-1-processed cytokines IL-1β could be released by microglia in pathological conditions, thus aggravating the progression of neuroinflammation (11). And, inhibiting NLRP3 inflammasome activation attenuates neuroinflammation and improves neurological function in brain injury (12). In this regard, the inflammasome-mediated microglia activation may play an important role in the neuroinflammatory conditions.

Previously, we suggested that mitochondrial (mt) damage was a potential cause of NLRP3 inflammasome activation (13). Shimada et al. demonstrated that NLRP3 inflammation can be activated through mt damage-induced apoptotic cascade (14). Moreover, it has also been proven that the combined roles for caspase-8 and caspase-1/NLRP3 causing IL-1β maturation (15), indicating the crosstalk between apoptosis and pyroptosis (16). The mitochondria-selective autophagy, termed as mitophagy, is a conserved self-degradation process that can be negatively regulated by NLRP3 inflammasome (17, 18). To initiate mitophagy, the ubiquitin kinase PTEN-induced putative kinase1 (PINK1) is recruited to the mitochondrial outer membrane to further induce the ubiquitin phosphorylation (19). Subsequently, PINK1 recruits the E3 ubiquitin ligase Parkin from cytosol to damaged mitochondria to establish ubiquitin chains and assemble autophagy receptors (20). The process results in the commencement of mitophagy and then elimination of mitochondrial ROS that is required for NLRP3 inflammasomes induction. Most studies on Parkin-dependent mitophagy have been relevant to neurologic diseases (e.g., Parkinson’s disease) (21, 22). However, the role of mitophagy in the setting of OSA is not clear yet.

In this study, we elucidated whether the mitophagy was linked to the protective effect of NLRP3 deficiency from CIH-induced neuroinflammation. Specifically, we focused our *in vitro* study on the Parkin-dependent mitophagy under CIH and determined the NLRP3-mediated mechanism controlling mitophagy. The present study reveals the relevance of Parkin-mediated mitophagy as the protective mechanism against neuroinflammation and presents NLRP3 as a potential therapeutic target.

## Material and Methods

### Animal and Experimental Model of CIH

NLRP3^−/−^ mice and aged-matched controls on C57BL/6 background (Jackson Laboratory, Sacramento, CA) were housed under standard conditions with a 12‐hr light/12‐hr dark cycle at 22–24°C and allowed free access to water and food. The project was approved by the Medical Experimental Animal Administrative Committee of the Shanghai Medical College of the Fudan University, in accordance with the guidelines implemented by the National Institutes of Health Guide regarding the care and use of animals for experimental procedures. All effects were made to minimize animal suffering. WT or NLRP3^−/−^ mice (male, 6–7 weeks old, 20–22 g) were randomly divided into four groups of six: the normal air (NA) plus WT mice group, the NA plus NLRP3-/- mice group, the CIH plus WT mice group, and the CIH plus NLRP3-/- mice group. The mice exposed to CIH were placed inside custom‐made (28.5cm × 30.0cm × 51.5cm) chambers where flows of oxygen and nitrogen were controlled to obtain the desired profile of changes in oxygen level. CIH was administered for 10 h/day, from 7:00AM to 5:00PM, with the oxygen level oscillating between 24% and 7% with a period of 60s. The NA or CIH treatment was lasted for 7 d/week for 5 weeks. The oxygen concentration was measured automatically using an oxygen analyzer (Corporation, Shanghai, China).

### Cell Culture and Treatment

Murine BV-2 microglial cell line was obtained from the Chinese Academyof Medical Sciences (Beijing, China). BV-2 cells were maintained in DMEM medium supplemented with 10% fetal bovine serum, 100 U/ml penicillin and 100 mg/ml streptomycin (Sigma) in a humidified atmosphere incubator until 70–80% confluent. Microglial cells were maintained in a 37°C custom-made chamber with 5% CO_2_. Consistent with animal experiments, the O_2_ concentration of this chamber was alternated between 0 and 22% every 30 min *via* injecting oxygen or nitrogen. The dissolved O_2_ inside the culture medium was monitored by a laser O_2_ probe (Biospherix) and the IH reached to 5% O_2_ and 21% O_2_ as hypoxic and normoxic values sensed by the cells. After exposing to IH for 3, 6, 12, or 24 h, the microglial cells were collected for immunoblotting, flow cytometry analysis, or immunofluorescent staining. We constructed the NLRP3 knockout (KO) cell line *via* lentivirus transfection (LV) strategy (MOI 20). Cells were plated in 24-well (1 × 10^4^) and cultured overnight before transfection. The cells were transfected with LV-NLRP3 or LV-NC, according to the manufacture’s protocols (Obio Technology, Shanghai, China). After 72-h transfection, cells were selected with Puromycin (5 ug/ml) for 10 days to obtain stable strains. Parkin short hairpin RNA (shRNA) plasmids synthesized by GenePharma (Shanghai, China) were diluted in Opti-MEM^®^ medium (Thermo Scientific). Transfection with shRNA was done by Lipofectamine 2000 (Invitrogen) according to the manufacturer’s instructions. After 6 h of transfection, cells were cultured in 10%FBS DMEM medium for 48 h. The sequence of shRNA for Parkin was as follows: 5’-GCTTTGAACCTGATCACCAGC-3’. The BV2 cells incubated with 3-MA (5 mM) or the PBS vehicle for 6 h before exposure to IH.

### Contextual Fear Conditioning Test

Based on a previously published model, contextual fear conditioning test (FCT) includes two parts: a training phase at 1 day before surgical operation and a test phase on postoperative 1 and 3 days. In training phase, mice receive fear conditioning to establish the long‐term memory. Each animal was allowed to adapt to the conditioning chamber (context) for 120 s, followed by six cycles of conditional‐unconditional stimuli. A cycle of conditional or unconditional stimuli was then applied as a 20 s, 80 dB tone (conditional stimuli)-30 s delay 5 s, 0.75 mA electrical foot shock (unconditional stimuli). The cycles of conditional/unconditional stimuli were separated by random intervals from 45 to 60 s. The context test, which represents hippocampal‐dependent memory, is the major part of the test phase of the FCT. At post-operative 1 and 3 days, all mice were returned into the original conditioning chamber for 5 min, where no tone and no shock were released. The percentage of freezing time (not moving) was captured and collected by Any‐Maze software (Xinruan, Shanghai, China).

### Immunohistochemical Analysis

For histological analysis, mice were anesthetized and perfused transcardially with cold phosphate buffer solution (PBS). Then the fresh brain was fixed with 4% paraformaldehyde (PFA) and then were embedded in paraffin, and cut into 4-μm-thick sections that were deparaffinized with xylene and rehydrated in a graded series of alcohol. Antigen retrieval was carried out by microwaving in citric acid buffer. Sections were incubated with an antibody against ASC (1:100; Cell Signaling Technology, Danvers, MA, USA), washed, and then incubated with secondary antibody for 1 h at room temperature.

### Immunofluorescence Analysis

Terminal deoxynucleotidyl transferase dUTP nick-end labeling (TUNEL) assay was to detect apoptotic cells with *in situ* cell death detection kit (Roche, Netley, NJ) according to the manufacturer’s protocol. The final average percentage of apoptotic cells was calculated as TUNEL+/DAPI+ cells in six sections (observed at ×200 magnification). Immunofluorescent staining was also performed on brain slice. Sections were incubated overnight at 4°C with antibodies against LC3 (1:200, Abcam, Cambridge, UK), and Ionized Calcium Binding Adapter Molecule1 (Iba1) (1:100; Wako, Japan). After washing, the sections were incubated with secondary antibodies for 1 h at room temperature. Cell nuclei were counter-stained with 4’,6-diamidino-2-phenylindole (DAPI).

BV2 cells were fixed with 4% paraformaldehyde at room temperature for 15–20 min and washed in PBS for three washes of 10 min each. The BV-2 cells were permeabilized for 10 min with 0.1% Triton X-100 in PBS, washed with PBS, and blocked in 1% bull serum albumin (BSA) for 30 min. The coverslips were incubated with mouse-anti-Parkin (1:100; Santacruz Biotechnologies), rabbit-anti-TOM20 (1:200; Beyotime Biotechnology), mouse-anti-LC3 (1:500; Cell Signal Technology), or rabbit-anti-LC3 (1:500; Cell Signal Technology) overnight at 4°C. After washing, the secondary antibody was added. The samples were incubated for 1 h at room temperature, and ultimately examined under a microscope (Olympus IX73, Japan) or a confocal microscope (Fluoview 1000, Olympus, Tokyo, Japan).

### Determination of Oxidative Stress Production

The hippocampal and cortex tissues were homogenized in lysis buffer and centrifuged at 10,000 × g for 10 min at 4°C. The supernatants were collected to assess the malondialdehyde (MDA) content and activities of superoxide dismutase (SOD) (Beyotime, China). All results were normalized to the protein concentration and expressed as U/mg protein or nmol/mg protein as appropriate.

### Mitochondrial ROS Measurement

The intact mitochondria of hippocampal region were isolated from brain using a commercial kit (Beyotime, China). The following experimental procedures were conducted according to the manufacturer’s instructions. Briefly, the homogenate was centrifuged at 6,000 g at 4°C for 5 min. The collected supernatant was further centrifuged at 11,000 g at 4°C for 10 min to obtain a mitochondrial pellet. Then, the mitochondrial ROS was detected utilizing the ROS assay kit (Genmed Scientifics, Shanghai, China).

Cellular mitochondrial ROS activity was assessed with Mito SOX Red (Invitrogen) staining. BV2 cells were seeded onto six-well blank plates with a density of 1 × 10^5^/ml with 3 parallel wells in each group. Cells were incubated with MitoSOX Red probe at a final concentration of 5 μM for 10 min at 37°C and washed twice with PBS. Quantification of mtROS release was conducted by FACSCalibur (BD Biosciences). All data were analyzed on FlowJo software (Tree Star, San Carlos, CA).

### Mitochondrial Membrane Potential

According to the manufacturer’s instructions, changes in mitochondrial membrane potential (MMP) were determined using a JC‐1 mitochondrial membrane potential assay kit (C2006, Beyotime, China). BV2 cells (1 × 10^5^) were incubated with JC-1 (10 μg/ml) staining buffer for 20 min at 37°C. Then the cells were washed with PBS and observed under microscope or analyzed by FlowJo software. The ratio of aggregates (red fluorescence; good mitochondrial membrane potential) to monomers (green fluorescence; loss of mitochondrial membrane potential) was regarded as a marker of MMP loss.

### Cell Apoptosis Detected by Flow Cytometry

As we previously described, BV-2 cells (1 × 10^5^) were collected and then incubated with Annexin V-fluorescein isothiocyanate (FITC) and propidium iodide (PI) (Annexin V Apoptosis Detection Kit; BD Biosciences). The lower and upper right quadrants show the proportions of the early (Annexin V+/PI−) and the late (Annexin V+/PI+) apoptotic cells, respectively.

### Western Blotting

Proteins from hippocampal tissues or cell lysates (20–40 μg of total protein) were separated by sodium dodecyl sulfate-polyacrylamide gel electrophoresis and transferred to a polyvinylidene difluoride membrane, and blocked with 5% BSA/Tris-buffered saline with Tween-20 (TBST) for 1 h at room temperature. Then, the membranes were incubated overnight at 4°C with primary antibodies at the following dilutions: NLRP3 (1:1000; abcam), caspase-1 (1:200; Santa cruz), ASC (1:1000; abcam), Bax (1:1000; CST), Bcl-2 (1:2000; CST), caspase-3 (1:1000; CST), Parkin (1:1000; CST), PINK1 (1:1000; abcam), LC3 (1:1000; CST), ATG5 (1:1000; CST), ATG7 (1:1000; CST), P62 (1:1000; CST), Beclin-1 (1:1000; CST), TOM20 (1:500; Beyotime), and GAPDH (1:500; Beyotime), followed by incubation with appropriate secondary antibodies after thoroughly washing three times with TBST. The bands were visualized using the chemiluminescence (ECL) detection system (Thermo Fisher Scientific) and quantified by Image J gel analysis software. Expression levels were normalized against GAPDH.

### Quantitative Reverse Transcriptase-PCR (qRT-PCR)

Total RNA was extracted from hippocampal tissues of WT and NLRP3^-/-^ mice from different groups using TRIzol reagent (Invitrogen, Carlsbad, CA) to detected relative IL-1β mRNA level. qRT-PCR was performed on a real-time PCR system (Applied Biosystems 7500HT; Applied Biosystems, Foster City, CA) using SYBR-Green Master Mix Plus (Toyobo, Osaka, Japan) according to the manufacturer’s instructions. The expression of IL-1β mRNA was normalized to the mRNA level of GAPDH. The primers specific to IL-1β mRNA used were purchased from Sangon Biotech (Shanghai, China). And, the sequences were as followed: IL-1β-Forward: 5’-GGGCCTCAAAGGAAAGAATC-3’, IL-1β-Reverse: 5’-TACCAGTTGGGGAACTCTGC-3’.

### Transmission Electron Microscopy (TEM)

BV2 cells were fixed with 2% glutaraldehyde and 1% osmium tetroxide in 0.1 M phosphate buffer (PB) (pH 7.4) at 4°C. Then the cells were sliced into 70~80-nm-thick sections. After being dehydrated in ethanol with 3% uranyl acetate, embedded, and stained with lead citrate for contrast, the sections were examined under transmission electron microscope (JEM 1011, Japan). A blinded pathologist was invited to quantify each section independently.

### Statistical Analysis

Data were analyzed with GraphPad Prism-7 statistic software (La Jolla, CA). All values were expressed as mean ± standard error of the mean (SEM). Qualitative data were analyzed by two-tailed t-test between two groups or one-way ANOVA, followed by post-hoc multiple comparisons among multiple groups. P < 0.05 was considered statistically significant. At least three independent experiments were performed in duplicate with all the results.

## Results

### NLRP3 Deficiency Alleviates CIH‐Induced Cognitive Dysfunction and Neuronal Apoptosis of Hippocampus

**Figure 1:**
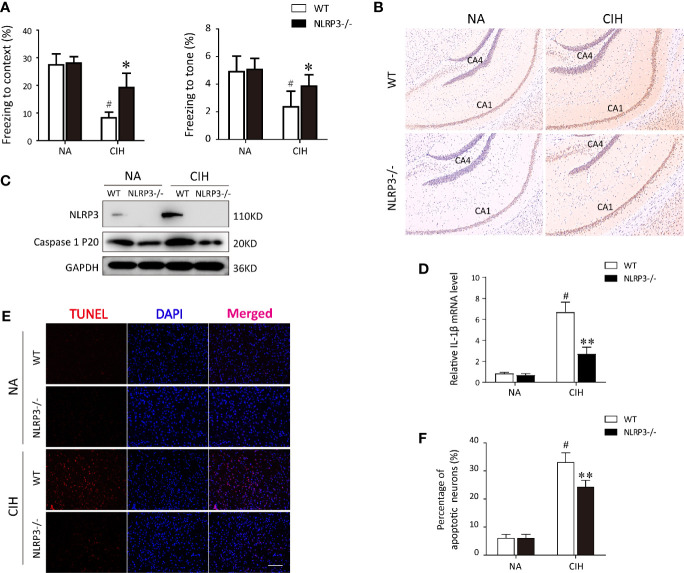
Protective effect of NLRP3 deficiency on CIH-induced neuronal damage *in vivo*. **(A)** Effects of NLRP3 deficiency on fear-conditioning tests results. ^#^P < 0.01 versus NA group; *P < 0.05 versus CIH + WT group (n = 6 mice/group). **(B)** Representative photographs of immunostaining for ASC in hippocampus (×200 magnification). **(C)** Protein expressions of NLRP3 and cleaved caspase‐1 in hippocampus tissues of NLRP3^−/−^ mice and WT mice measured by western blot. **(D)** Expression levels of IL-1β mRNA in the hippocampi from WT or NLRP3^-/-^ mice, exposing to NA or CIH. ^#^P < 0.01 versus NA group; **P < 0.01 versus CIH + WT group. **(E, F)** The neuronal apoptosis was assayed with TUNEL. Typical immunofluorescent micrographs for TUNEL (red) staining from hippocampus tissues of each group. Scale bar = 100 μm. Quantitative analyses of the number of TUNEL-positive cells. ^#^P < 0.01 versus NA group; **P < 0.01 versus CIH + WT group. All data are presented as means ± SEM. CIH, chronic intermittent hypoxia; NA, normal air; WT, wild type.

To investigate the potential mechanism regarding CIH-induced neurocognitive impairment, we first established a CIH mice model as described before (23). When the mice were performed the fear-conditioning test, we found the freezing times in the contextual and tone conditional tasks were significantly lower in the CIH group compared with the NA control group (P < 0.01, **Figure 1A**). Dramatically, NLRP3 deficiency tended to restore the decreased freezing time as compared to WT mice after CIH exposure (P < 0.05, **Figure 1A**). As illustrated in **Figure 1B**, ASC was highly expressed in hippocampus of WT mice exposed to CIH. In contrast, the immunohistochemical staining showed the ASC was faintly stained in the hippocampus of NLRP3^−/−^ mice upon CIH treatment. Western blotting was applied to the cortex and hippocampus samples to detect NLRP3 and cleaved caspase‐1. The levels of NLRP3 and activated caspase‐1 in WT mice were shown to be upregulated in response to CIH stimulation, but lowly detectable in NLRP3^−/−^ mice (**Figure 1C** and **Figure S1**). Besides, the expression level of IL-1β mRNA in the hippocampi of WT mice increased obviously after CIH treatment, which can be alleviated by the *NLRP3* gene knockout (**Figure 1D**). Neuronal apoptosis caused by neuroinflammation is one important mechanism of cognitive impairment induced by CIH (11). To ascertain it, the CIH group showed a significant increase in the number of TUNEL positive cells by immunofluorescence (33 ± 7.78%, P < 0.01). However, the absence of NLRP3 in mice underwent a 26% attenuation of the apoptotic cells compared with the WT mice following CIH exposure (P < 0.01, **Figures 1E, F**). It is worthy to note the enhanced neuronal apoptosis subjected to CIH in the hippocampal region, thus selectively influencing contextual fear conditioning and hippocampal-dependent memory consolidation. Taken together, these results suggested the involvement of the NLRP3 inflammasome in the pathogenesis of CIH‐induced cognitive dysfunction and neuronal damage.

### NLRP3 Deficiency Attenuates CIH‐Induced Microglia Activation and Oxidative Stress in Hippocampus

**Figure 2:**
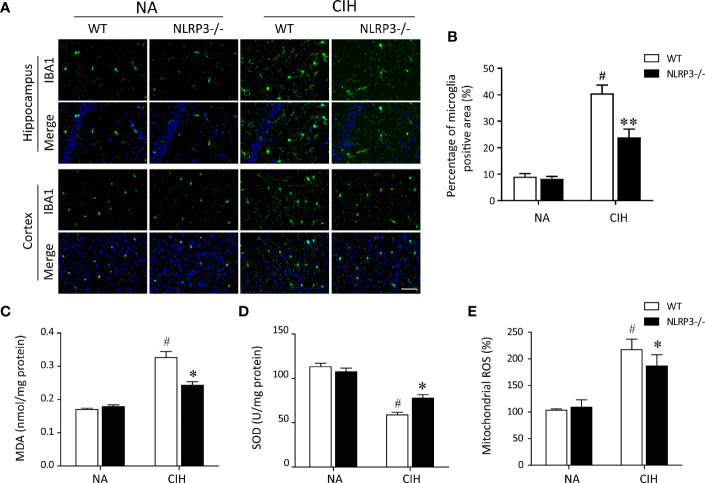
NLRP3 deficiency prevents CIH-induced microglia activation and oxidative stress in hippocampus. **(A)** Microglia were detected with ionized calcium binding adapter molecule 1 (Iba1) antibody. Photomicrographs showed the Iba‐1 (green) immunofluorescent staining from hippocampus and cortex tissues of each group. Note that Iba‐1 was highly expressed in response to CIH. Scale bar = 50 μm. **(B)** Bar graphs displayed the percentage of Iba-1 positive cells per high-power field (n = 6). The hippocampal MDA content **(C)**, SOD activities **(D)**, and mitochondrial ROS levels **(E)** were measured in tissue homogenates (n = 6 per group). The data are presented as means ± SEM. ^#^P < 0.01 versus NA group; *P < 0.05 and **P < 0.01 versus CIH + WT group. CIH, chronic intermittent hypoxia; NA, normal air; WT, wild type; MDA, malondialdehyde; SOD, superoxide dismutase.

Since microglial cells were regarded as a predominant contributor to the pathogenesis of CIH-induced neuroinflammation (7, 24), we wonder whether NLRP3 inflammasome influences the behavioral changes of microglia during CIH exposure. As displayed in **Figure 2A**, CIH remarkably increased the numbers of activated microglia both in cortex and hippocampus compared with NA control group. However, in NLRP3^−/−^ mice, the activated microglia were seldom observed in cortex and hippocampus section upon CIH treatment (**Figure 2B**). It is important to emphasize that the inflammasome activation to be associated with defective mitochondrial function and ROS accumulation (14). Hence, to examine the occurrence of mitochondrial dysfunction in microglia after CIH, the level of mitochondrial ROS (mtROS) production was measured. We found that exposed WT mice to CIH were shown to have the increased MDA levels and decreased SOD activities (**Figures 2C, D**), both of which were characterized as biomarkers of oxidative stress injury. Moreover, the hypoxic oxidative stress was accompanied with increased mtROS, as shown in **Figure 2E**. However, compared with the WT mice exposed to CIH, NLRP3 knockout eradicated the changes in MDA content and ameliorated the mitochondrial damage in hippocampus and cortex. According to the above results, our data demonstrated that activated microglial cells in a CIH model caused neuroinflammation and subsequently affected the behavior of the mice through producing a mass of mtROS.

### NLRP3 Deficiency Enhances CIH-Induced Mitophagy and Increases Parkin Expression in Hippocampus

**Figure 3:**
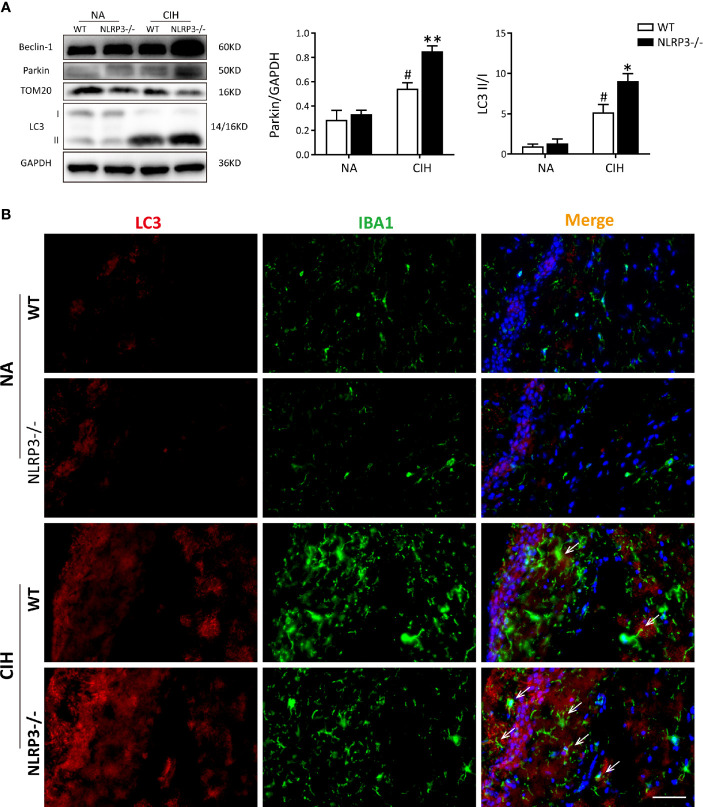
NLRP3 deficiency enhances CIH-induced mitophagy and increases parkin expression *in vivo*. **(A)** Protein expressions of Beclin-1, Parkin, TOM20, and LC3 in hippocampal tissues of NLRP3^−/−^ mice and WT mice measured by western blot. Values are expressed as means ± SEM. ^#^P < 0.05 versus NA group; *P < 0.05 and **P < 0.01 versus CIH + WT group. **(B)** Representative images of double-labeled with LC3 and IBA1 (microglia, white arrow) in hippocampus revealed the increased autophagosome formation after 5 weeks of CIH exposure, especially the NLRP3-/- group. Scale bars = 50 μm. CIH, chronic intermittent hypoxia; NA, normal air; WT, wild type.

Next, the level of mitophagy was further detected in hippocampal tissues *via* immunoblotting and immunofluorescence staining. Western blot analysis showed obvious mitophagy induction in mice exposed to CIH, especially the NLRP3^−/−^ mice, evidencing by decrease in TOM20 protein levels, and increase in LC3 II and Beclin-1 protein levels. In addition, CIH significantly increased the protein expression of Parkin in hippocampus compared with NA group, and NLRP3 knockout reinforced this trend (**Figure 3A**). To examine the formation of autophagosome in microglia of hippocampus, confocal microscopy showed that the expressions of autophagy markers LC3 were robustly stained in CIH group compared to NA control group. Further co-staining with IBA1 revealed that NLRP3 knockout remarkably enhanced the co-location of LC3 and IBA1 both in NA group and CIH group (**Figure 3B**). In this regard, mitophagy and autophagy could be up-regulated by CIH. NLRP3 deletion may further increase the formation of mitophagosome and autophagosome, which was also observed in microglia of hippocampus. Nevertheless, whether and how mtROS, NLRP3 inflammasome, and mitophagy interacted with each other in microglial cells needs to be further explored.

### IH Facilitates NLRP3 Inflammasome Activation and Parkin-Mediated Mitophagy in BV2 Cells

**Figure 4:**
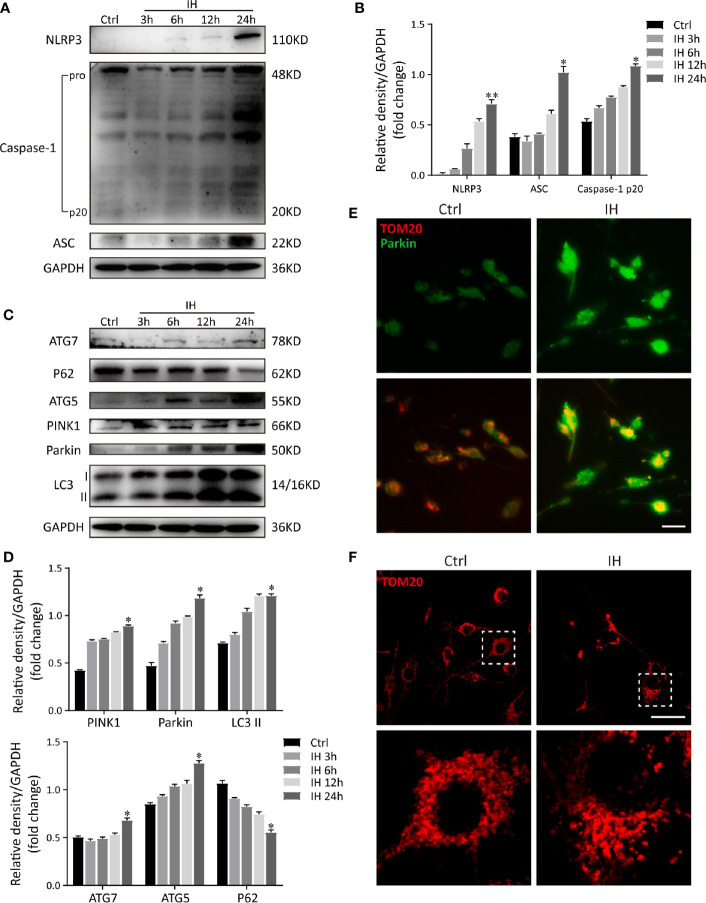
IH induces NLRP3 inflammasome activation and Parkin-mediated mitophagy in BV2 cells. **(A)** Protein levels of NLRP3, cleaved caspase-1 and ASC increased significantly in BV2 cells subjected to 24h of IH, indicating activation of NLRP3 inflammasome. **(B)** Densitometric quantification of relative protein expression normalized to GAPDH was shown on the bar graphs. **(C)** Western blot revealed the significant increased levels of autophagic and mitophagic markers (ATG-5, ATG-7, PINK1, Parkin, and LC3-II), and decreased p62 expression in response to IH. **(D)** Quantification of relative protein expression assessed by densitometric analysis with GAPDH as an internal control. (n = 3 in each group). Values are expressed as means ± SEM. *P < 0.05 versus control group; **P < 0.01 versus control group. **(E)** Cells were double-labeled with parkin (green) and mitochondrial outer membrane protein TOM20 (red). Immunofluorescence images showed more parkin-positive cells colocalized with TOM20 following IH, suggesting the mitophagy induction. bar = 50 μm. **(F)** Confocal imaging presented the mitochondrial network stained for the MitoTracker (TOM20), in NA and IH microglial cells. bar = 50 μm. Similar results were obtained from three independent experiments. IH, intermittent hypoxia.

To further study the regulatory relationship between mitophagy and NLRP3 inflammasome, we established an IH *in vitro* model as described previously (13, 23). As a selective form of specialized autophagy, mitophagy controls the turnover of dysfunctional, and damaged mitochondria, thus eliminating excessive mtROS as well as NLRP3-elicited inflammatory response (25, 26). Consistent with previous observations, NLRP3 inflammasome was activated in IH-induced microglial cells and all these results were in a time-dependent manner (**Figures 4A, B**). Meanwhile, immunoblot analysis showed that both autophagic and mitophagic activities (Atg7, p62, Atg5, LC3, Parkin, and PINK1) were augmented (**Figures 4C, D**). Considering that OSA is a kind of chronic disease, we finally chose IH exposure of 24 h as an *in vitro* model. Furthermore, as illustrated in **Figure 4E**, colocalization of Parkin (green) with mitochondrial related protein (TOM20, red) yielded that the bright green fluorescence of Parkin was largely enhanced following CIH exposure. Then, we further investigated the impact of IH on the changes to mitochondrial morphology in microglial cells by immunofluorescent staining. As illustrated in **Figure 4F**, exposure to IH can trigger the fragmentation of mitochondria in microglia cells, which was followed with the induction of mitophagy. In summary, these results suggested that the IH-related NLRP3 inflammasome activation was accompanied with Parkin-mediated mitophagy induction in microglia.

### NLRP3 Deficiency Exerts Protective Effect Against IH *via* Parkin-Mediated Mitophagy *In Vitro*

**Figure 5:**
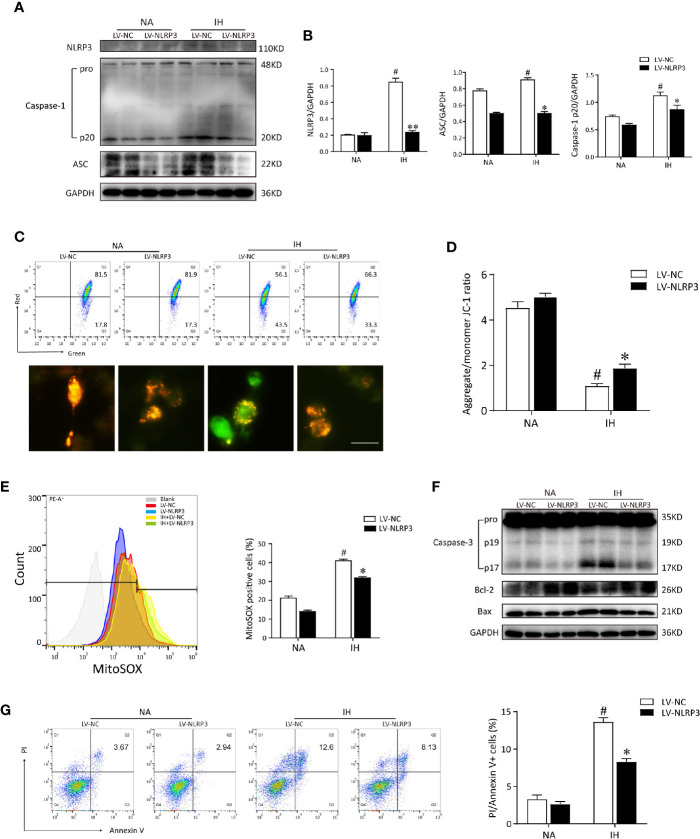
NLRP3 knockout restores the IH from the mitochondrial dysfunction and reduces mtROS production *in vitro*. **(A)** Immunoblot protein expressions of NLRP3, ASC and caspase-1 in IH-treated BV2 cells transfected with LV-NLRP3 or LV-NC. **(B)** The amounts of each protein were quantified by densitometry and expressed relative to the amount of GAPDH in the same samples. **(C)** LV-NC or LV-NLRP3 transfected BV2 cells were stained with JC-1 and analyzed by flow cytometry (upper) and microscopy (below). Scale bar = 50 μm. **(D)** Quantification of MMP was represented as the ratio red to green fluorescence. **(E)** LV-NC or LV-NLRP3 transfected cells were stained with MitoSOX and analyzed by flow cytometry. IH-elicited mtROS generation was inhibited by NLRP3 deficiency. **(F)** Cell lysates were immunoblotted for apoptotic proteins in BV2 cells. **(G)** Apoptosis of cells transfected with LV-NLRP3 in the presence or absence of IH was assayed by Annexin-V/PI staining. The quantitative rate of apoptosis was presented on the right histogram. Values are expressed as means ± SEM of three independent experiments. ^#^P < 0.05 versus LV-NC group; *P < 0.05 versus IH + LV-NC group; **P < 0.01 versus IH + LV-NC group. IH, intermittent hypoxia; NA, normal air; LV, lentivirus; mtROS, mitochondrial reactive oxygen species.

To distinguish the deleterious contribution of NLRP3 inflammasome *in vitro*, we constructed the NLRP3 knockout cell line *via* lentivirus transfection strategy, and then verified the NLRP3- elicited inflammatory response by western blot. As expected, the immunoblot images of NLRP3, cleaved caspase-1 and ASC were not obviously detectable in NLRP3-deleted cells, confirming the knockout efficiency. Moreover, accumulation of ASC and cleaved caspase 1 induced by IH was substantially abolished by deletion of NLRP3 (**Figures 5A, B**). It is generally considered that mitochondria dysfunction is tightly associated with MMP loss and mtROS, subsequently affects apoptosis (14). Afterward, we monitored the MMP levels, mtROS production, and examined cell apoptosis. In line with the *in vivo* results, our *in vitro* data demonstrated that IH alone disrupted the MMP (red to green ratio, 1.09 ± 0.19%) and augmented mtROS release. NLRP3 deletion also blocked the MMP loss upon stimulation with IH (red to green ratio, 1.87 ± 0.21%, **Figures 5C, D**). Consistently, IH‐triggered excessive mtROS generation (41.27 ± 2.27%) was significantly ameliorated by NLRP3 deficiency (32.13 ± 1.98%, **Figure 5E**). In addition, the expressions of cleaved caspase-3 p17 and pro-apoptotic protein Bax were both obviously upregulated in response to IH, whereas knockout of NLRP3 markedly attenuated their activations compared to the LV-NC group (**Figure 5F**). Notably, similar results were obtained in flow cytometric analysis, which showed the proportion of double-positive cells (Annexin V+/PI+) was the highest following IH exposure (13.39 ± 2.27%), while the percentage of apoptosis was significantly lower in NLRP3 knockout cells (8.65 ± 1.54%, p < 0.05, **Figure 5G**). These results suggested that deletion of NLRP3 can reduce CIH-triggered mitochondrial damage and alleviate microglial apoptosis.

**Figure 6:**
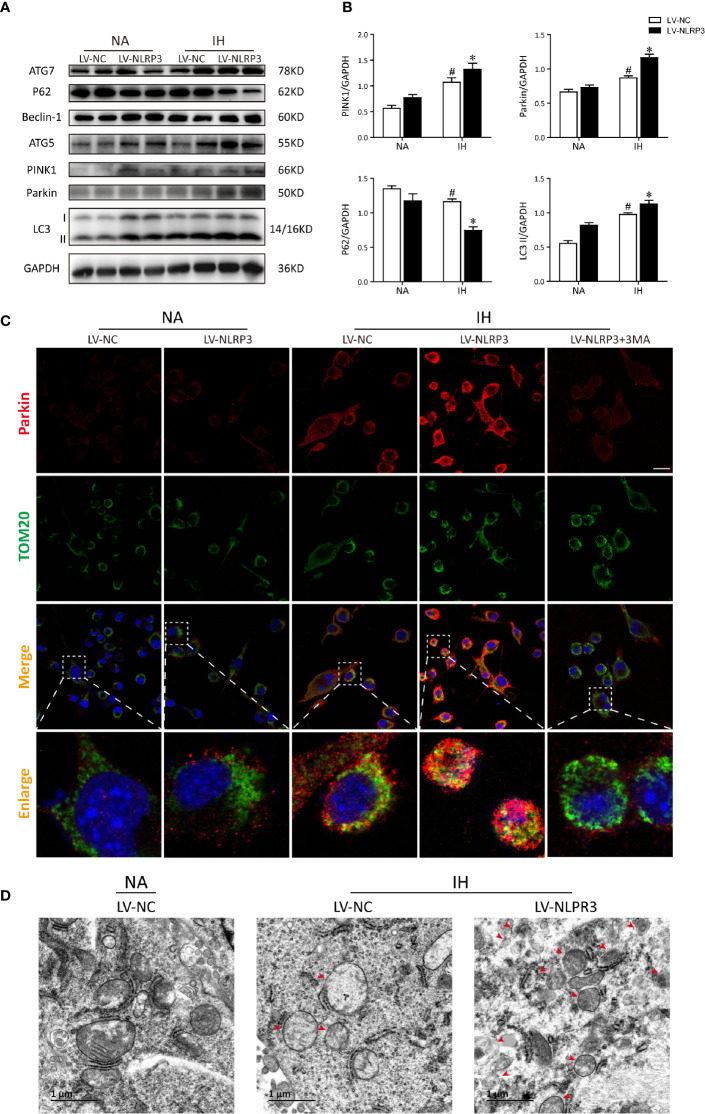
NLRP3 knockout provides protective effect against IH *via* Parkin-dependent mitophagy *in vitro*. **(A)** Representative western blot bands from BV2 cells transfected with LV-NLRP3 or LV-NC showed autophagic and mitophagic protein expression in the presence or absence of IH. **(B)** Densitometric quantification of PINK1, Parkin, P62, and LC3-II levels in comparison with GAPDH as a loading control. The results of statistical analysis were shown three independent replicates. Values are expressed as means ± SEM. ^#^P < 0.01 versus LV-NC group; *P < 0.05 versus IH + LV-NC group. **(C)** Representative confocal microscopic images of gene-modified BV2 cells co-localization with Parkin (red) and TOM20 (green). Scale bar = 25 μm. **(D)** Representative TEM images of mitophagosomes (red arrow) in BV2 cells after IH exposure. Scale bar = 1 μm. IH, intermittent hypoxia; NA, normal air; LV, lentivirus; TEM, transmission electron microscopy.

To address how NLRP3 inflammasome modulates IH-induced mitophagy, we then verified the mitophagy and autophagy activities after NLRP3 deletion by western blot. As compared to IH exposure alone, deletion of NLRP3 dramatically restored the levels of Parkin approximately 1.5-fold over basal levels. Conversely, silencing NLRP3 exerted an inhibitory effect on the P62 expression under normoxia, and further reduced P62 expression after exposure to IH (**Figures 6A, B**). Next, we performed immunofluorescence staining of Parkin (red) and TOM20 (green) to evaluate the degree of Parkin-mediated mitophagic activity. As the enhanced immunofluorescent co-staining shown in **Figure 6C**, Parkin was observed to be translocated on damaged mitochondria, thereby resulting in substantial mitophagy following IH exposure. In particular, abundant Parkin expression was prominently localized on the mitochondria in NLRP3-deficient cells after IH treatment, suggesting the accumulation of mitophagosomes. Moreover, the positive effect of NLRP3 deficiency on parkin-mediated mitophagy could also be counteracted by 3MA pre-treatment (5 mM). Then, we examine the effect of NLRP3 deletion on mitochondrial morphology under TEM. After IH exposure, mitochondria were observed to swell and loss of cristae in the matrix of microglial cells. Consistent with the results of immunofluorescence, more mitophagosome formations were noticed in NLRP3-deficient microglial cells after IH exposure compared to the IH exposure alone group (**Figure 6D**). Taken together, these results indicated that NLRP3 deficiency showed protective effect against IH *via* inducing Parkin-mediated mitophagy.

### Parkin-Dependent Mitophagy Is Involved in the Protective Mechanism of NLRP3 Deficiency

**Figure 7:**
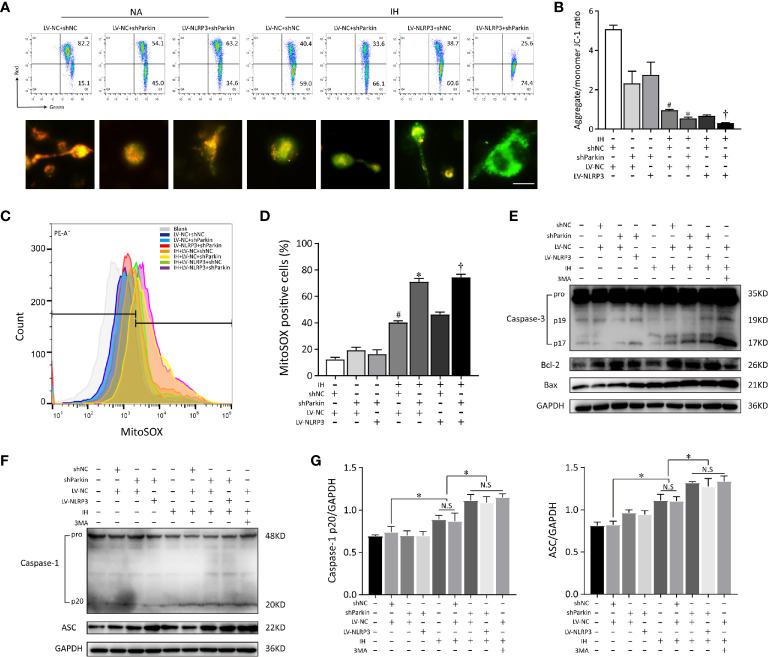
Parkin knockdown reverses the positive effect of NLRP3 deletion on mitochondrial maintenance in microglia. Gene modified BV2 cells transfected with sh-Parkin or pretreated with 3MA (5 mM) for 6h before exposure to IH. **(A)** Flow cytometry and immunofluorescent staining reflected the MMP. Scale bar = 50 μm. **(B)** Quantification of MMP changes was represented as the ratio of red to green fluorescence. **(C)** Parkin sh-RNA transfected LV-NC or LV-NLRP3 cells were stained with MitoSOX. **(D)** The quantitative histograms from the obtained results. ^#^P < 0.05 versus LV-NC + shNC group; *P < 0.05 versus IH + LV-NC + shNC group; ^†^P < 0.05 versus IH + LV-NC + shNC group. **(E)** Western blot analysis revealed that Parkin knockdown or pretreated with 3MA exacerbated the apoptosis that caused by IH, and neutralized the positive effect of NLRP3 knockout. **(F, G)** Effects of Parkin knockdown or 3MA pretreatment on cleaved caspase-1 and ASC protein expressions in gene modified BV2 cells exposed to IH. Representative histograms to quantify the relative levels and GADPH acted as an internal control. Similar results were obtained from three independent experiments. Data are presented as the mean ± SEM. *P < 0.05. NC, negative control; NS, not significant; IH, intermittent hypoxia; NA, normal air.

To clarify whether Parkin is required in the positive effect of NLRP3 deletion on mitochondrial maintenance in microglia, we first deleted Parkin *via* shRNA. After Parkin knockdown, IH enhanced the accumulation of damaged mitochondria and neutralized the protective of NLRP3 deletion in microglia, evidencing by increasing mitochondrial depolarization (red to green ratio, 0.34 ± 0.11%) and augmenting mtROS release (79.47 ± 0.15%), as illustrated in **Figures 7A–D**. Thus, proper control of Parkin-dependent mitophagy is pivotal to restoration of mitochondrial integrity and function in microglial cells after NLRP3 knockout. To further discuss the role of Parkin-dependent mitophagy in NLRP3^-/-^ microglia, we incubated BV2 cells with 3-MA (5 mM) or the PBS vehicle for 6h before exposure to IH. As shown in **Figure 7E**, both Parkin deletion or pretreated with 3MA exacerbated the cell apoptosis in NLRP3^-/-^ microglial cells caused by IH. Furthermore, Parkin deletion or 3MA pretreatment upon IH exposure resulted in higher expression levels of caspase-3 p17 and Bax compared with IH control group. Moreover, inhibition of mitophagy by 3MA pretreatment had pronounced effect on NLRP3^-/-^ microglial cells that further increased the protein expressions of NLRP3, ASC, pro-caspase-1 under IH condition compared to the IH control group (p < 0.05). The similar effect was observed in Parkin shRNA-transfected NLRP3^-/-^ cells (p < 0.05, **Figures 7F, G**). These results collectively indicated that Parkin deficiency failed to elicit a protective phenotype in the context of IH after NLRP3 knockout.

**Figure 8:**
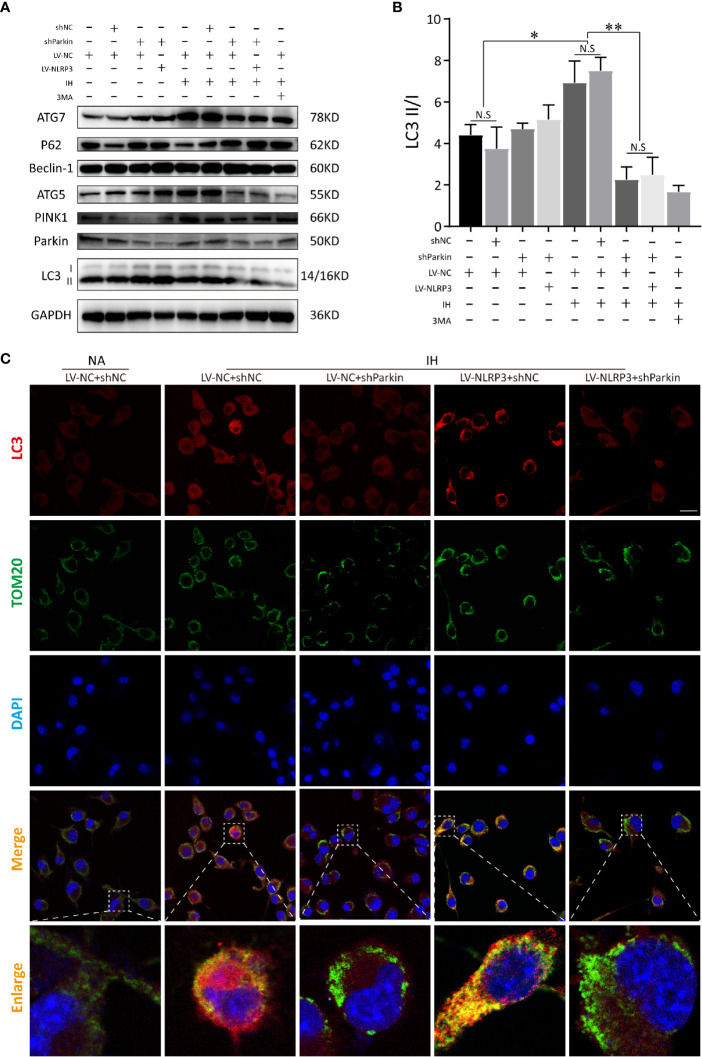
Parkin knockdown blocks the activation of mitophagy inducing by NLRP3 deletion in IH-treated microglia. **(A)** Immunoblot showed that after NLRP3 deletion, Parkin knockdown or 3-MA (autophagy inhibitor) pretreatment restrained the autophagy and mitophagy expressions (LC3-II, Beclin-1, ATG-5, ATG-7, Parkin, and PINK1), but upregulated P62 under IH condition in BV2 cells. **(B)** Quantification of autophagic flux was represented as the ratio of LC3 II to LC3 I proteins levels. Data are indicated as the mean ± SEM (n = 3 in each group). *P < 0.05; **P < 0.01; N.S, not significant. **(C)** Transfection with sh-Parkin inhibited the co-localization with TOM20 (green) and LC3 (red) upon IH challenge in NLRP3^-/-^ microglia. Scale bar = 25 μm. IH, intermittent hypoxia; NA, normal air; LV, lentivirus.

Next, we detected the mitophagic activities in NLRP3^-/-^ microglial cells after deletion of Parkin. Immunoblot analysis showed that silencing Parkin restrained the subsequent PINK1 mitophagy signaling under IH exposure and reversed the protective effect of NLRP3 deletion on IH-treated microglial cells, indicating the induction of mitophagy upon NLRP3 deficiency is Parkin-dependent (**Figures 8A, B**). Similarly, co-localization of LC3 (red) and TOM20 (green) demonstrated that Parkin ablation effectively prevented mitophagosomes formation upon IH challenge, evidenced by the weak red fluorescence of LC3 co-localized with TOM20. Moreover, after transfection with sh-Parkin, NLRP3 knockout no longer restored the impaired mitophagy after IH treatment. As expected, co-staining of LC3 and TOM20 showed faint double immunofluorescence in IH+LV-NLRP3+sh-Parkin group, as illustrated in **Figure 8C**. Taken together, our data showed that Parkin-dependent mitophagy plays a vital role in the NLRP3-deficient protective action under IH exposure, and inhibition of mitophagy *via* Parkin deletion abolished the positive effect of NLRP3 deficiency against IH (**Figure S2**).

## Discussion

At present, little is known about the mechanisms of structural neuron damage and the potential roles played by microglia during IH exposure from OSA. In our work, CIH elicited pathologies such as hippocampal apoptosis with learning deficits, while genetic deletion of NLRP3 displayed less neuronal damage or microglia activation after 5 weeks of CIH. In CNS, microglia are a robust source of oxidative stress, production of which are critical for self-activation of microglia and the overproduction of proinflammatory factors (6). As expected, we demonstrated that the microglia activation enhanced by CIH was accompanied by the elevated autophagy and mirophagy markers, LC3 and Parkin expressions *in vivo* experiments. In addition, we also indicated that NLRP3 deficiency can further enhanced Parkin-mediated mitophagy in hippocampus of IH mice, as well as autophagosomes formation in microglia of hippocampus. Of note, our data showed that CIH stimulated damaged mitochondria to release signals, such as mtROS and mtDNA, which further promoted the NLRP3 inflammasome complex assembly to activate the caspase‐1 and the subsequent cytokines IL‐1β release. The accumulation of proinflammatory cytokines as well as mitochondrial ROS was proposed to directly induce neuronal apoptosis, resulting in hippocampal-dependent impairment of learning and memory.

The major limitation of this article is that there is no clinically relevant data; however, some of researchers have provided clear evidences to show that neurocognitive deficit is identified as one of the main co-morbidities associated with OSA (4). Besides, previous studies also indicated the processes involved in the cognitive decline in patients with OSA were shown to overlap with those found in the pathogenesis of Alzheimer’s disease (AD) (3). Continuous positive airway pressure (CPAP), the first-line treatment for OSA patients, can rapidly improve the oxyhemoglobin desaturation and cognitive function of them (27). After a short-term CPAP treatment, functional MRI was used to show that OSA patients revealed an improvement in memory and attention (28), which changes were associated with that in cerebellar cortices and bilateral hippocampi (29). Taken together, these data indicated that OSA patients were usually suffered from cognitive impairment, and improving oxygen saturation can alleviate the phenomenon through regulating hippocampal function.

In line with our findings, Racanelli et al. reported chronic hypoxia triggered autophagosome formation (30). Recently, receptor-mediated mitophagy was found to be activated in response to hypoxia or mitochondrial oxidative stress (31). A previous study has also documented that level of cleaved caspase-3 and LC3 in hippocampal neurons were both upregulated by IH (32). Consistently, our results indicated that hypoxia initiated a time-dependent mitophagy activation whose ultimate goal was clearance of damaged mitochondria in BV2 cells. Meanwhile, IH-treated WT cells seemed more likely to apoptosis compared to NLRP3 knockout cells. In contrast, another study revealed that neuroinflammation activated the NLRP3-caspase-1 inflammasome in the hippocampus of mice and BV2 cells by triggering autophagy-lysosomal dysfunction, thus having specific relevance to neuronal cells damage (33). A possible mechanism has been proposed that a low ROS level specifically induces mitophagy without nonspecific autophagy, whereas excessive oxidative stress activates both autophagy and mitophagy as a negative-feedback to reduce mitochondria-derived ROS production (31). Actually, less severe protocols may elicit beneficial (compensatory) plasticity without morbidity (2). This diverse effect of IH on autophagy could in part be explained by the varying degrees of hypoxic paradigms: hypoxic events can be either neuroprotective or neurotoxic depending on the severity, frequency, and duration of the hypoxia.

Many researchers have attempted to link Parkin-PINK1 pathway to NLRP3 inflammasome. Several studies have reported that Parkin deficiency enhanced the production of inflammatory cytokines such as MCP-1, TNF-α and NF-kB (34). In addition, some investigators have found the enhanced NLRP3 signaling in Parkin-deficient cells (35). Sumpter et al. demonstrated the Parkin-dependent mitophagy limited NLRP3 activation in peripheral macrophages and primary fibroblasts (36). Intriguingly, Parkin can be cleaved by caspase-1, thus contributing to the resultant excessive inflammation cell death and pyroptosis. Herein, we revealed that NLRP3 deletion attenuated IH-induced injury through enhancement of Parkin-mediated mitophagy. In addition, inhibition of mitophagy *via* parkin deletion was shown to facilitate the cell apoptosis and abolish the protective effect of NLRP3 deficiency against IH. These data indicate that Parkin-mediated mitophagy is one of the self-limiting systems to protect cells from hyper-inflammation. Although hypoxia-induced mitophagy *via* receptors such as Parkin/PINK1, Bcl2/adenovirus E1B 19 kDa protein-interacting protein 3 (BNIP3)/NIX, and FUN14 domain containing 1 (FUNDC1) has been described (34, 35), the crucial molecular mechanisms for hypoxia-induced mitophagy appeared to be cell type-specific. Herein, we only presented *in vitro* data that Parkin-dependent mitophagy controlled mitochondrial quality following hypoxia exposure. It would be interesting to further investigate the effect of NLRP3 deficiency on other mitophagy signaling.

Accumulation of misfolded proteins and damaged mitochondria has been documented to be hallmarks of neurologic disease (37). Coupled with lost membrane potential, most damaged mitochondria inhibit PINK1 imported to the inner mitochondrial membrane but stabilized on outer mitochondrial membrane (38). Then, the phosphorylated Parkin mediates mitochondrial ubiquitination, as an ‘eat-me’ signal that can be recognized by the adaptor p62 (21, 39). It is widely accepted that NLRP3 inflammasome stimuli could impair the mitochondria (26). By eliminating damaged mitochondria, mitophagy induction is dependent on recruitment of p62/SQSTM1 to limit inflammasome as a compensatory mechanism. Besides, damaged mitochondria further facilitate inflammasome inductive signals *via* mtDNA or mtROS, forming a vicious circle afterward (25). In the present study, protective effects of the NLRP3 deficiency on mitophagy and mitochondrial dysfunction have been undoubtedly identified. Furthermore, in IH-treated BV2 cells, we found p62 level to be dramatically suppressed compared to WT group, and further be restrained when NLRP3 deletion. This discrepancy is likely due to the excessive mtROS or NLRP3 inflammasome produced a large amount of autophagosome, caused overwork of lysosome, and subsequently resulted in failure of autophagy and mitophagy during CIH exposure. It accounts for the fact that decreased p62 levels represent the enhancement of autophagy flux, and the reason that NLRP3 deletion elicited more mitophagosomes formation attribute to the balance between the generation and elimination of harmful substances once again. Interestingly, some research found that p62 was dispensable for parkin-mediated mitophagy (39). Despite the controversial roles of P62, it is generally recognized that parkin recruits P62 to mediate mitophagy through selective cargo recognition. However, the inverse correlation between PINK1-Parkin pathway and P62 warranted to be explored in further study.

## Conclusions

In summary, our study revealed that NLRP3 ablation or inhibition orchestrated a reparative inflammatory response, which was linked with enhanced Parkin-dependent mitophagy upon hypoxia. As a regulatory feedback loop that maintains homeostasis and favors intrinsic repair in response to mitochondrial oxidative stress, Parkin-dependent mitophagy is involved in the protective mechanism of NLRP3 deficiency. Although, the directly molecular mechanism between NLRP3 and Parkin is still unclear, our results highlight the significant implication of NLRP3-Parkin axis as an important signaling pathway that determines the fate of cells under hypoxia. The mechanism of how *NLRP3* gene knockdown leads to the elevated Parkin protein level will be further studied in our subsequent experiments. Overall, our observations suggest the imbalanced crosstalk of NLRP3-Parkin axis participates the pathogenesis of CIH-induced neuroinflammation. Gene knockout or pharmacological blockage of NLRP3 might serve as a potential therapeutic target for OSA associated neurocognitive impairment.

## Data Availability Statement

The raw data supporting the conclusions of this article will be made available by the authors, without undue reservation.

## Ethics Statement

The animal study was reviewed and approved by the Medical Experimental Animal Administrative Committee of the Shanghai Medical College of the Fudan University.

## Author Contributions

XW and LG designed experiments and wrote the paper. XW, LG, LX, WG, and XYW performed experiments and acquired data. XW, LG, WG, XYW, ZL, and SL analyzed data and supervised the research. All authors contributed to the article and approved the submitted version.

## Funding

The study was financially supported by grants from The National Key Research and Development Program of China (No. 2018YFC1313600), the National Natural Science Foundation of China, Grant/Award Number: (No. 82070094, 82000095, 81900086, 81800089, & 81873420), the Shanghai Top-Priority Clinical Key Disciplines Construction Project, Grant/Award Number: (No. 2017ZZ02013), and the fund for Fundamental Research Funds for the Central Universities (22120180576).

## Conflict of Interest

The authors declare that the research was conducted in the absence of any commercial or financial relationships that could be construed as a potential conflict of interest.

## Supplementary Material

Supplementary Figure 1 Quantification of the relative protein (NLRP3, and Caspase-1 p20) levels in **Figure 1C**, and GADPH acted as an internal control. ^#^P < 0.01 versus NA group; **P < 0.01 versus CIH + WT group, ****P < 0.001 versus CIH + WT group.

Supplementary Figure 2 Schematic diagram of the interaction between PINK1/Parkin-dependent mitophagy and NLRP3 inflammasome in OSA-associated neuroinflammation. CIH induced mitochondrial ROS production, and facilitated NLRP3 inflammasome assembly to subsequently activate the caspase-1 cleavage. NLRP3 deficiency by gene knockout or pharmacological blockage could restore the CIH-induced mitochondrial dysfunction, alleviate mtROS production, reduce cell apoptosis, and further enhance Parkin-mediated mitophagosome formation. Green lines: facilitation. Red lines: inhibition. CIH, intermittent hypoxia.
