# Supplementary figures and images for: NLRP3 Deficiency Protects Against Intermittent Hypoxia-Induced Neuroinflammation and Mitochondrial ROS by Promoting the PINK1-Parkin Pathway of Mitophagy in a Murine Model of Sleep Apnea
Source: Front Immunol. 2021 Feb 24;12:628168. doi: 10.3389/fimmu.2021.628168 (PMC7943742; doi:10.3389/fimmu.2021.628168)

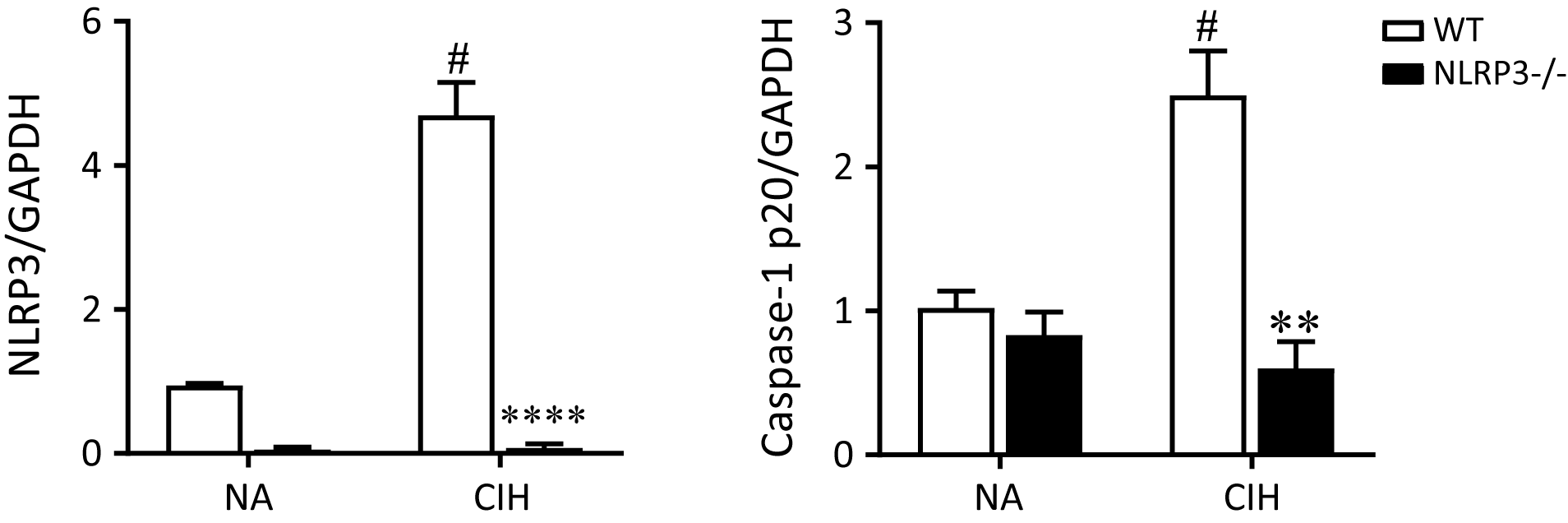

Supplement: Supplementary Figure 1 — Quantification of the relative protein (NLRP3, and Caspase-1 p20) levels in Figure 1C , and GADPH acted as an internal control. #P < 0.01 versus NA group; **P < 0.01 versus CIH + WT group, ****P < 0.001 versus CIH + WT group. [file Image_1.tif]

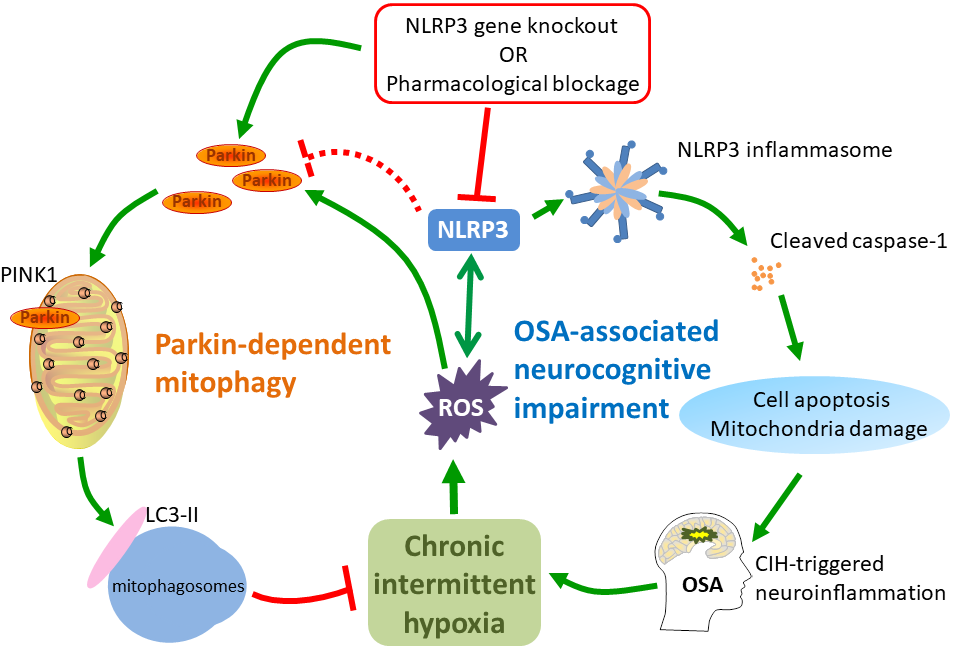

Supplement: Supplementary Figure 2 — Schematic diagram of the interaction between PINK1/Parkin-dependent mitophagy and NLRP3 inflammasome in OSA-associated neuroinflammation. CIH induced mitochondrial ROS production, and facilitated NLRP3 inflammasome assembly to subsequently activate the caspase-1 cleavage. NLRP3 deficiency by gene knockout or pharmacological blockage could restore the CIH-induced mitochondrial dysfunction, alleviate mtROS production, reduce cell apoptosis, and further enhance Parkin-mediated mitophagosome formation. Green lines: facilitation. Red lines: inhibition. CIH, intermittent hypoxia. [file Image_2.tif]

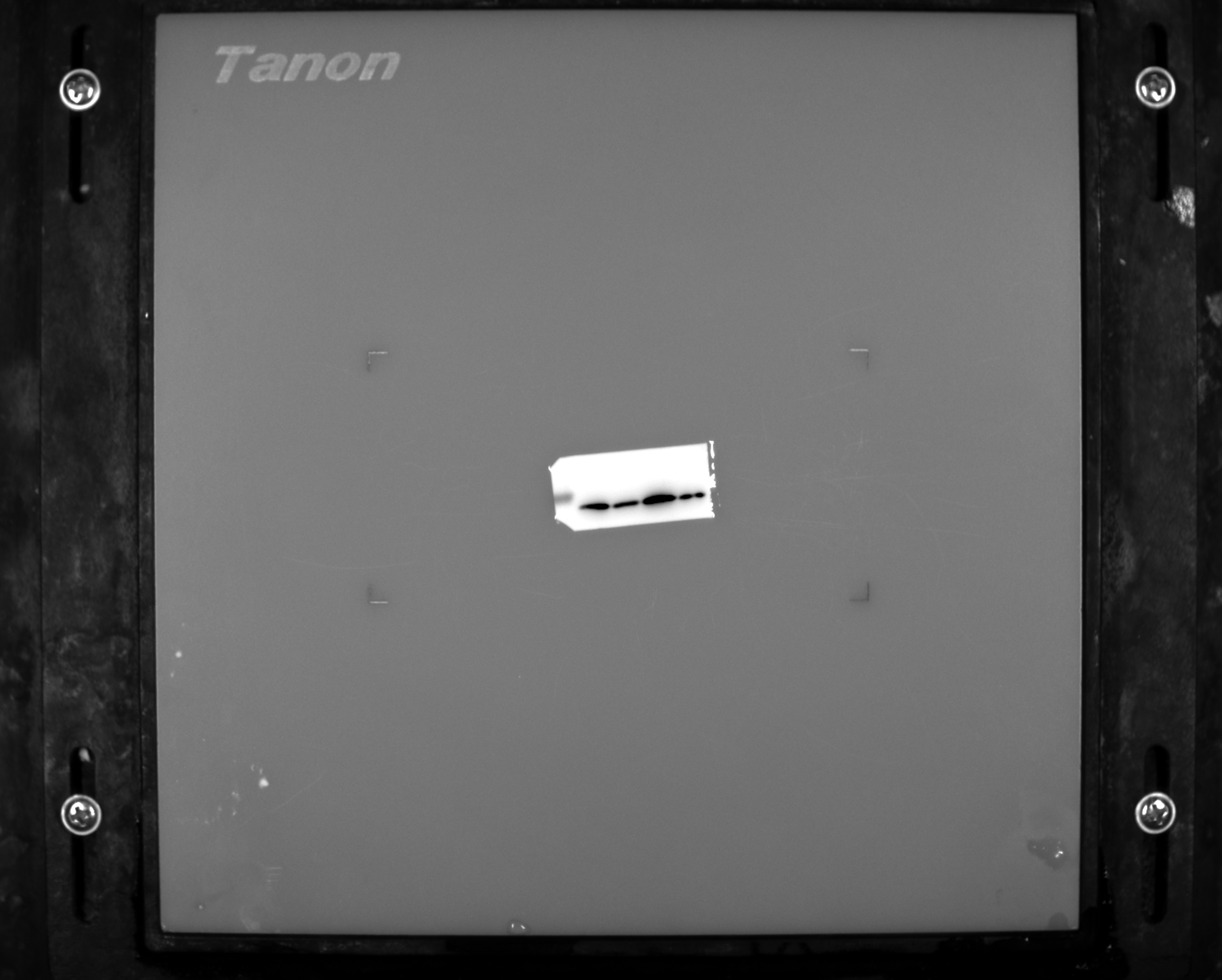

Supplement: Supplementary file 3 [file DataSheet_1.zip › Figure 1/Caspase-1 p20.Tif]

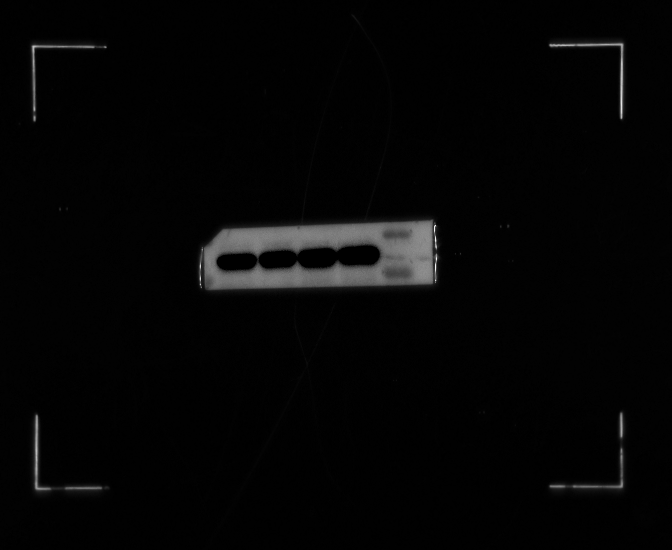

Supplement: Supplementary file 3 [file DataSheet_1.zip › Figure 1/GAPDH.tif]

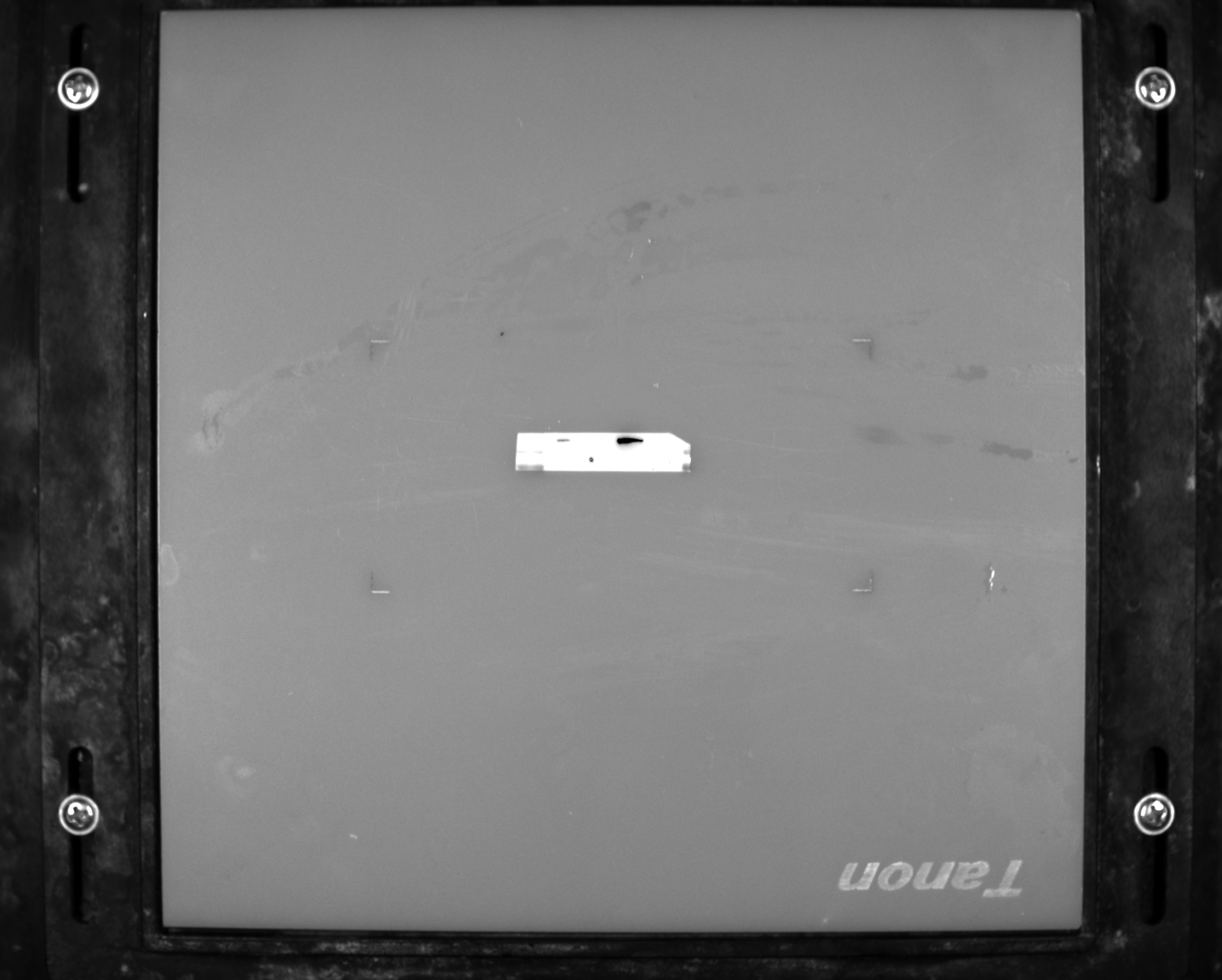

Supplement: Supplementary file 3 [file DataSheet_1.zip › Figure 1/NLRP3.Tif]

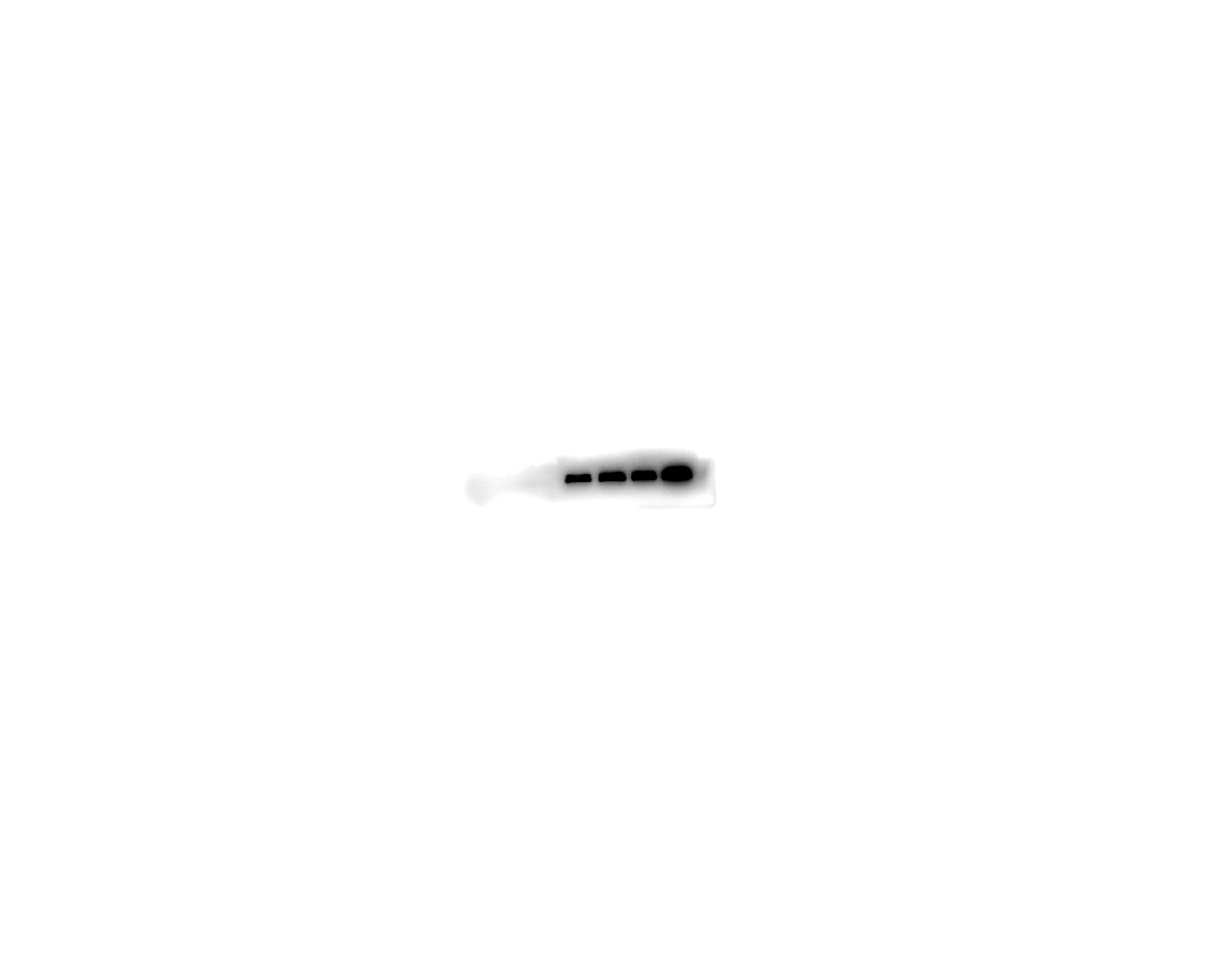

Supplement: Supplementary file 4 [file DataSheet_2.zip › Figure 3/Beclin-1.Tif]

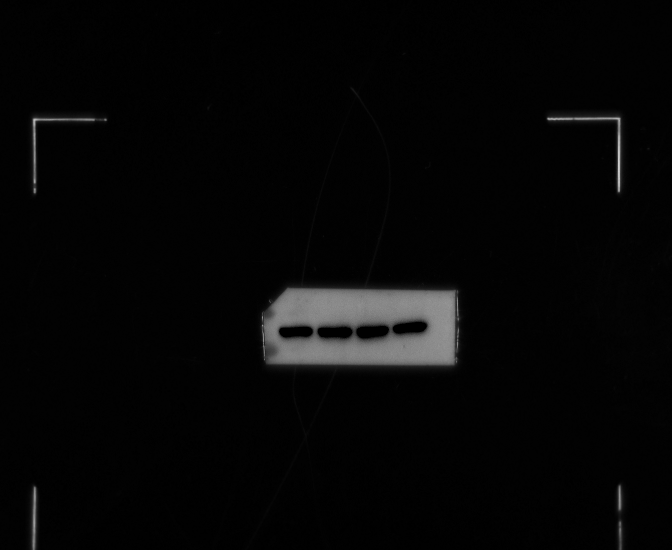

Supplement: Supplementary file 4 [file DataSheet_2.zip › Figure 3/GAPDH.tif]

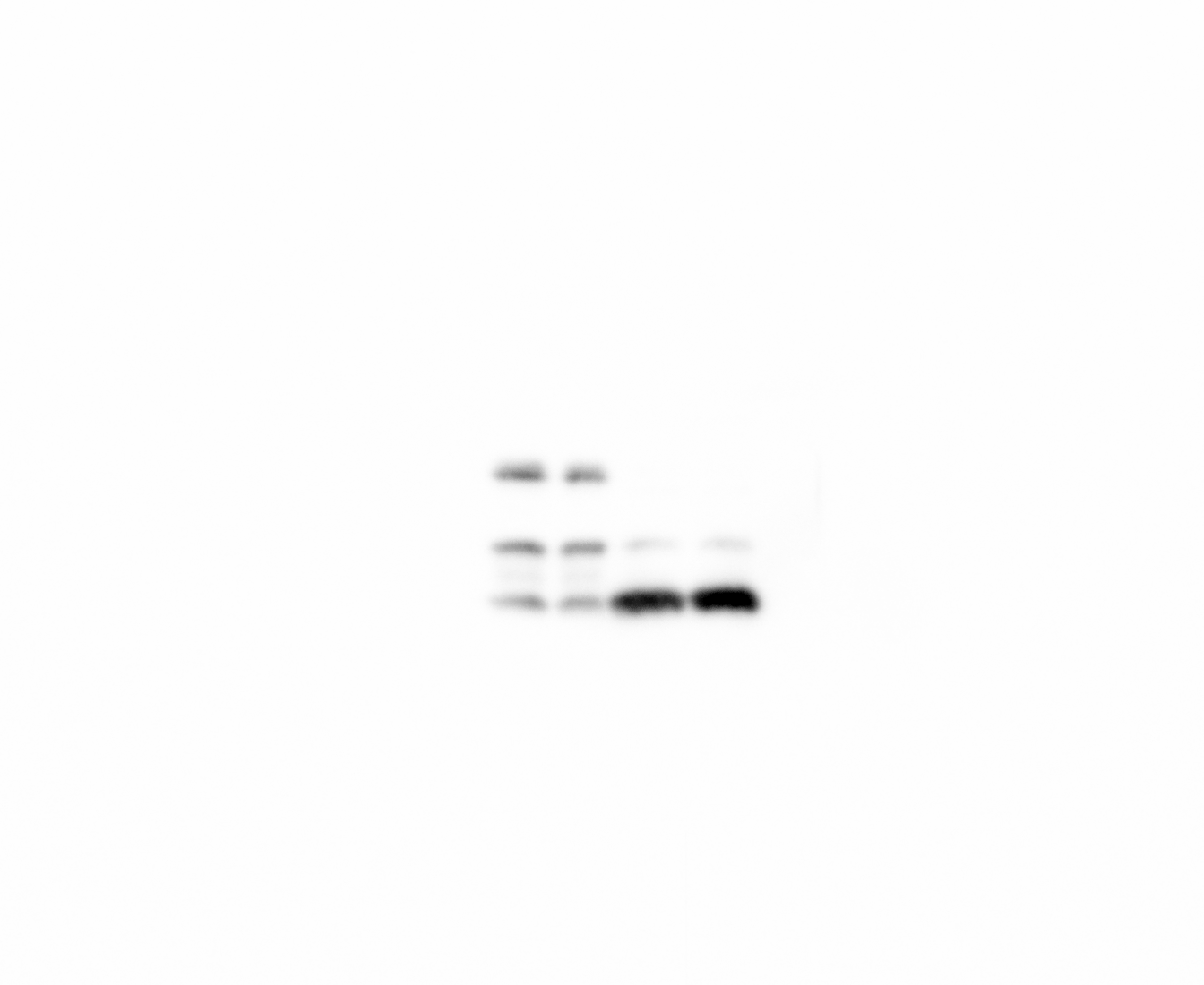

Supplement: Supplementary file 4 [file DataSheet_2.zip › Figure 3/LC3.tif]

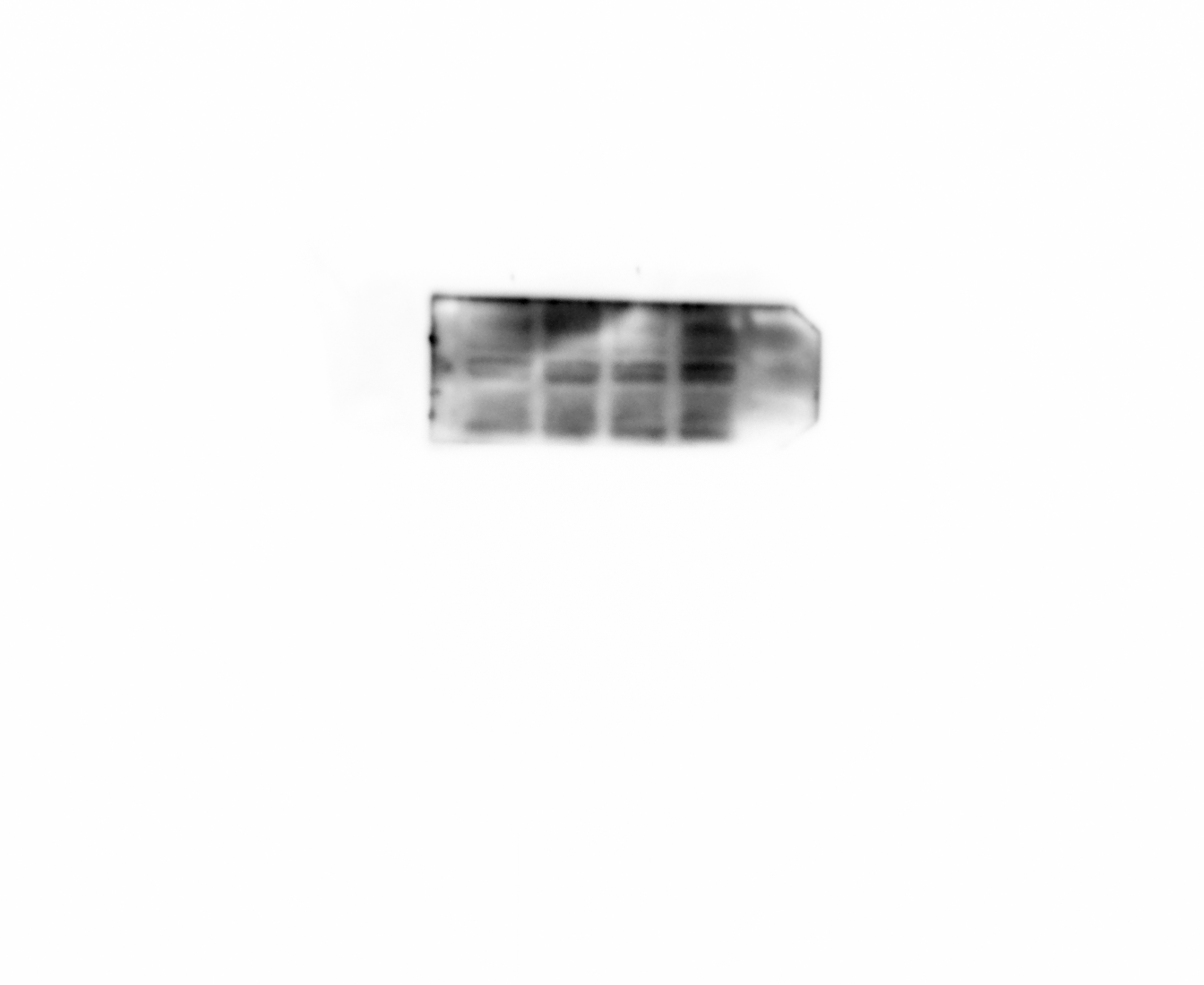

Supplement: Supplementary file 4 [file DataSheet_2.zip › Figure 3/Parkin.tif]

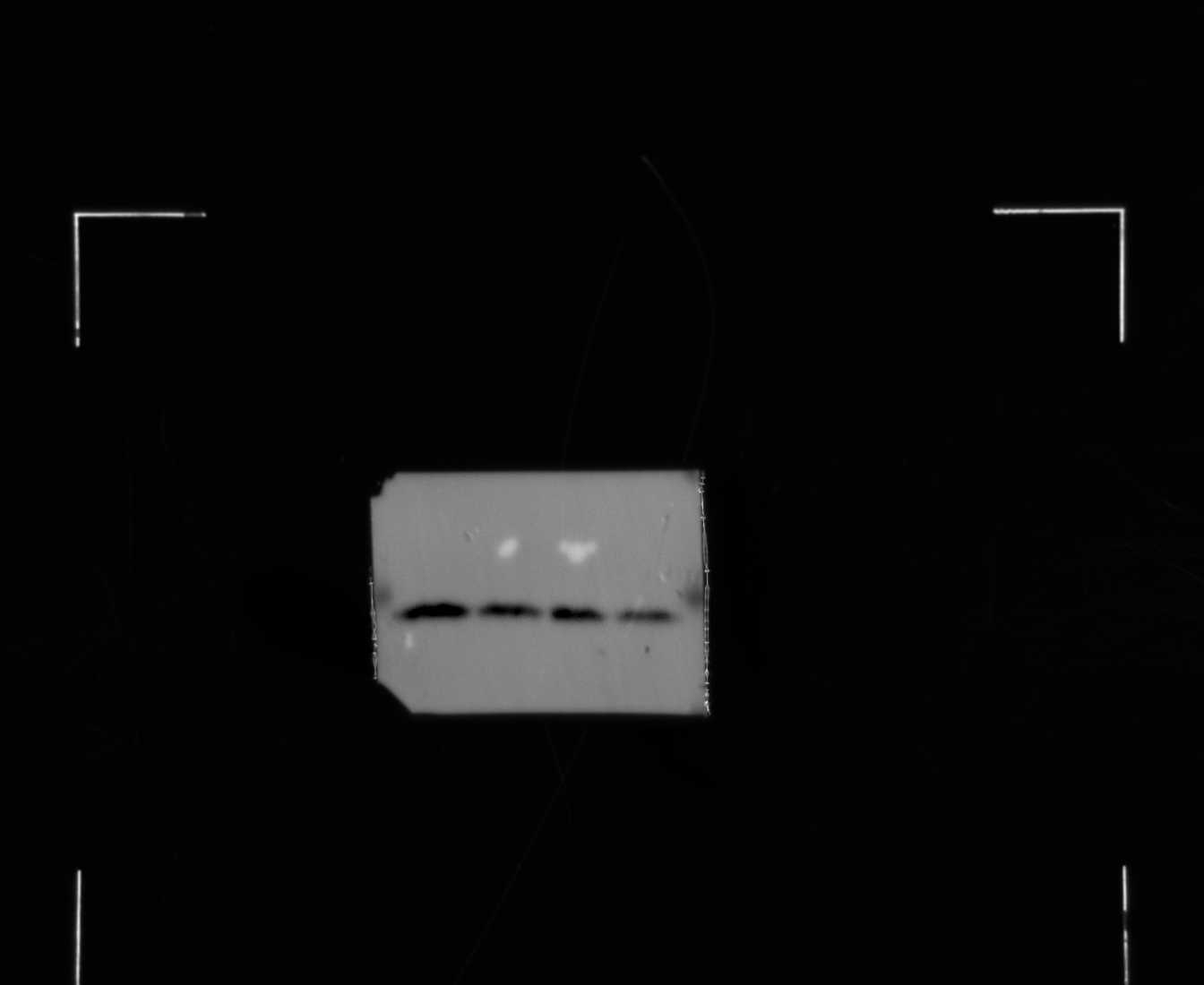

Supplement: Supplementary file 4 [file DataSheet_2.zip › Figure 3/TOM20.tif]

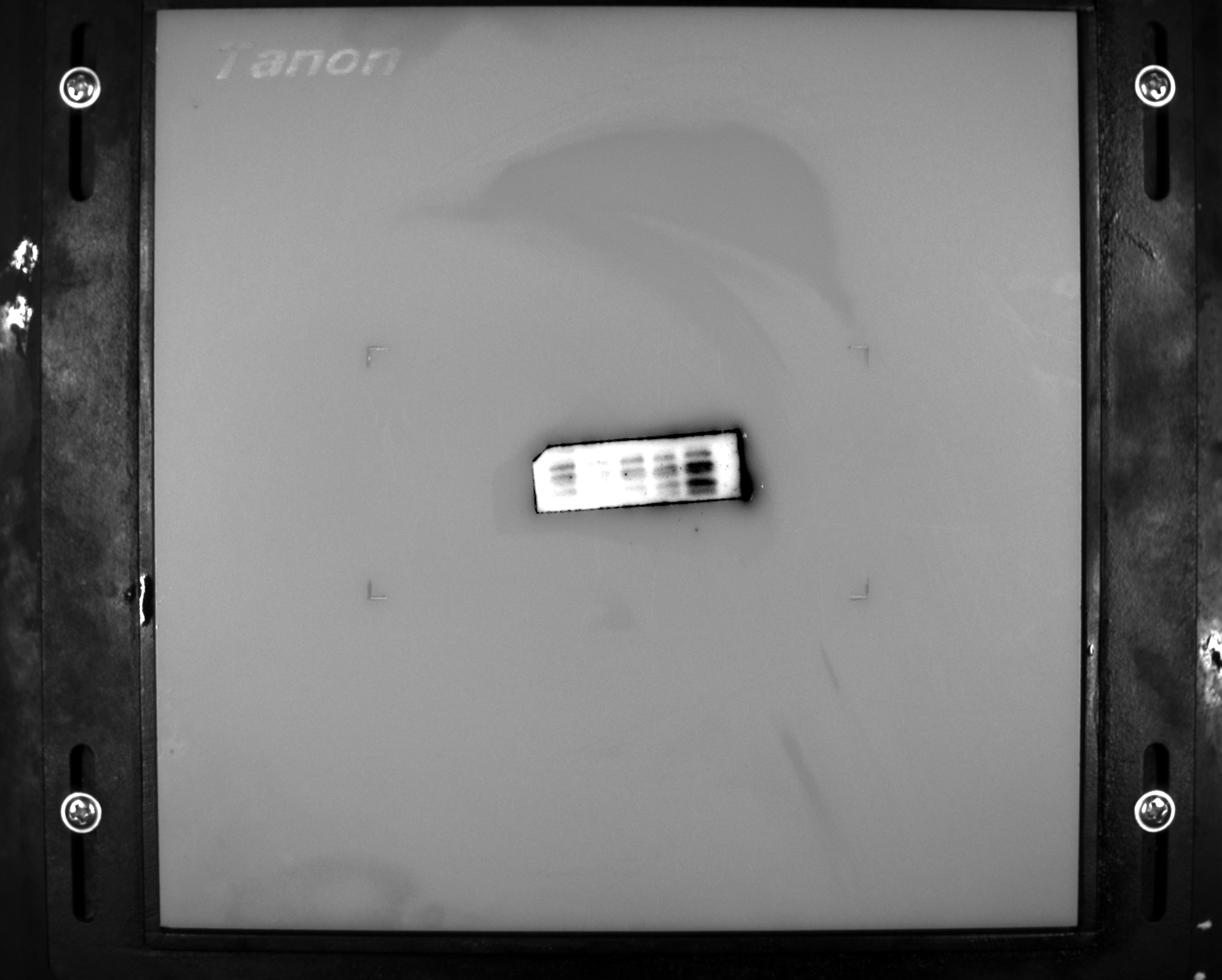

Supplement: Supplementary file 5 [file DataSheet_3.zip › Figure 4/Figure 4A/ASC.Tif]

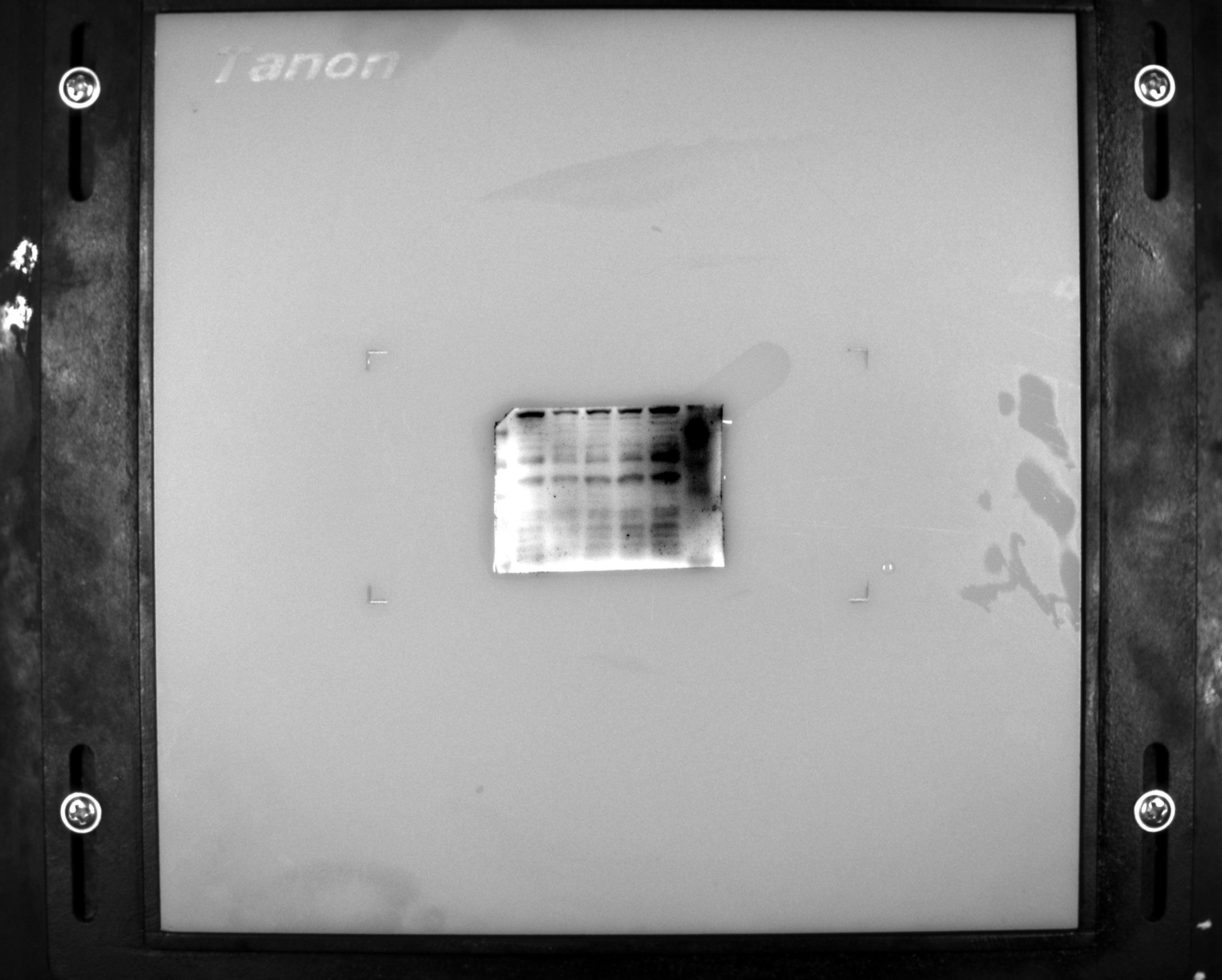

Supplement: Supplementary file 5 [file DataSheet_3.zip › Figure 4/Figure 4A/Caspase-1.Tif]

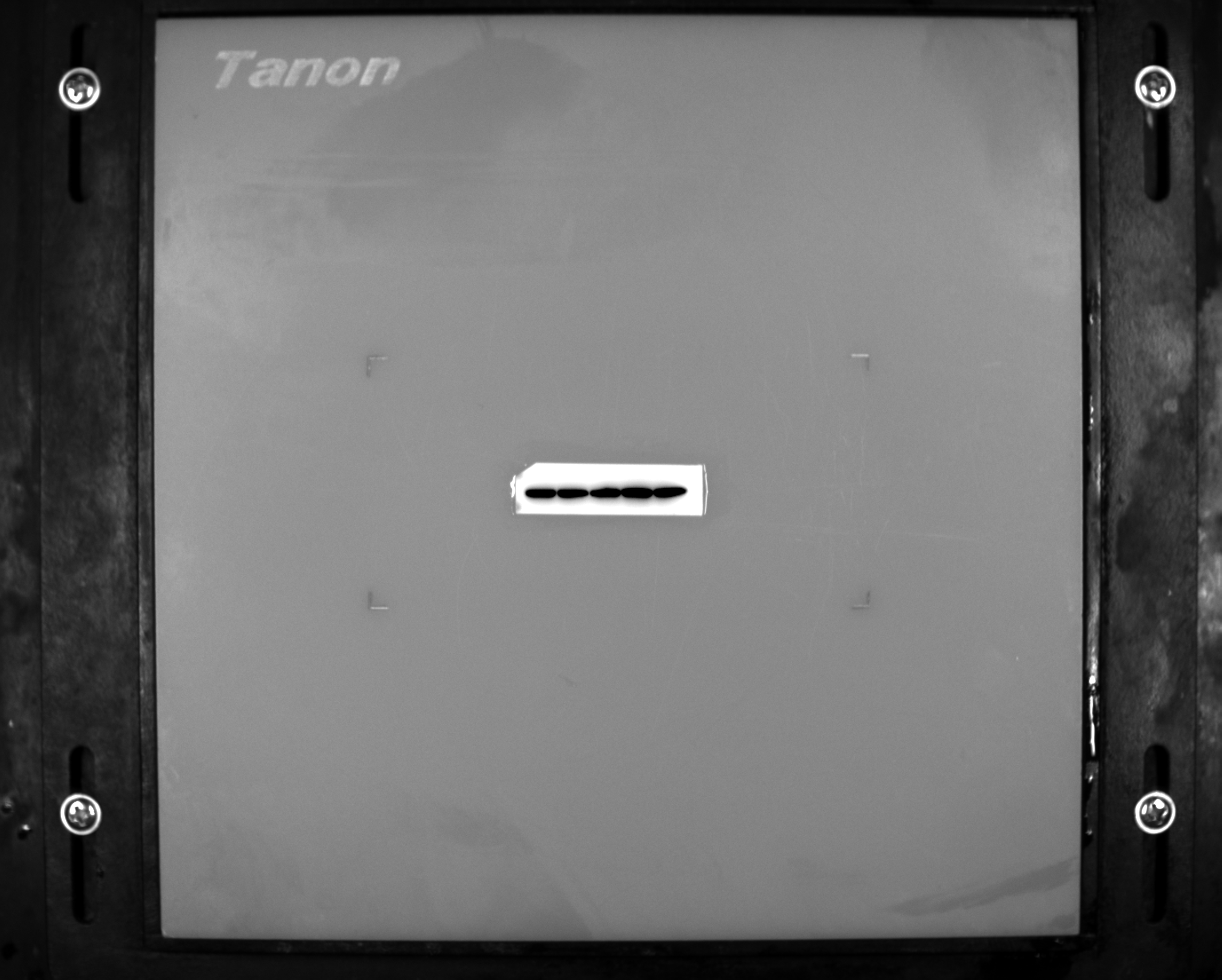

Supplement: Supplementary file 5 [file DataSheet_3.zip › Figure 4/Figure 4A/GAPDH.Tif]

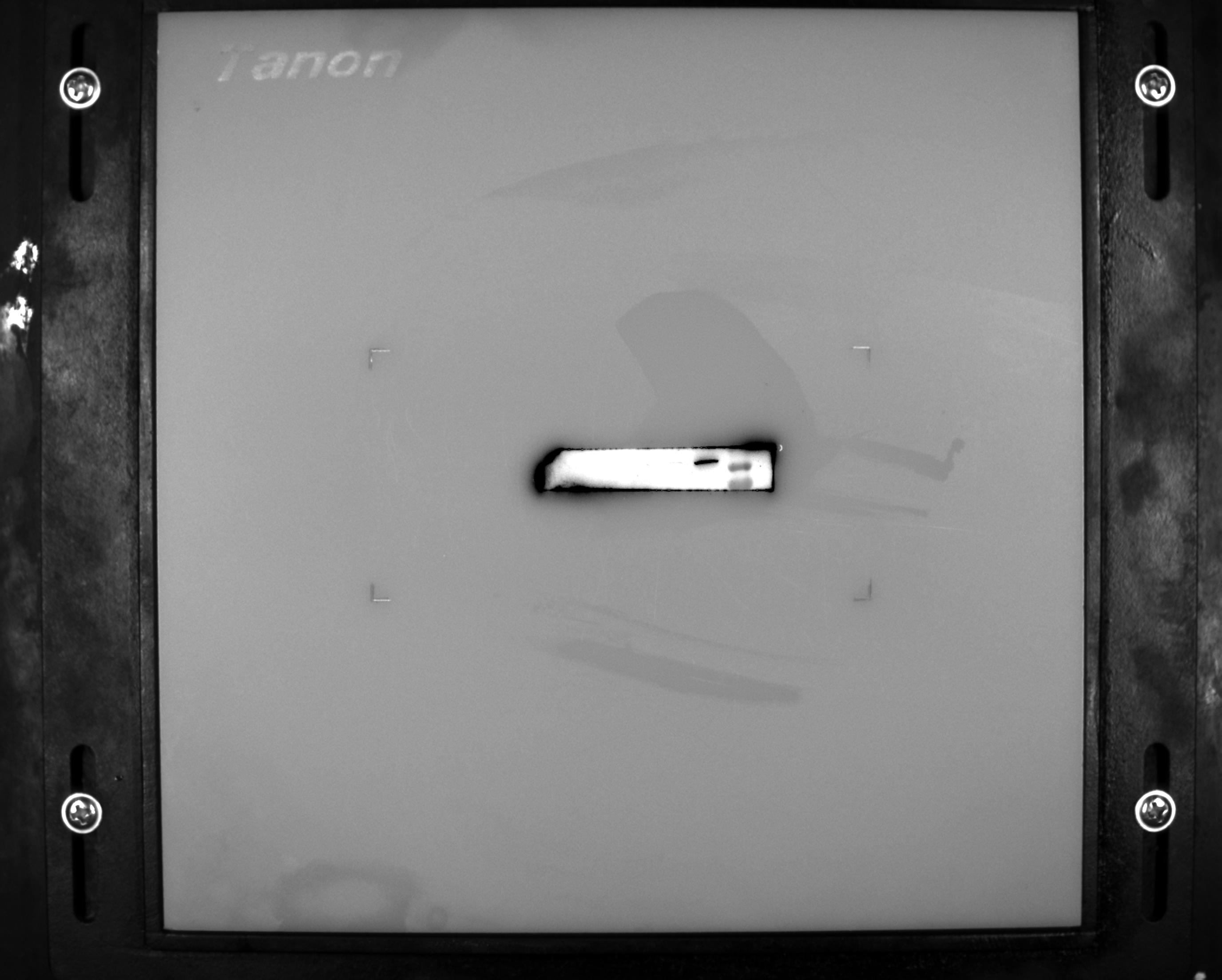

Supplement: Supplementary file 5 [file DataSheet_3.zip › Figure 4/Figure 4A/NLRP3.Tif]

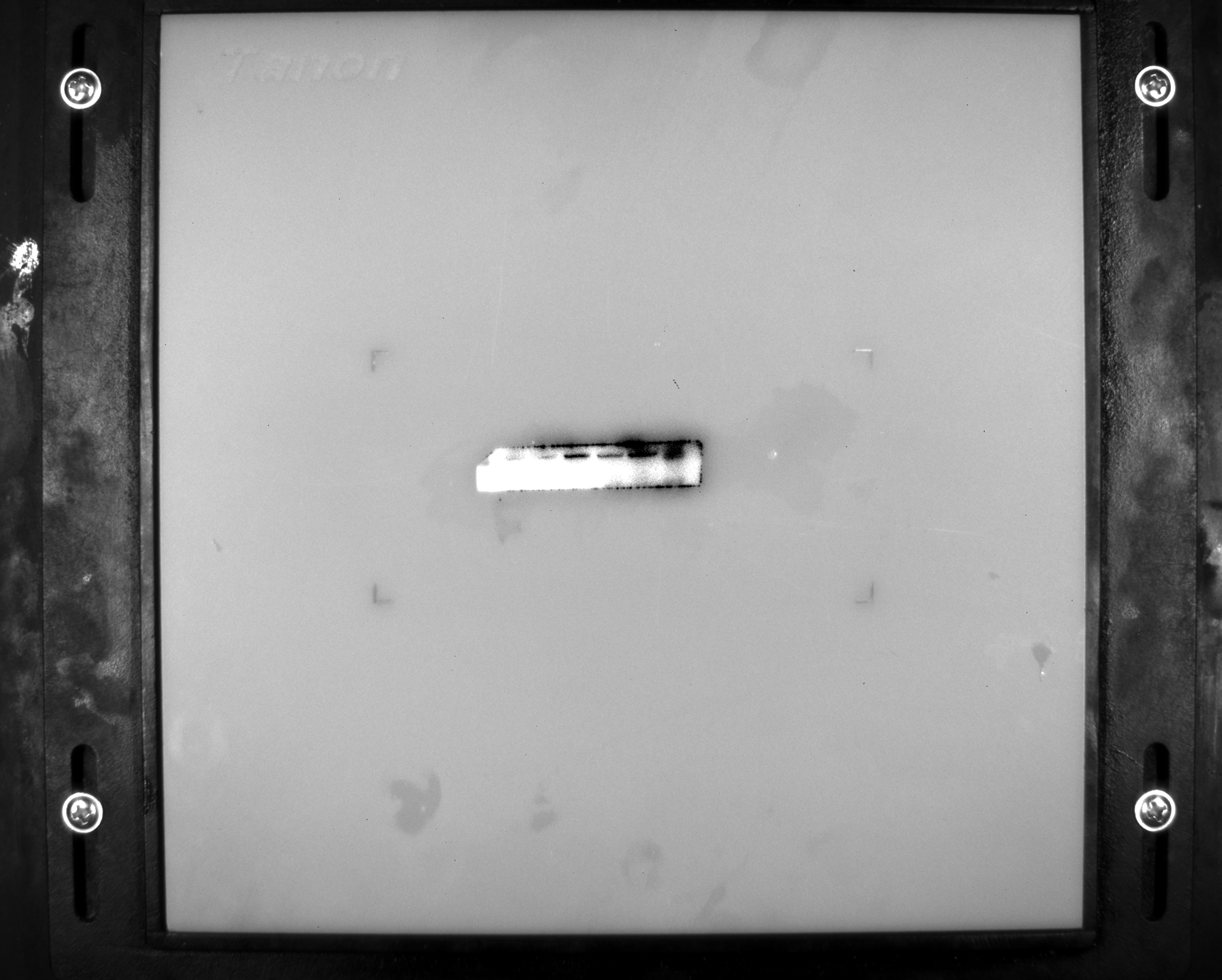

Supplement: Supplementary file 5 [file DataSheet_3.zip › Figure 4/Figure 4C/ATG5.Tif]

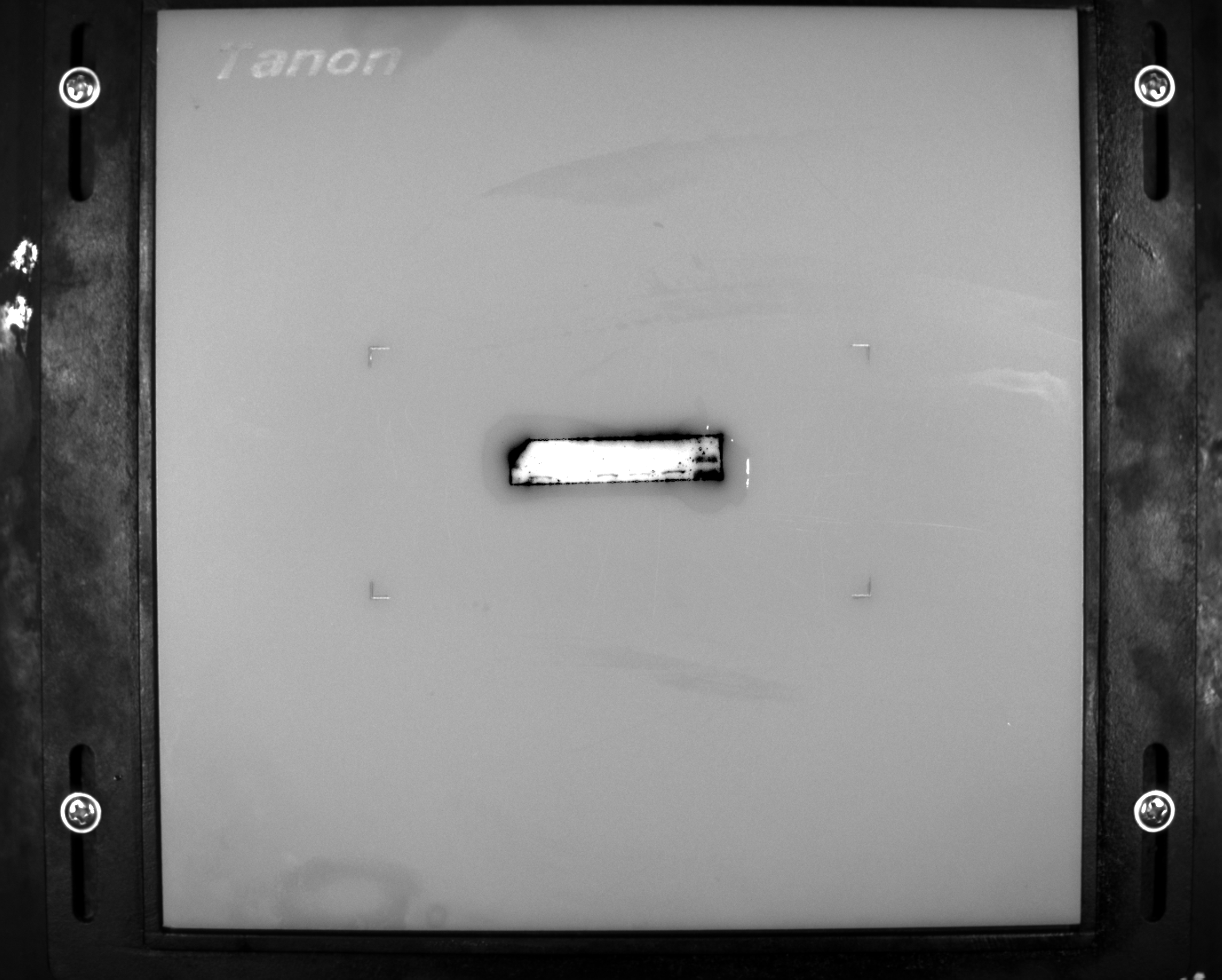

Supplement: Supplementary file 5 [file DataSheet_3.zip › Figure 4/Figure 4C/ATG7.Tif]

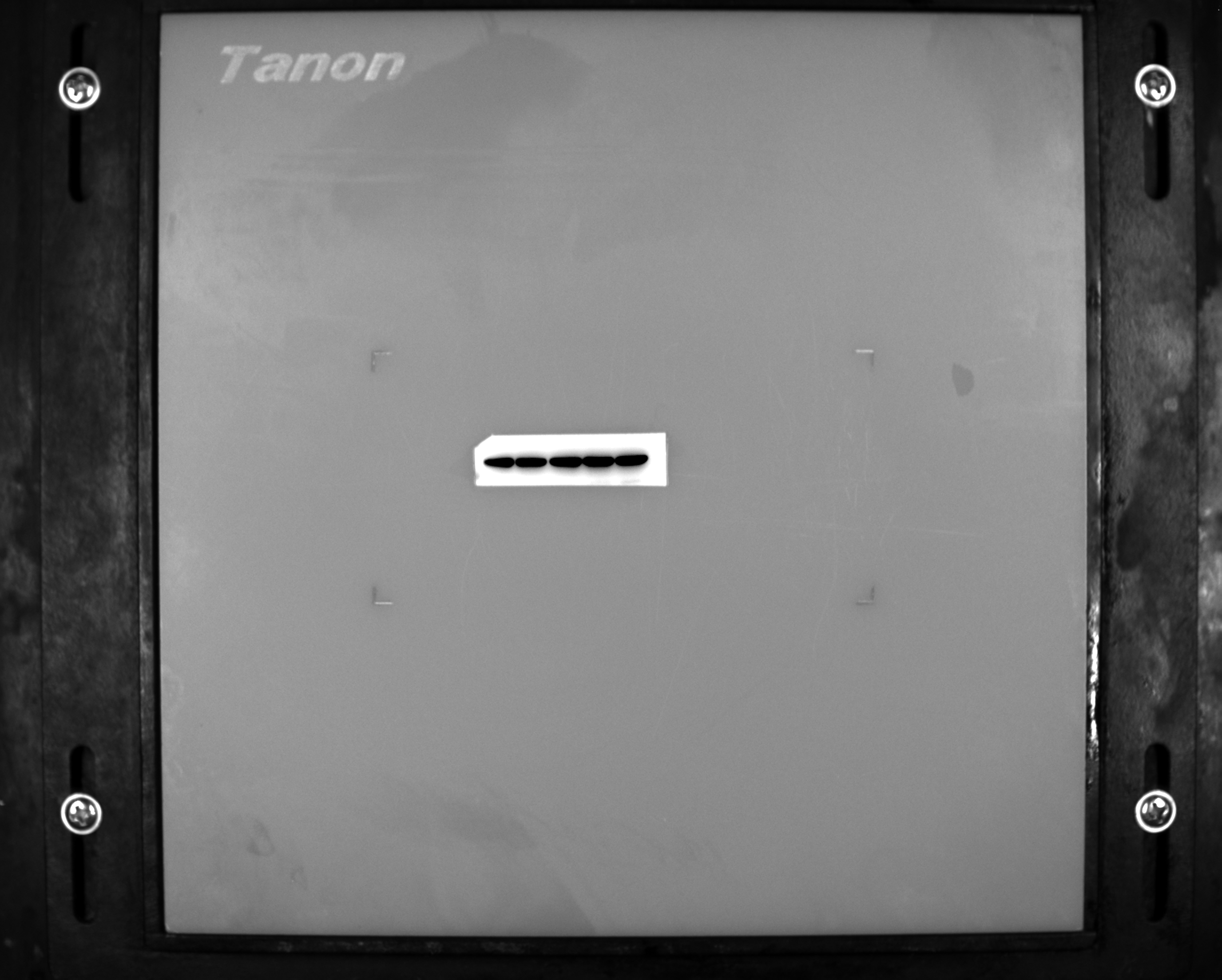

Supplement: Supplementary file 5 [file DataSheet_3.zip › Figure 4/Figure 4C/GAPDH.Tif]

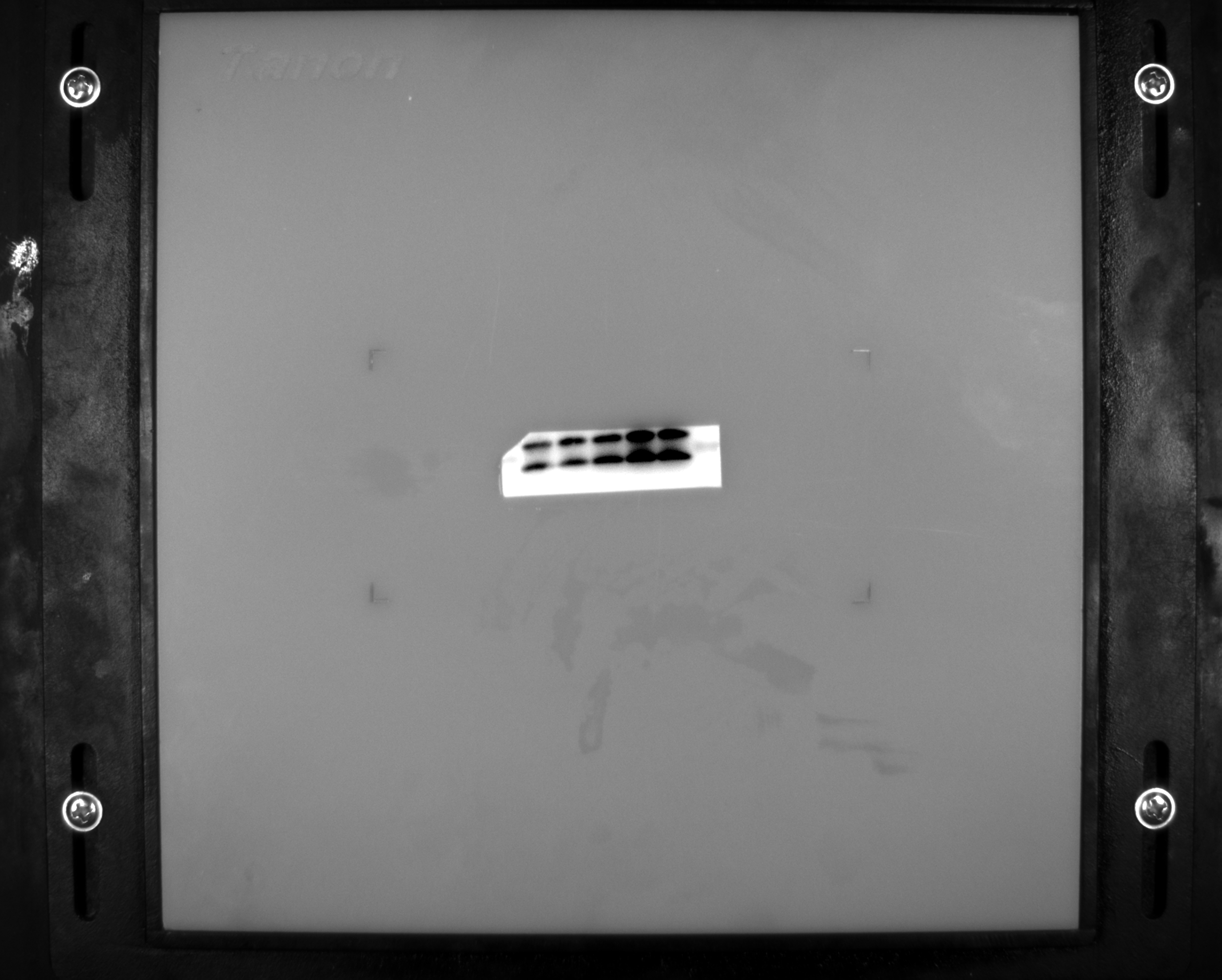

Supplement: Supplementary file 5 [file DataSheet_3.zip › Figure 4/Figure 4C/LC3.Tif]

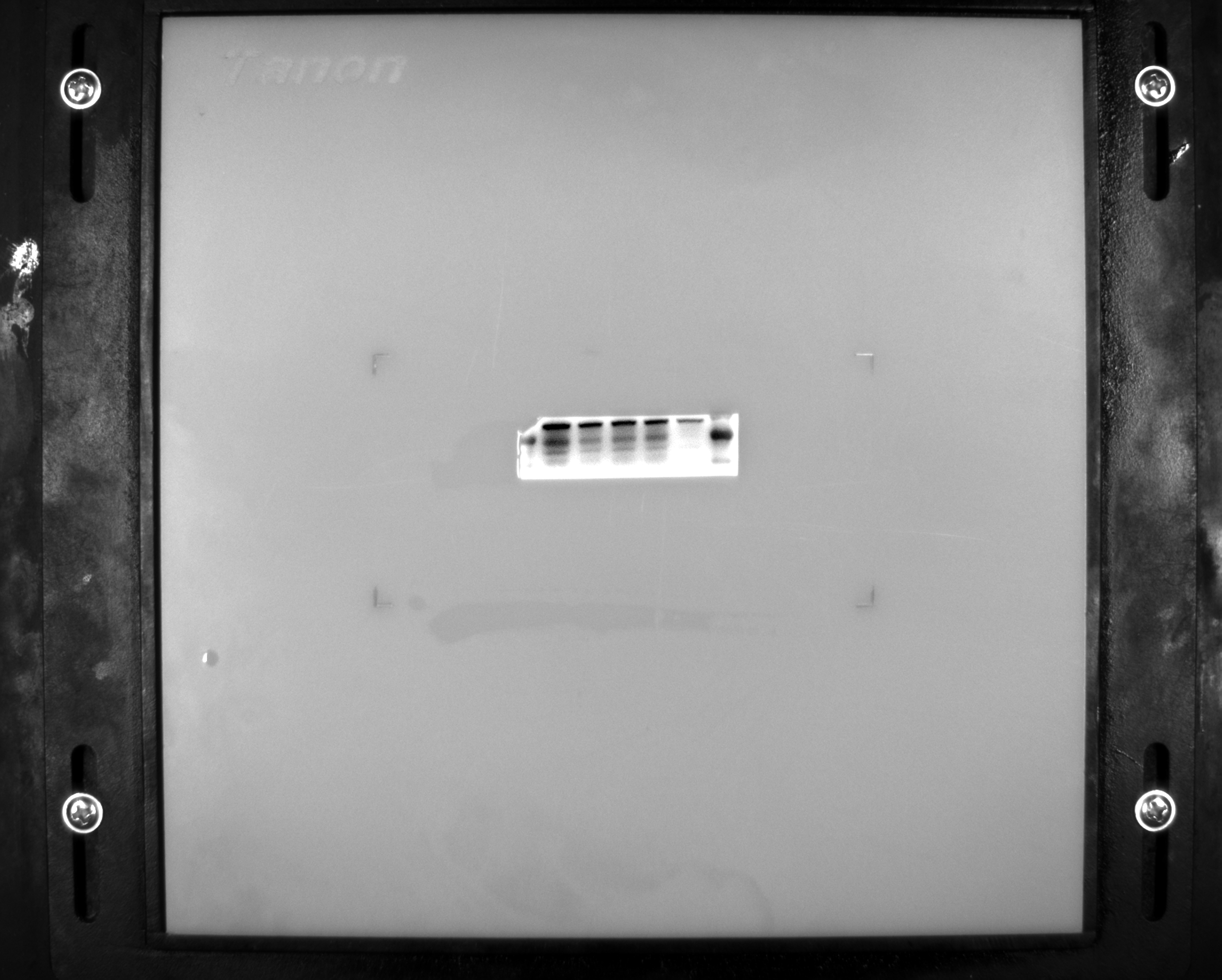

Supplement: Supplementary file 5 [file DataSheet_3.zip › Figure 4/Figure 4C/P62.Tif]

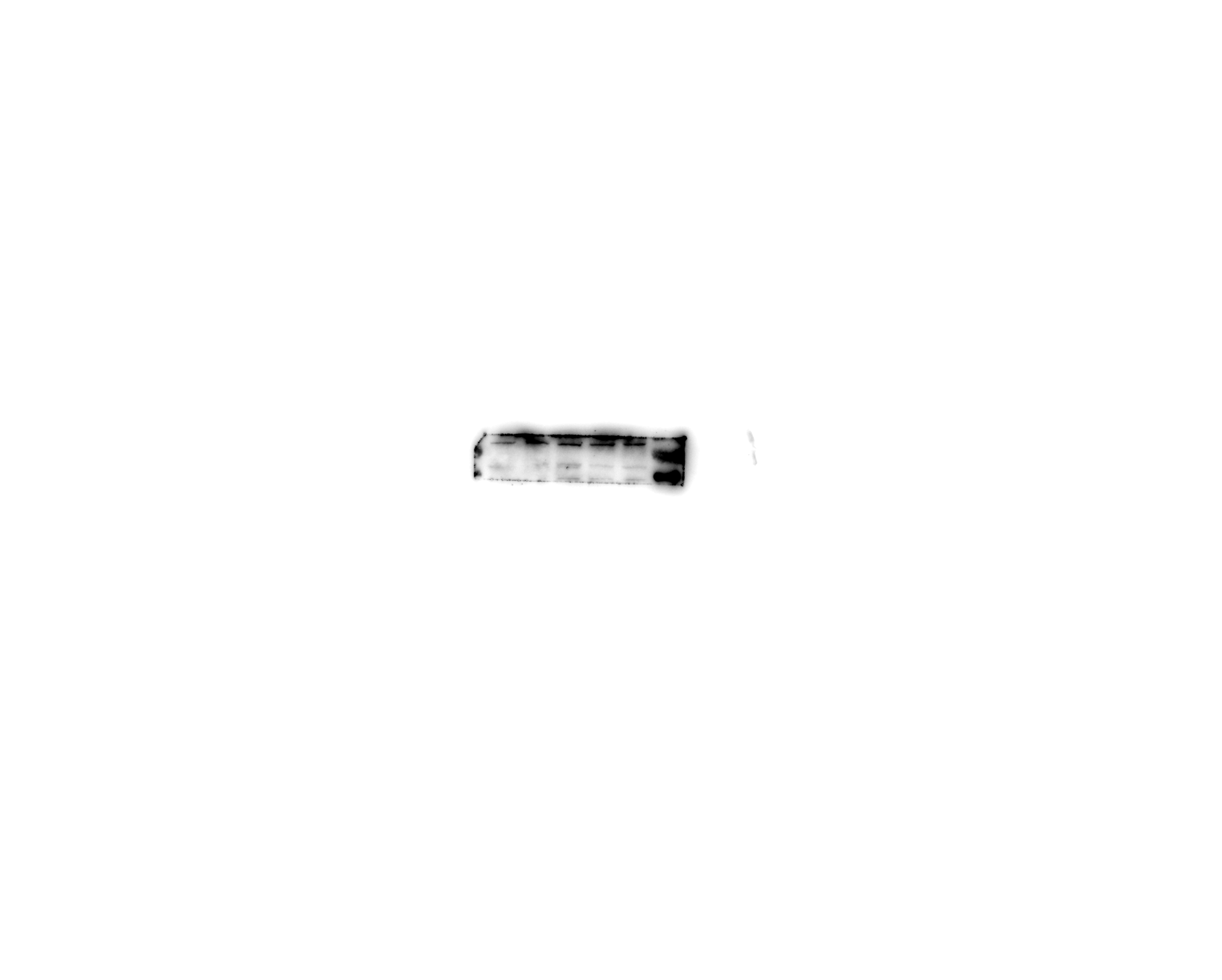

Supplement: Supplementary file 5 [file DataSheet_3.zip › Figure 4/Figure 4C/PINK1.Tif]

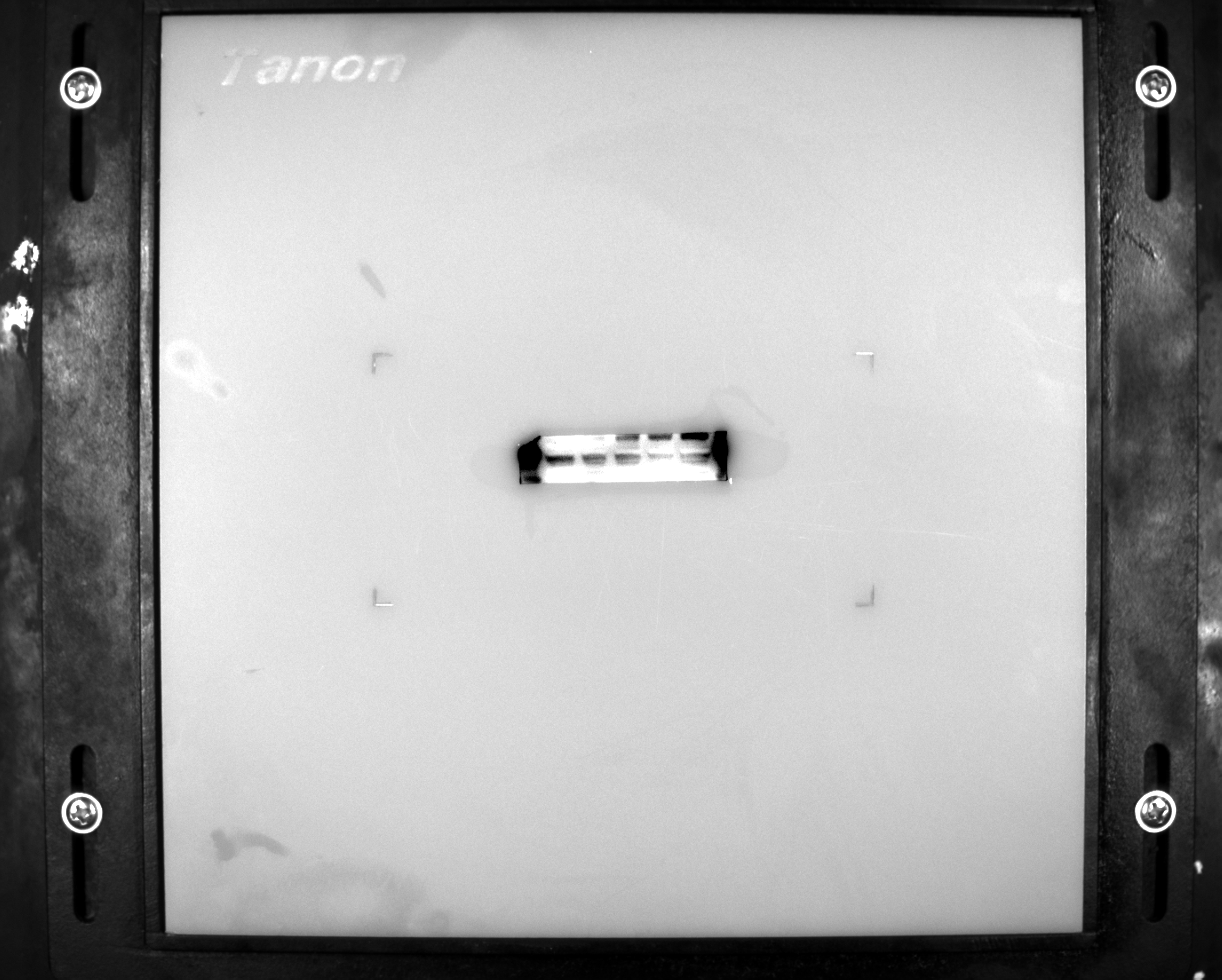

Supplement: Supplementary file 5 [file DataSheet_3.zip › Figure 4/Figure 4C/Parkin.Tif]

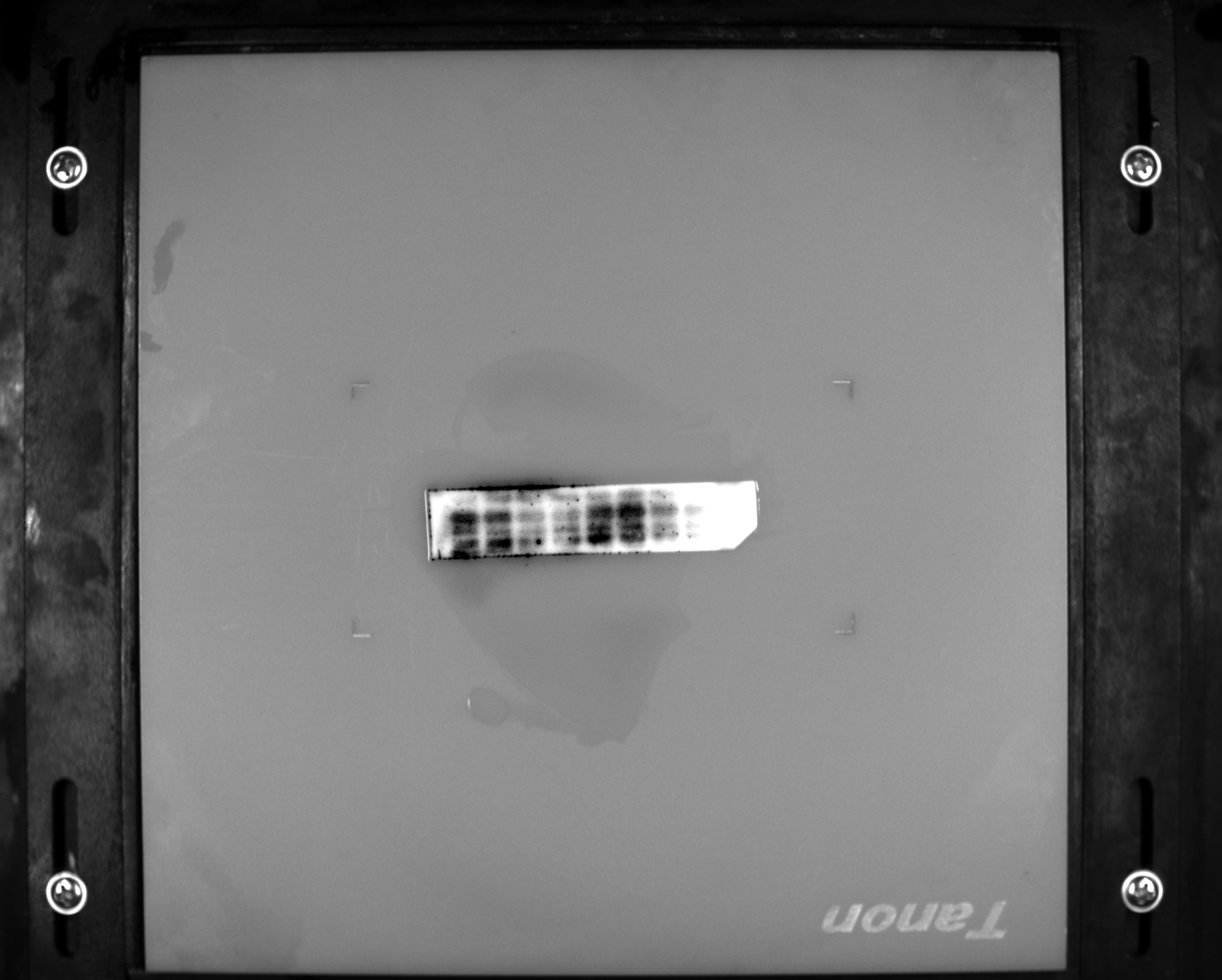

Supplement: Supplementary file 6 [file DataSheet_4.zip › Figure 5/Figure 5A/ASC.Tif]

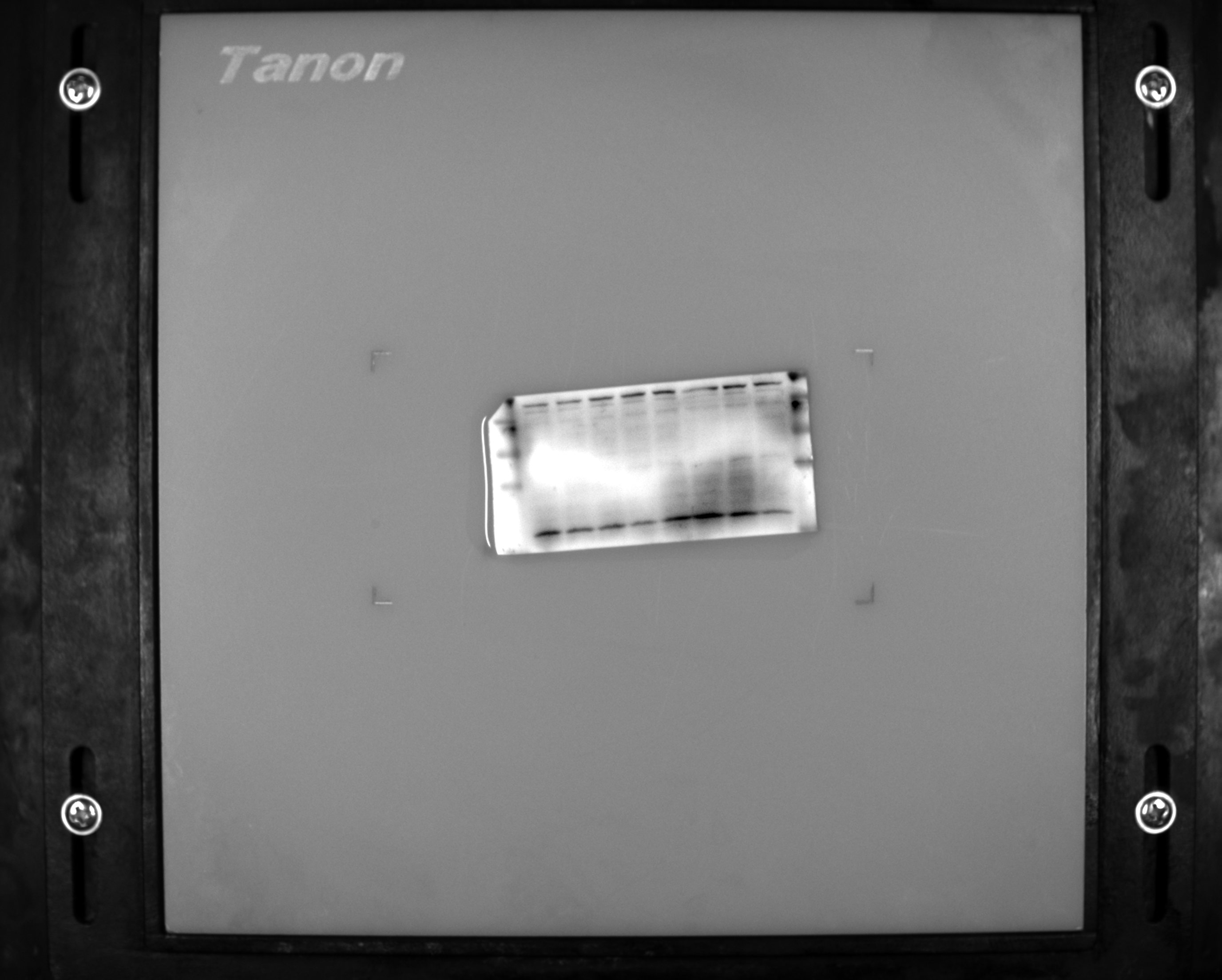

Supplement: Supplementary file 6 [file DataSheet_4.zip › Figure 5/Figure 5A/Caspase-1.Tif]

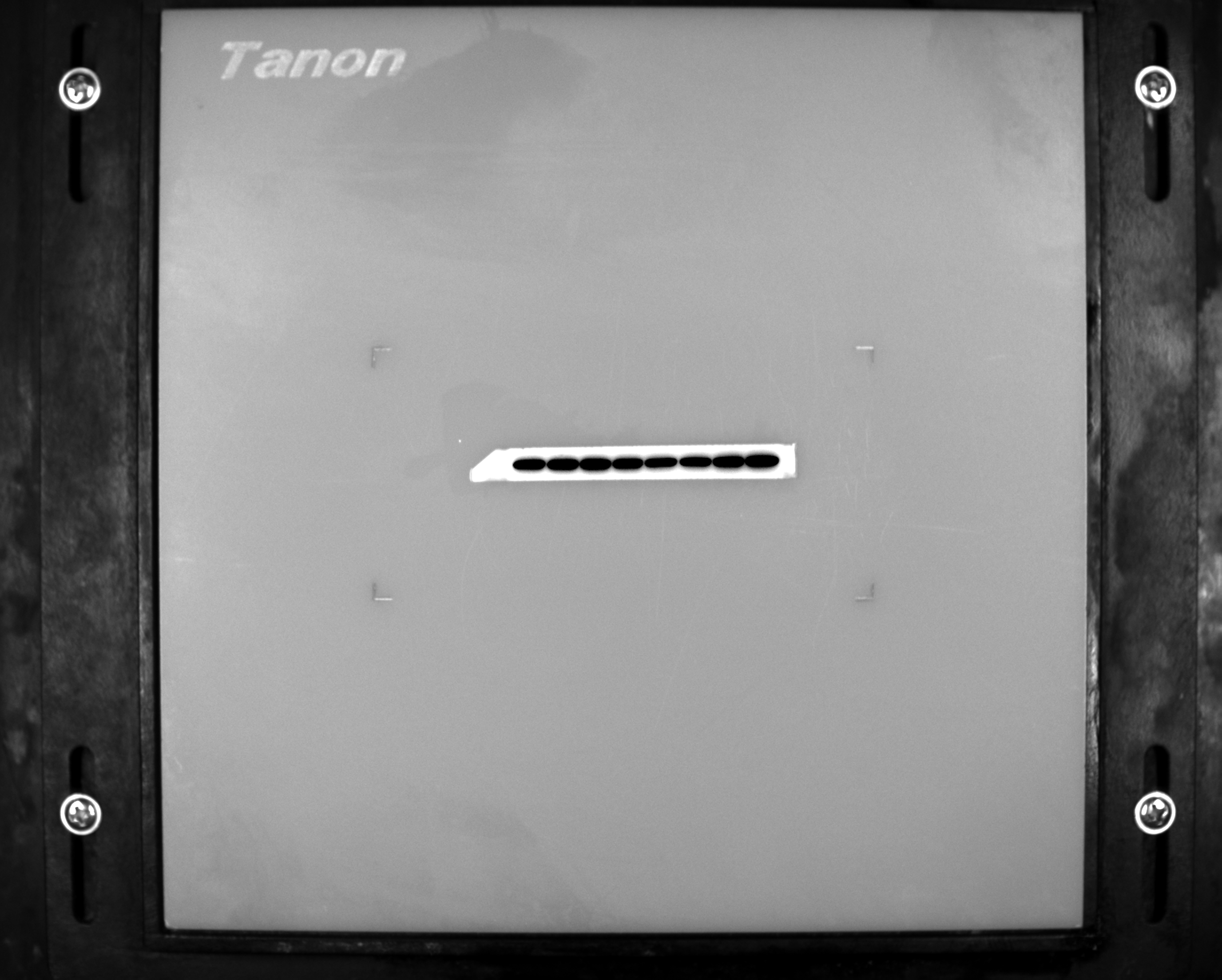

Supplement: Supplementary file 6 [file DataSheet_4.zip › Figure 5/Figure 5A/GAPDH.Tif]

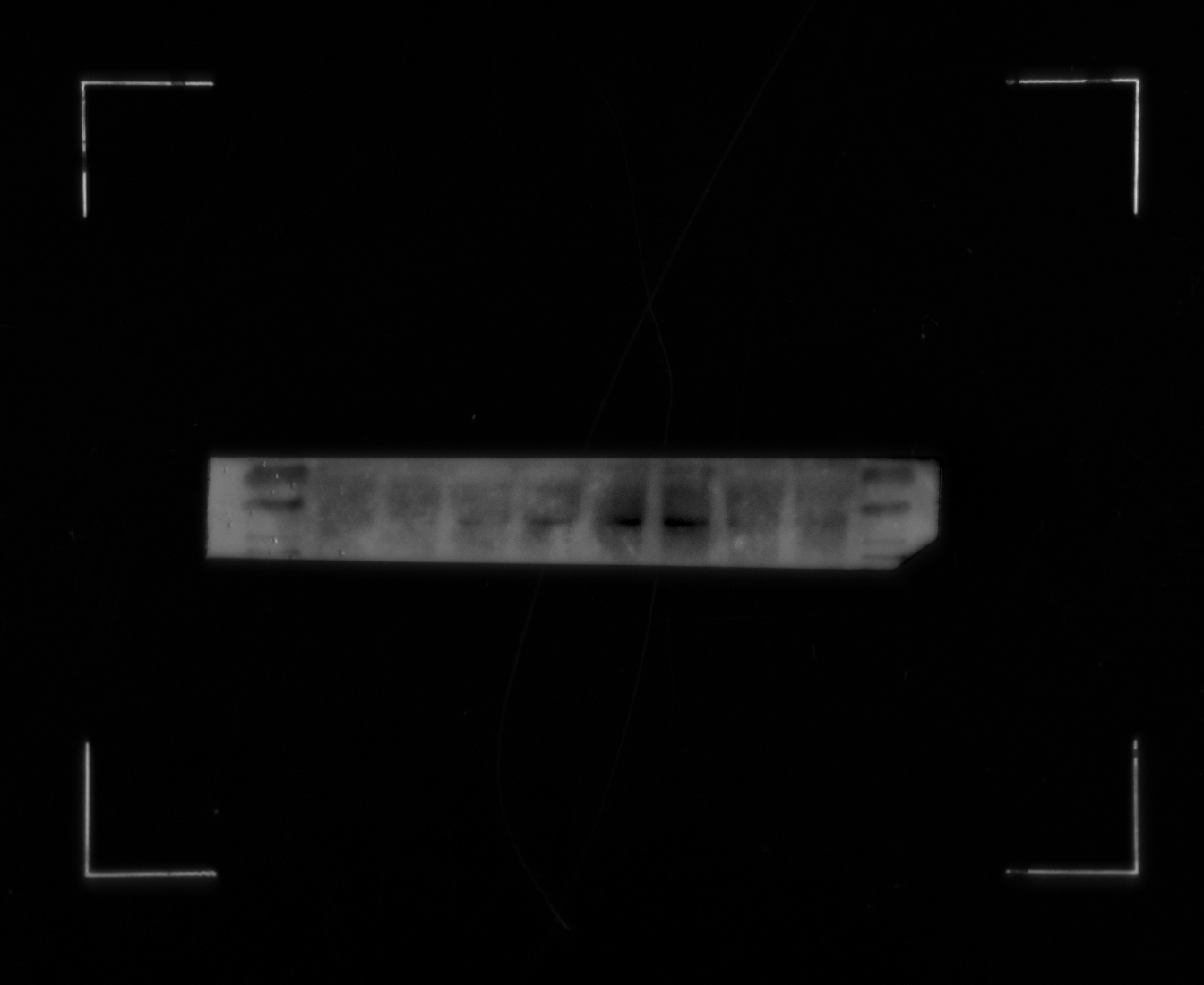

Supplement: Supplementary file 6 [file DataSheet_4.zip › Figure 5/Figure 5A/NLRP3.tif]

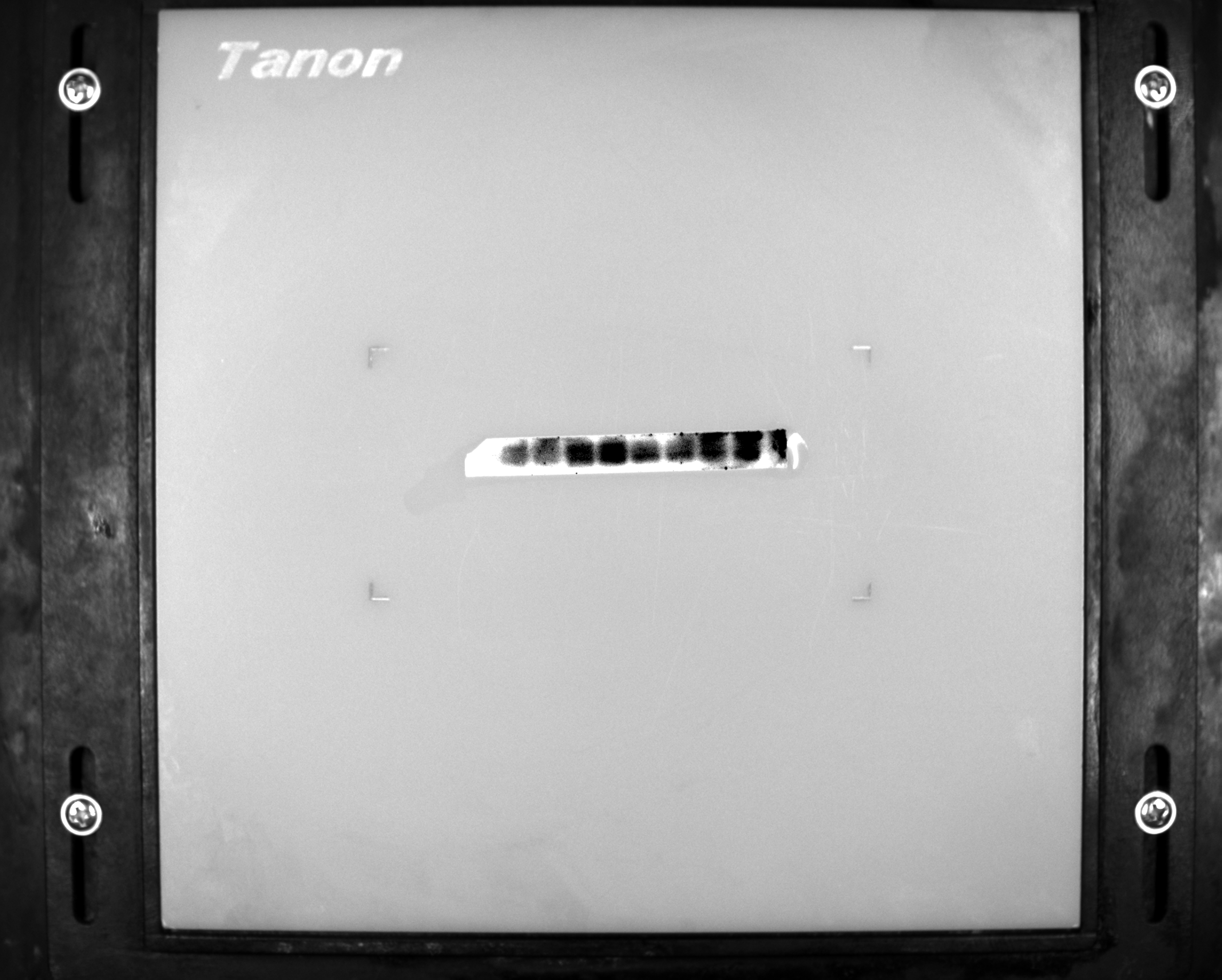

Supplement: Supplementary file 6 [file DataSheet_4.zip › Figure 5/Figure 5F/BCL-2.Tif]

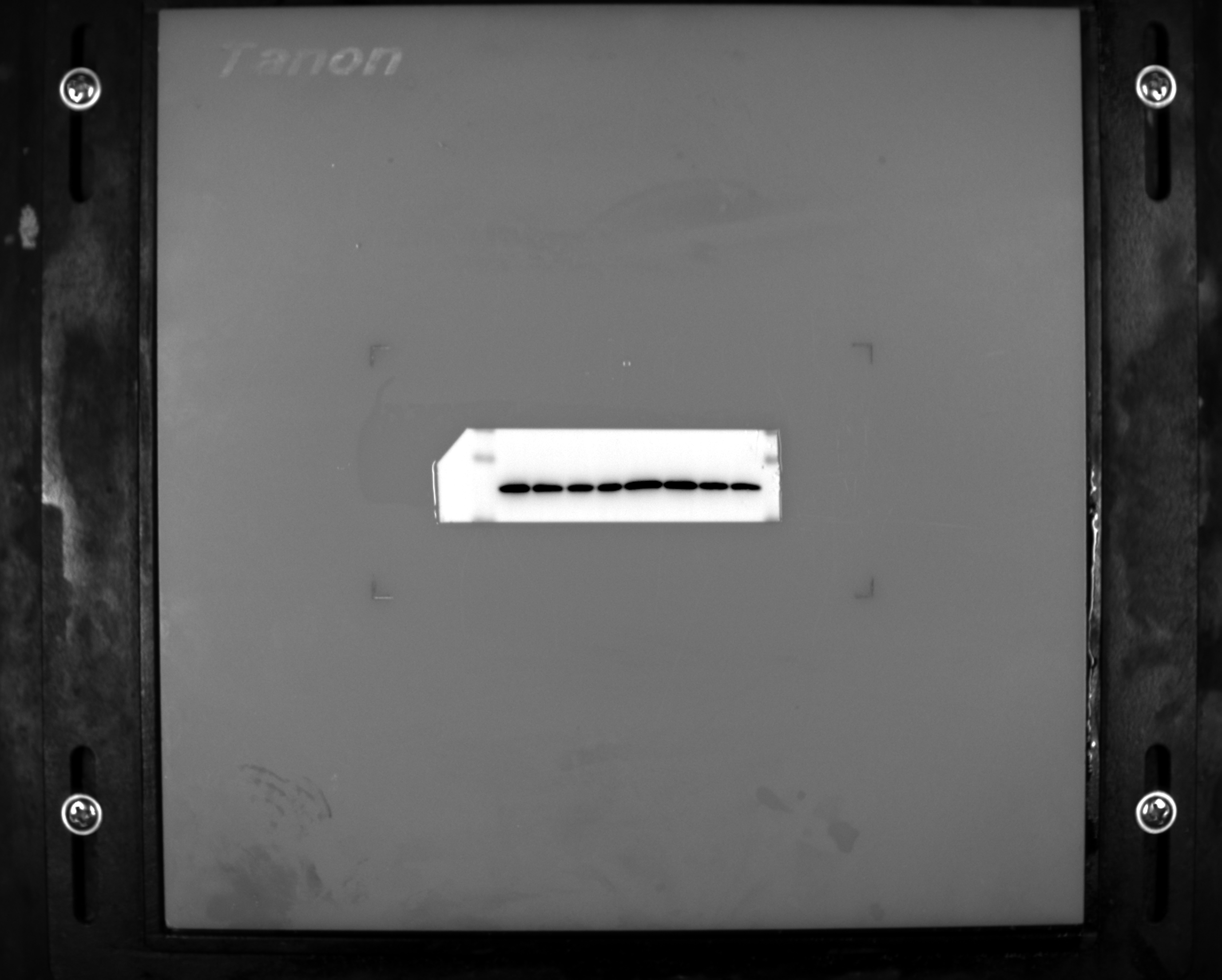

Supplement: Supplementary file 6 [file DataSheet_4.zip › Figure 5/Figure 5F/Bax.Tif]

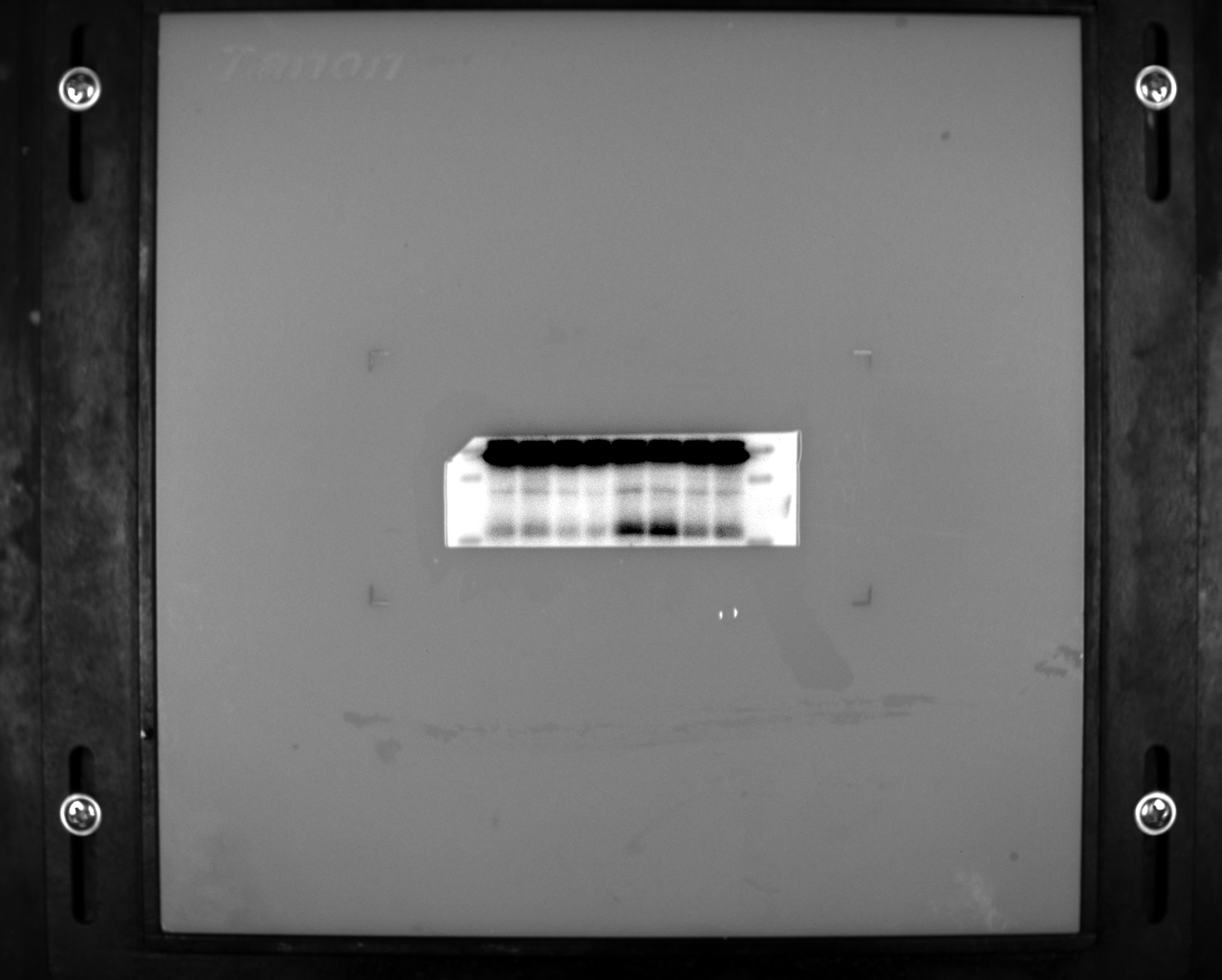

Supplement: Supplementary file 6 [file DataSheet_4.zip › Figure 5/Figure 5F/Caspase-3.Tif]

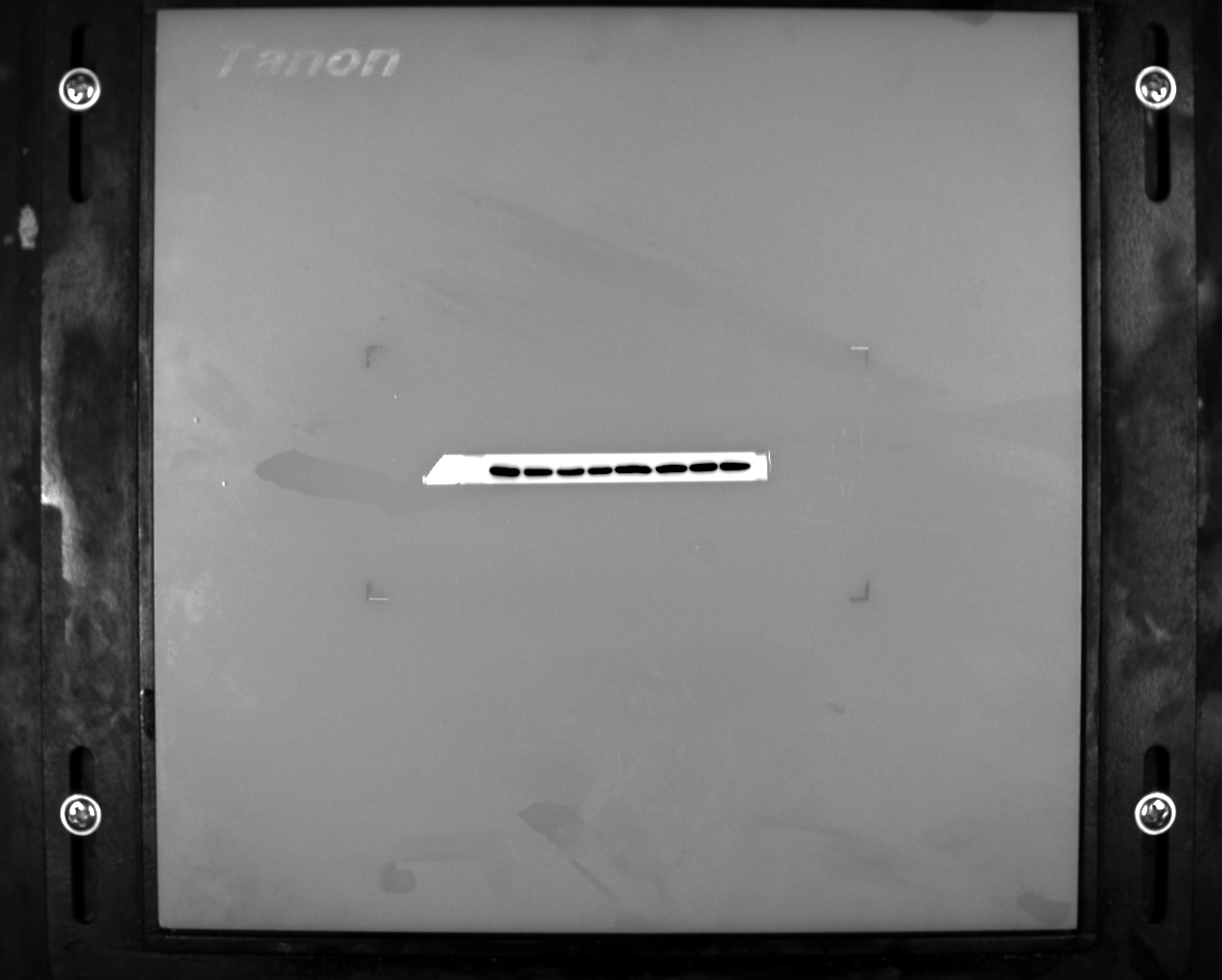

Supplement: Supplementary file 6 [file DataSheet_4.zip › Figure 5/Figure 5F/GAPDH.Tif]

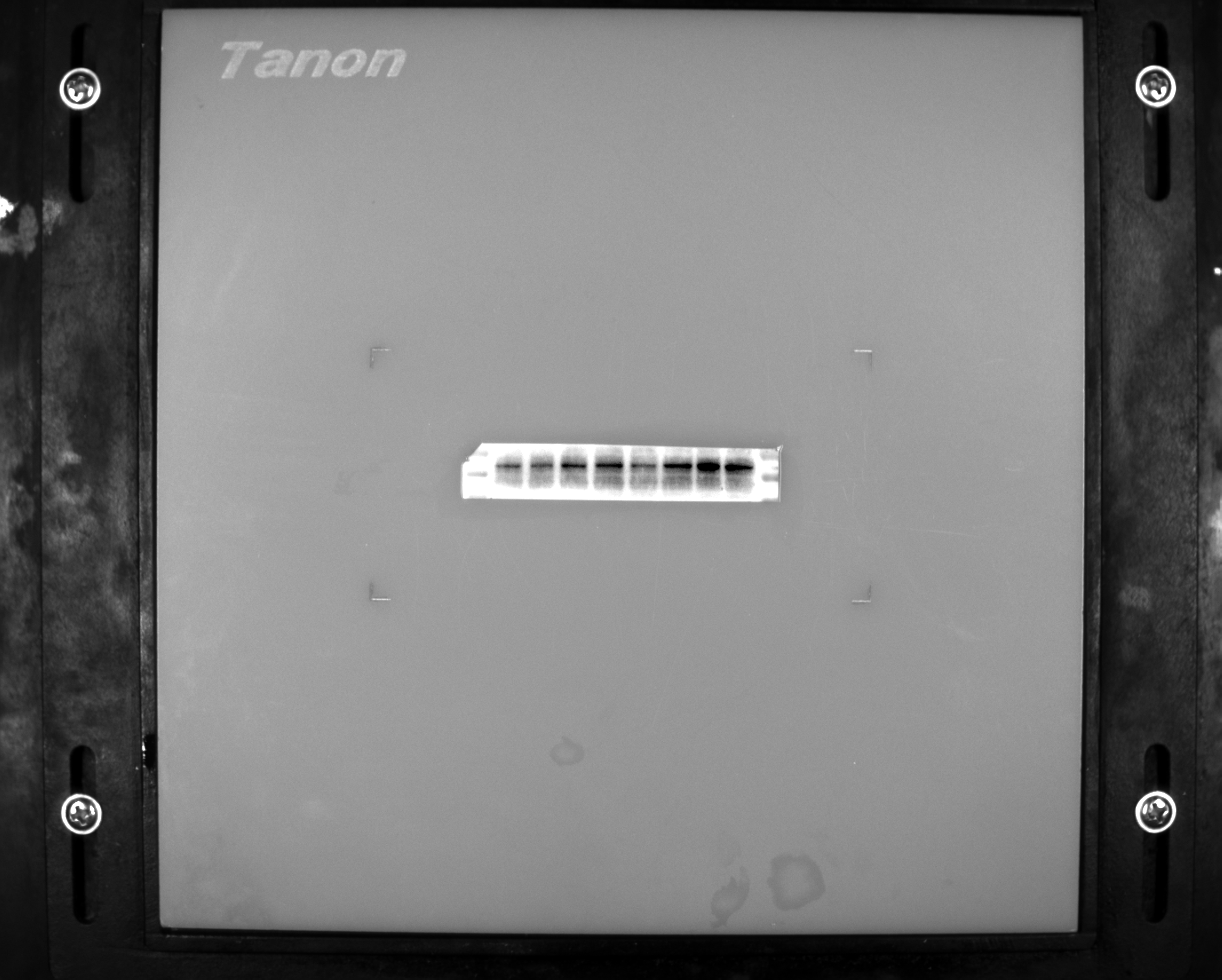

Supplement: Supplementary file 7 [file DataSheet_5.zip › Figure 6/ATG5.Tif]

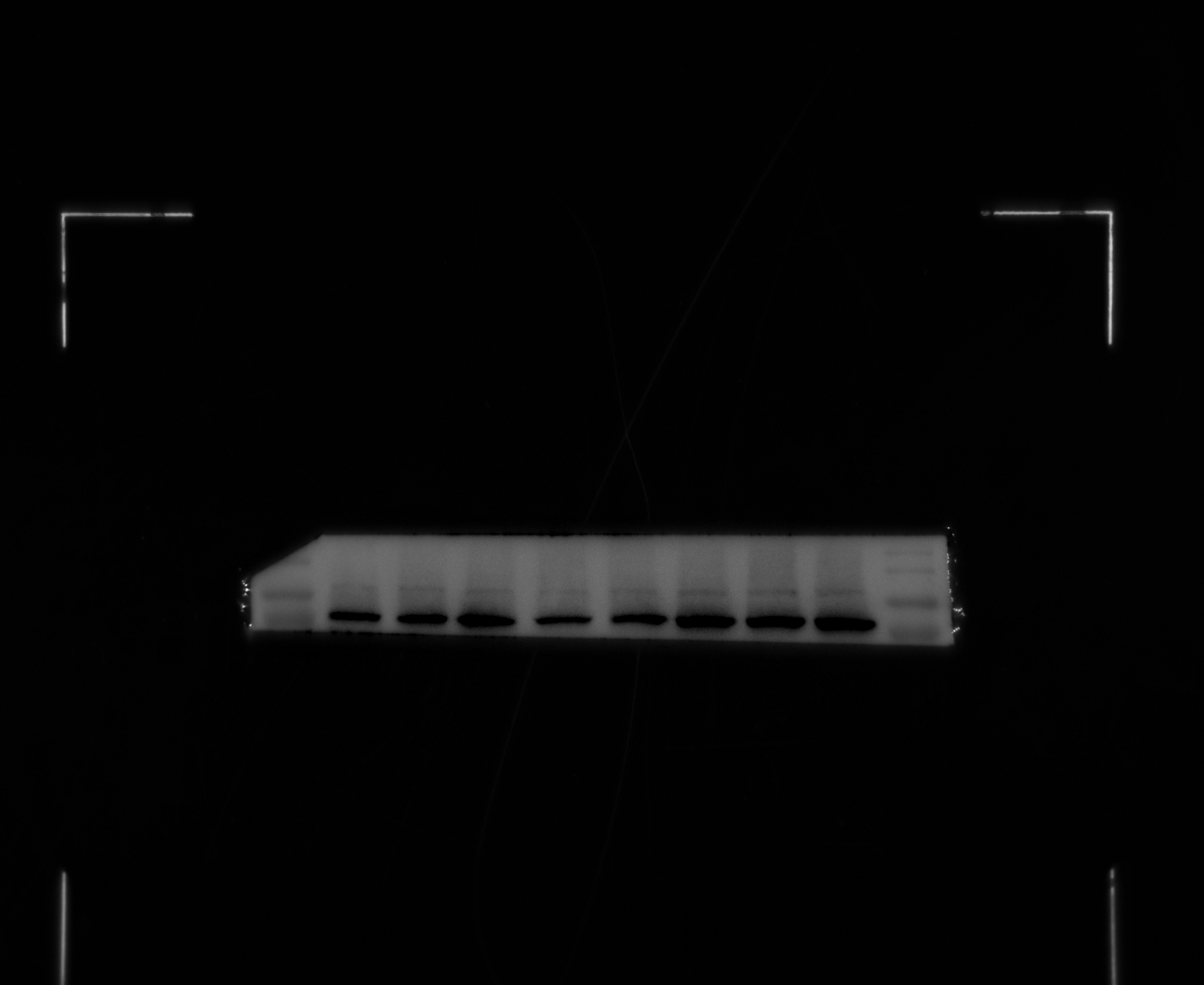

Supplement: Supplementary file 7 [file DataSheet_5.zip › Figure 6/ATG7.tif]

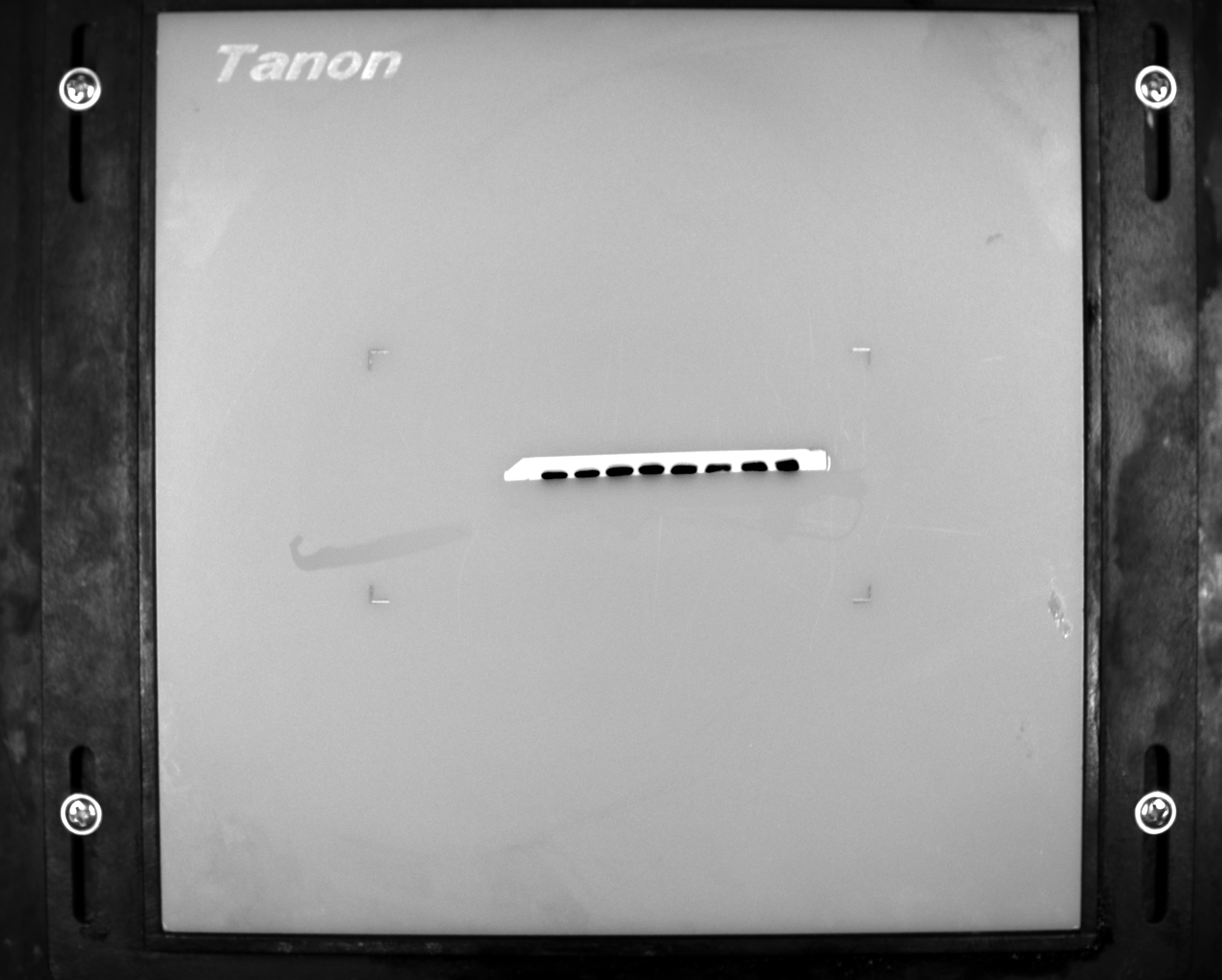

Supplement: Supplementary file 7 [file DataSheet_5.zip › Figure 6/Beclin-1.Tif]

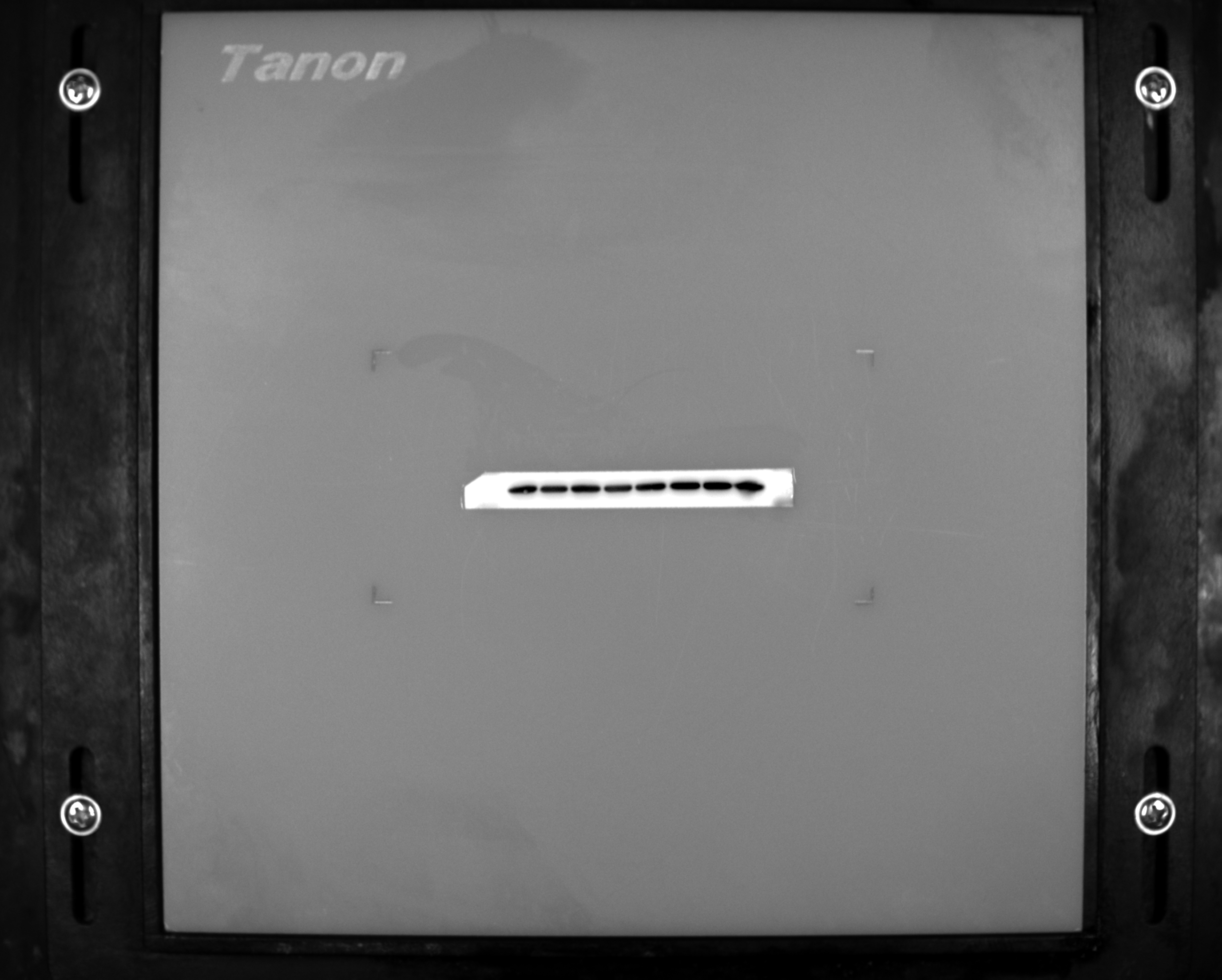

Supplement: Supplementary file 7 [file DataSheet_5.zip › Figure 6/GAPDH.Tif]

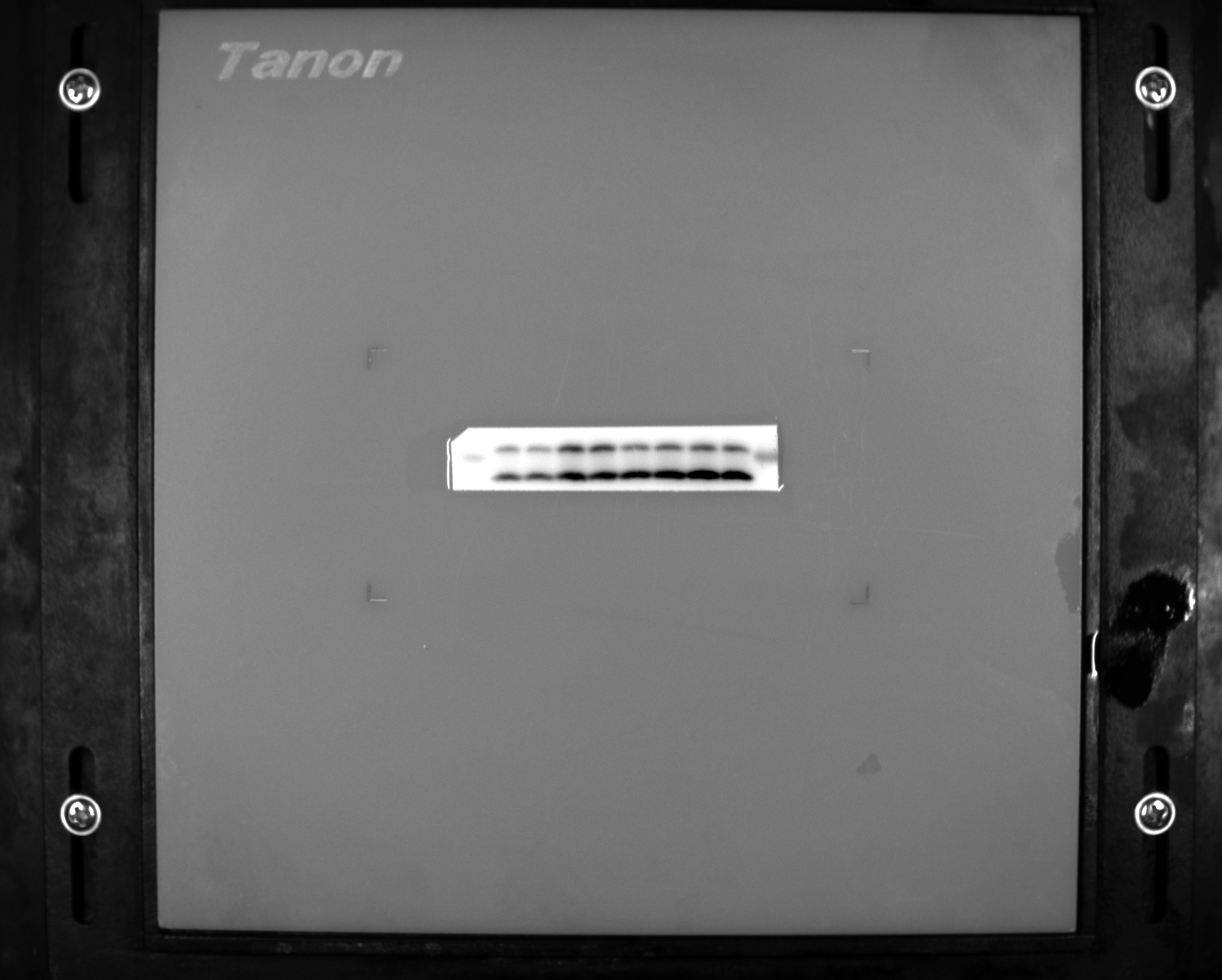

Supplement: Supplementary file 7 [file DataSheet_5.zip › Figure 6/LC3.Tif]

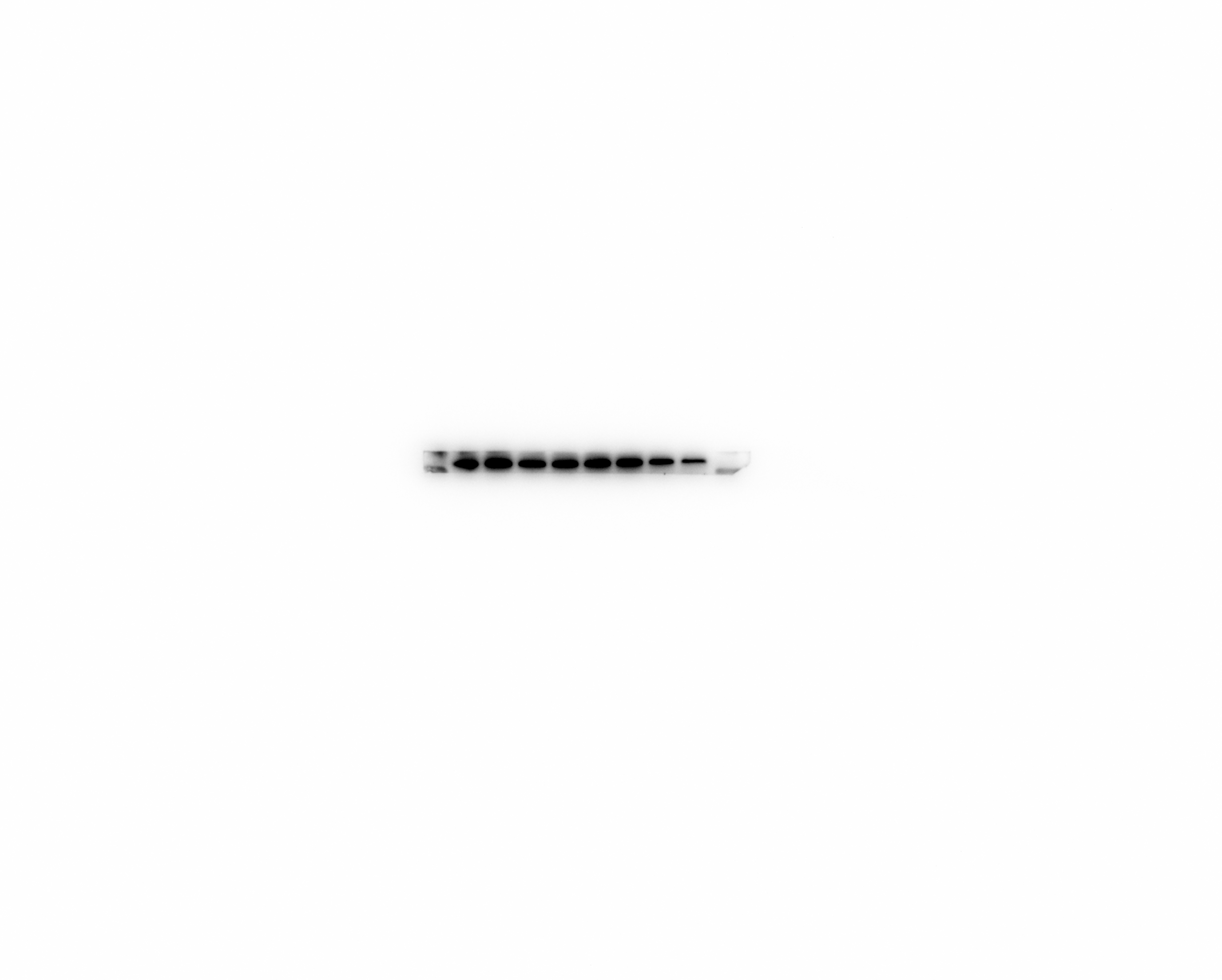

Supplement: Supplementary file 7 [file DataSheet_5.zip › Figure 6/P62 .Tif]

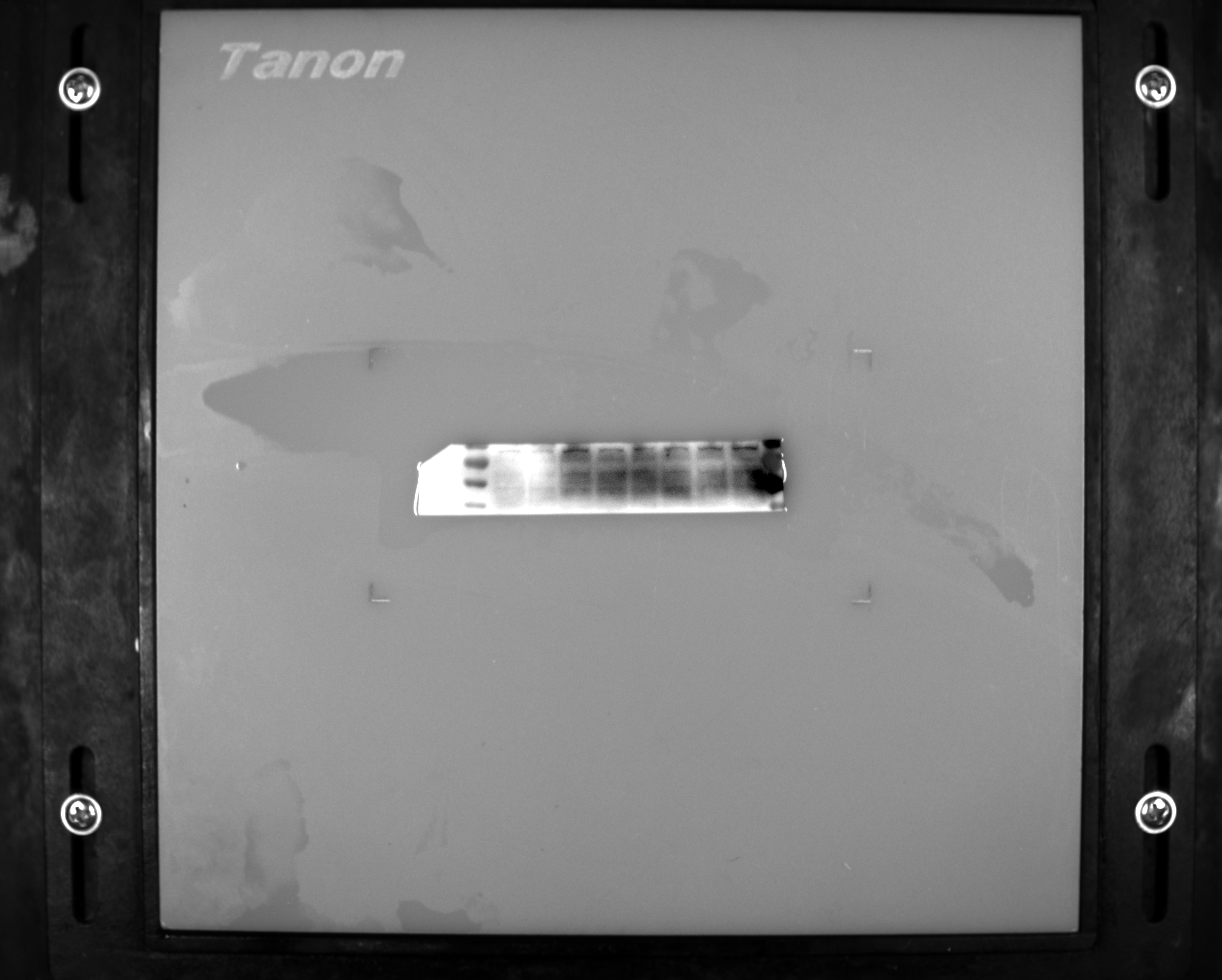

Supplement: Supplementary file 7 [file DataSheet_5.zip › Figure 6/PINK1.Tif]

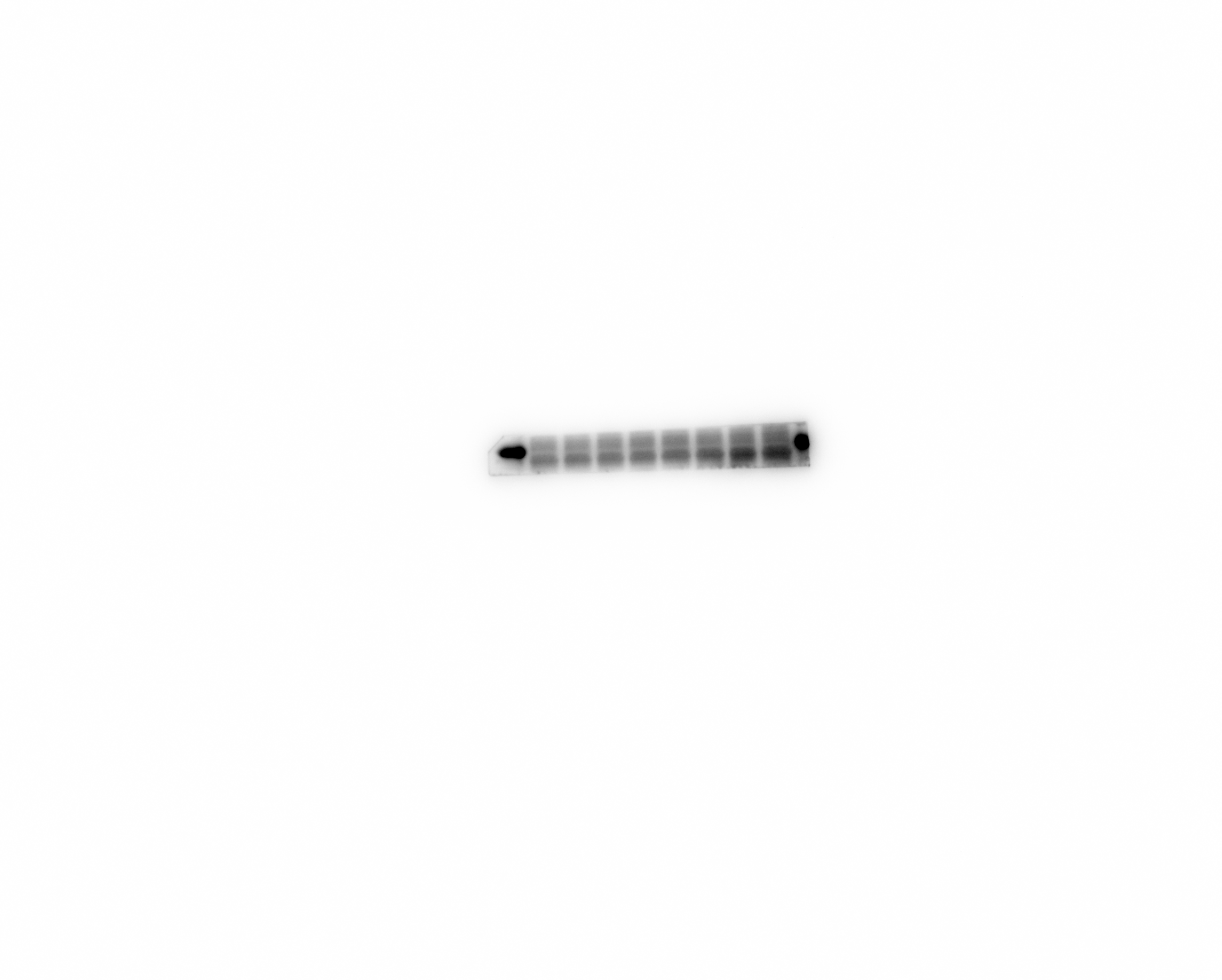

Supplement: Supplementary file 7 [file DataSheet_5.zip › Figure 6/Parkin.Tif]

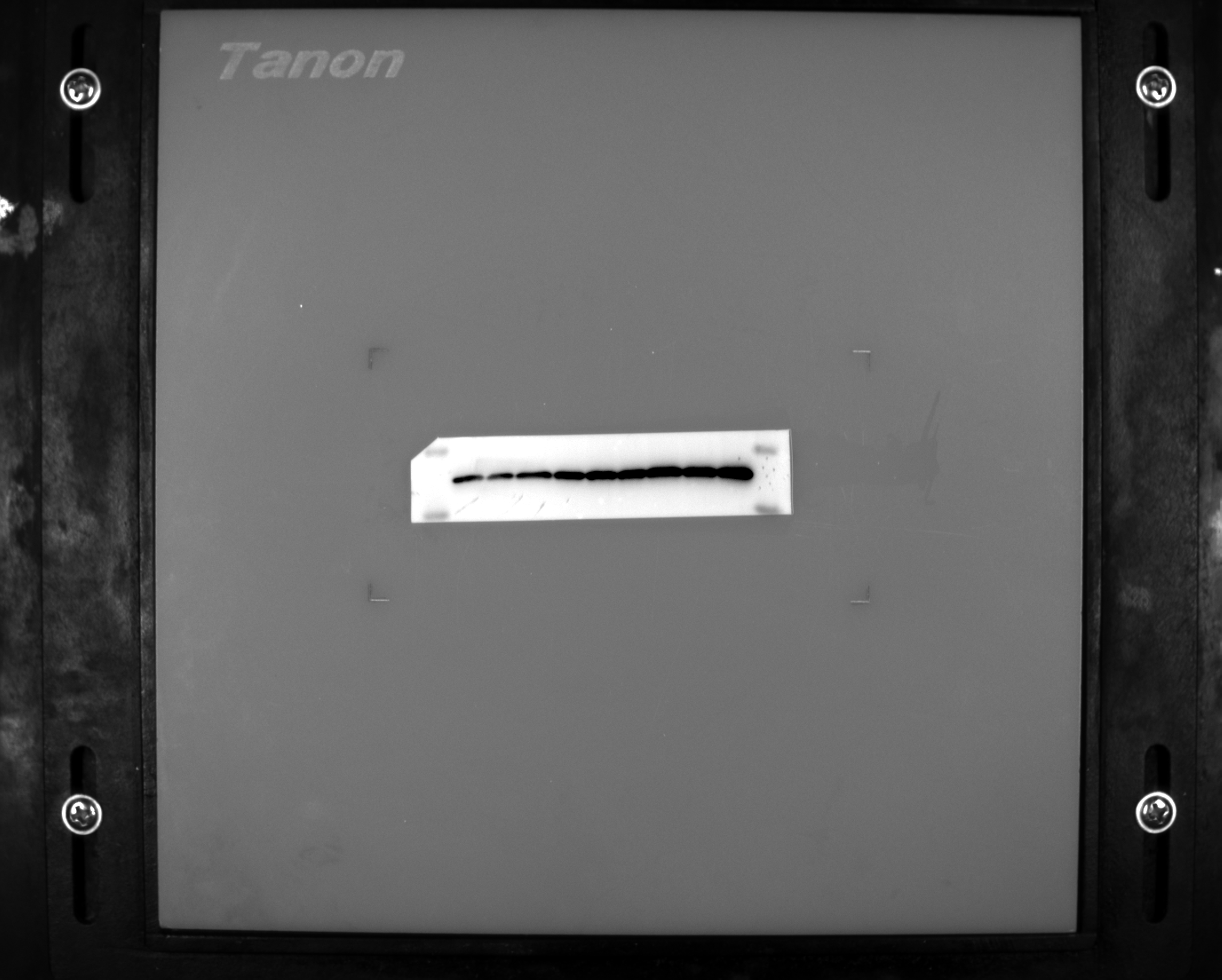

Supplement: Supplementary file 8 [file DataSheet_6.zip › Figure 7/Figure 7E/Bax.Tif]

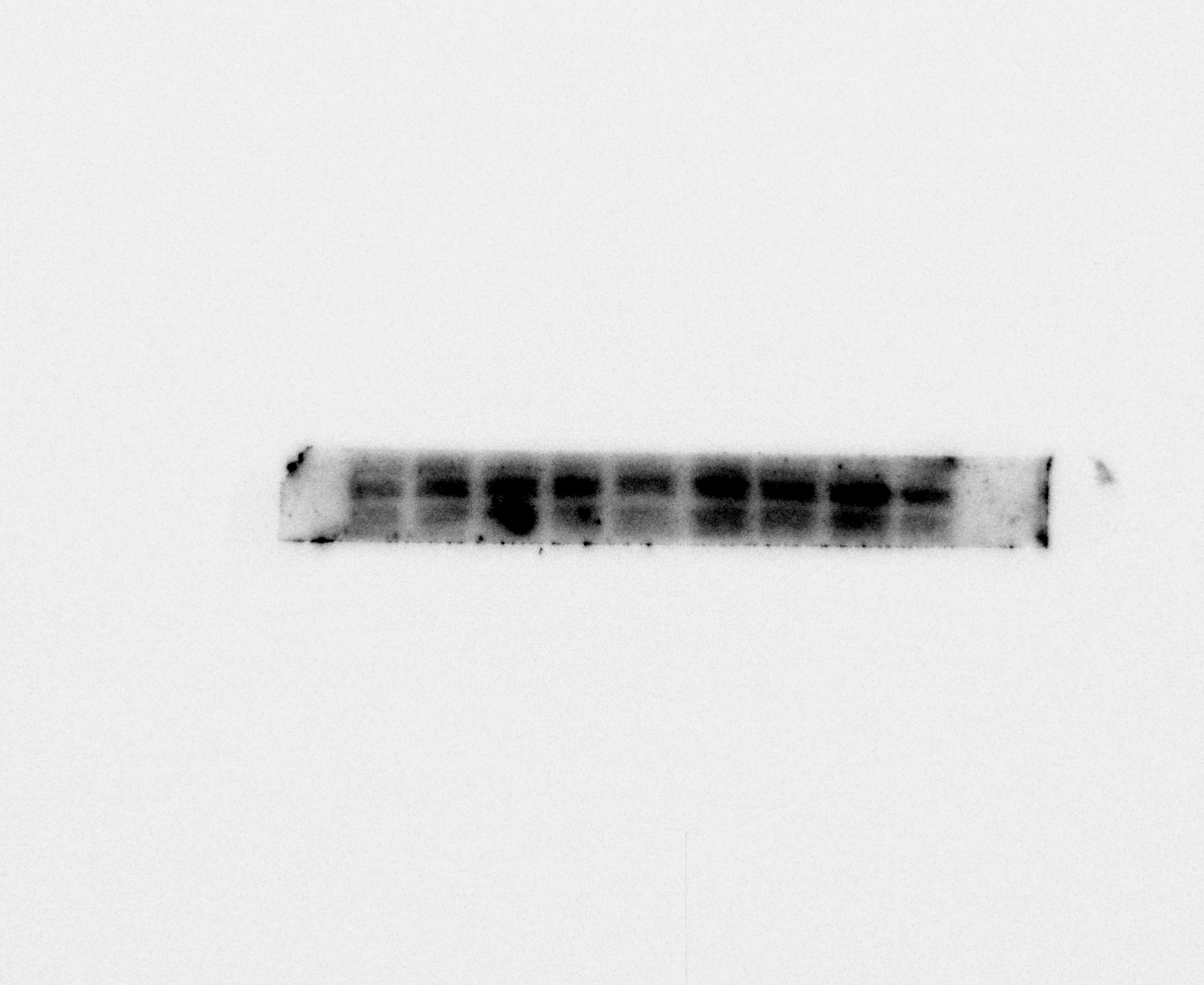

Supplement: Supplementary file 8 [file DataSheet_6.zip › Figure 7/Figure 7E/Bcl-2.tif]

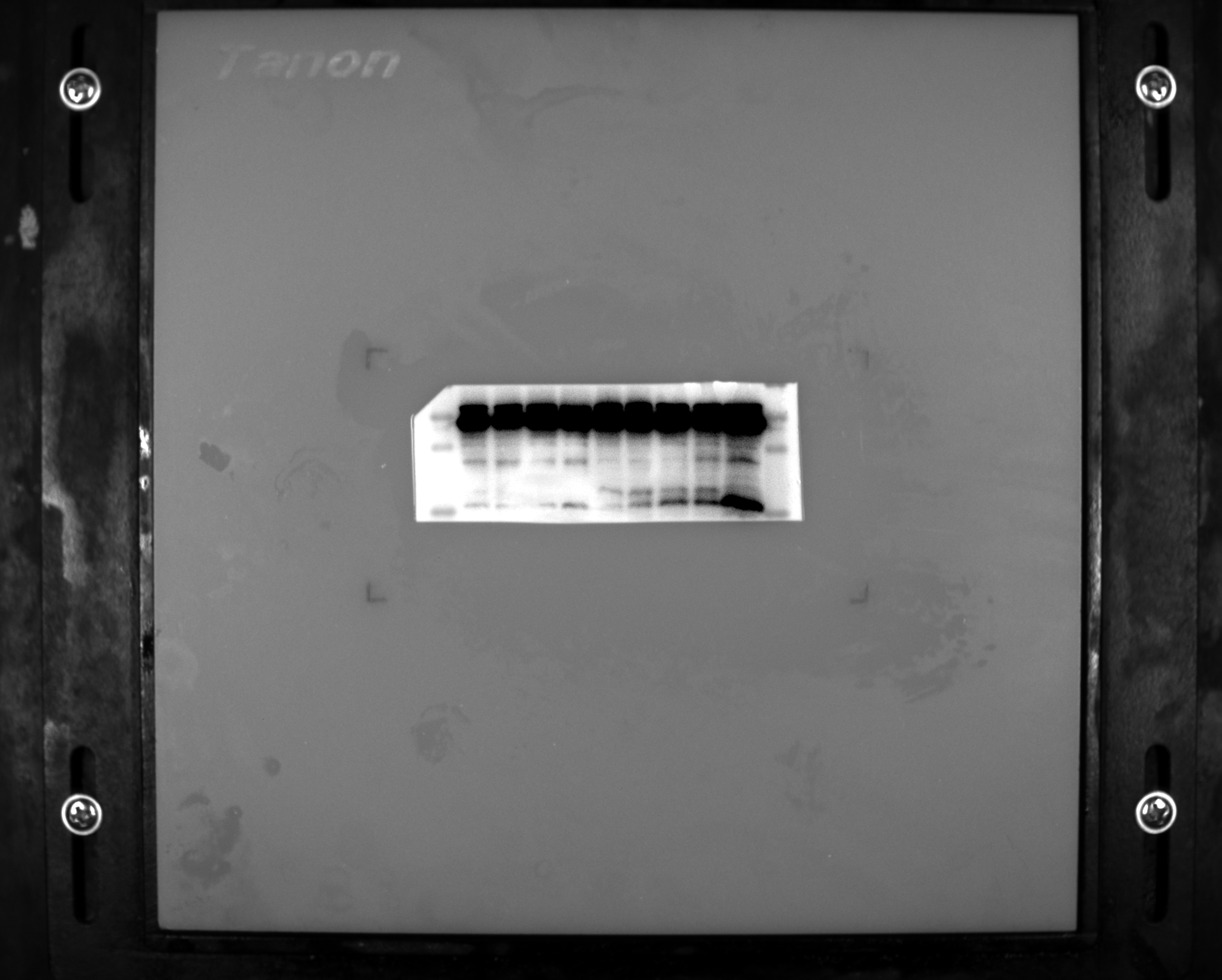

Supplement: Supplementary file 8 [file DataSheet_6.zip › Figure 7/Figure 7E/Caspase-3.Tif]

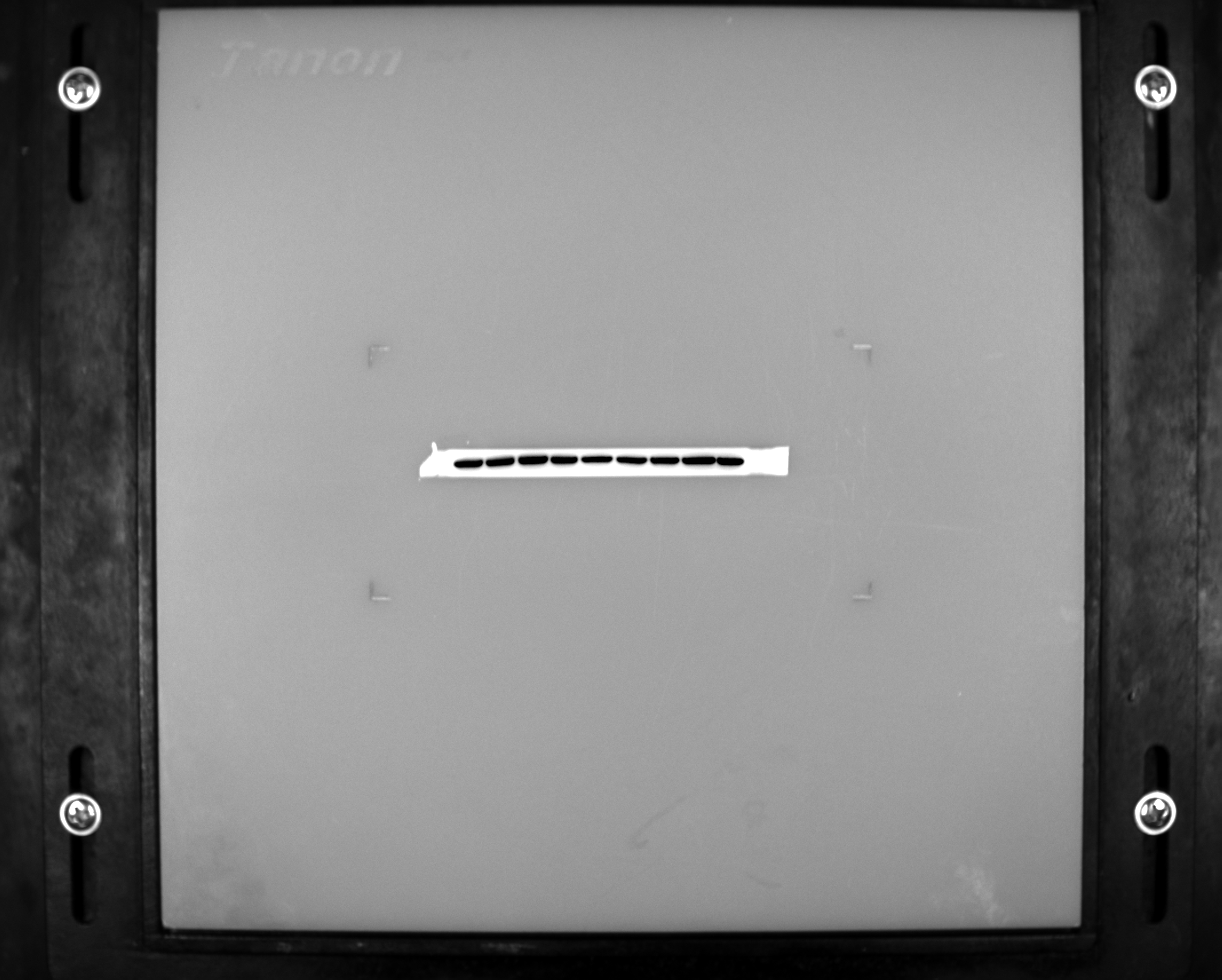

Supplement: Supplementary file 8 [file DataSheet_6.zip › Figure 7/Figure 7E/GAPDH.Tif]

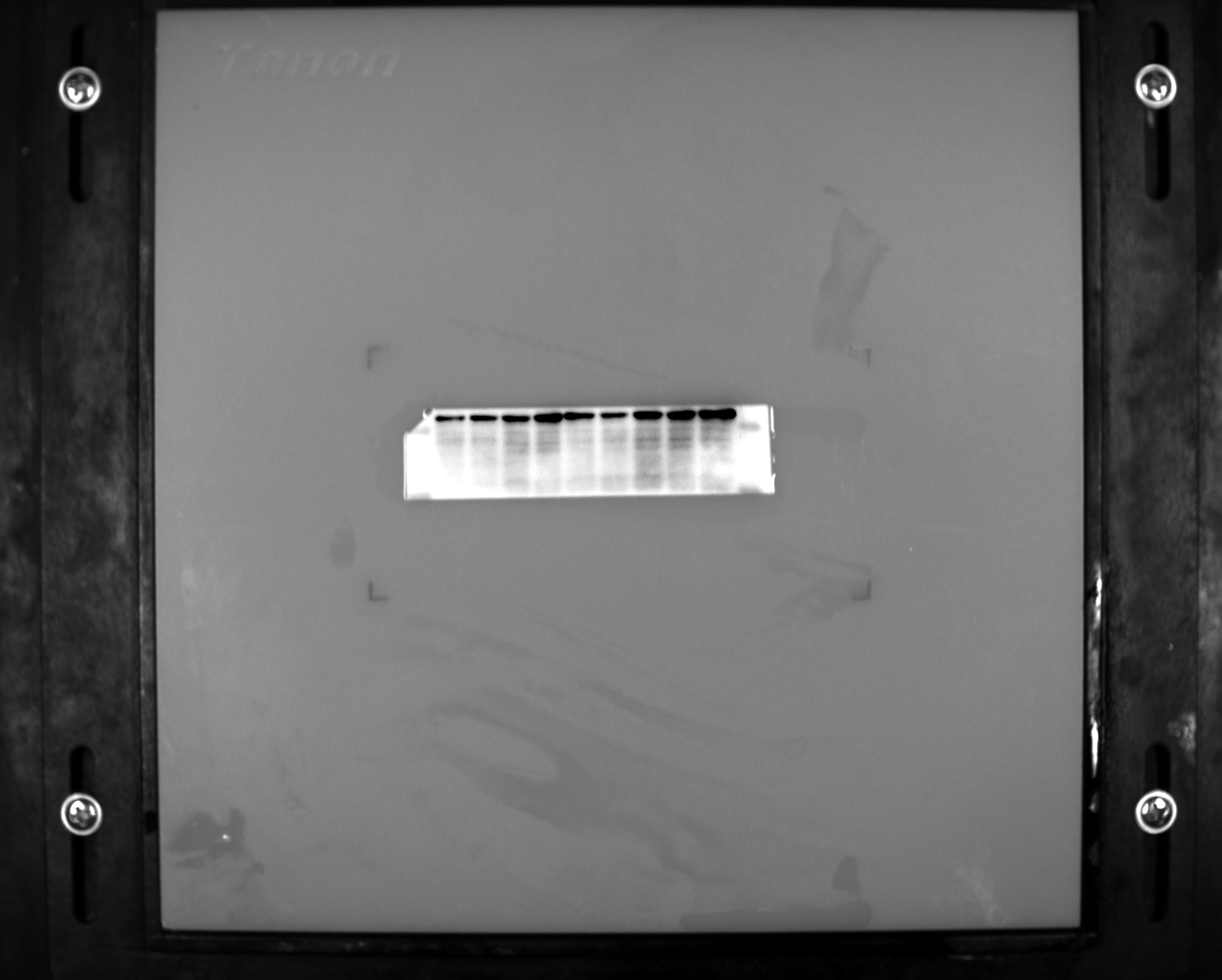

Supplement: Supplementary file 8 [file DataSheet_6.zip › Figure 7/Figure 7F/ASC.Tif]

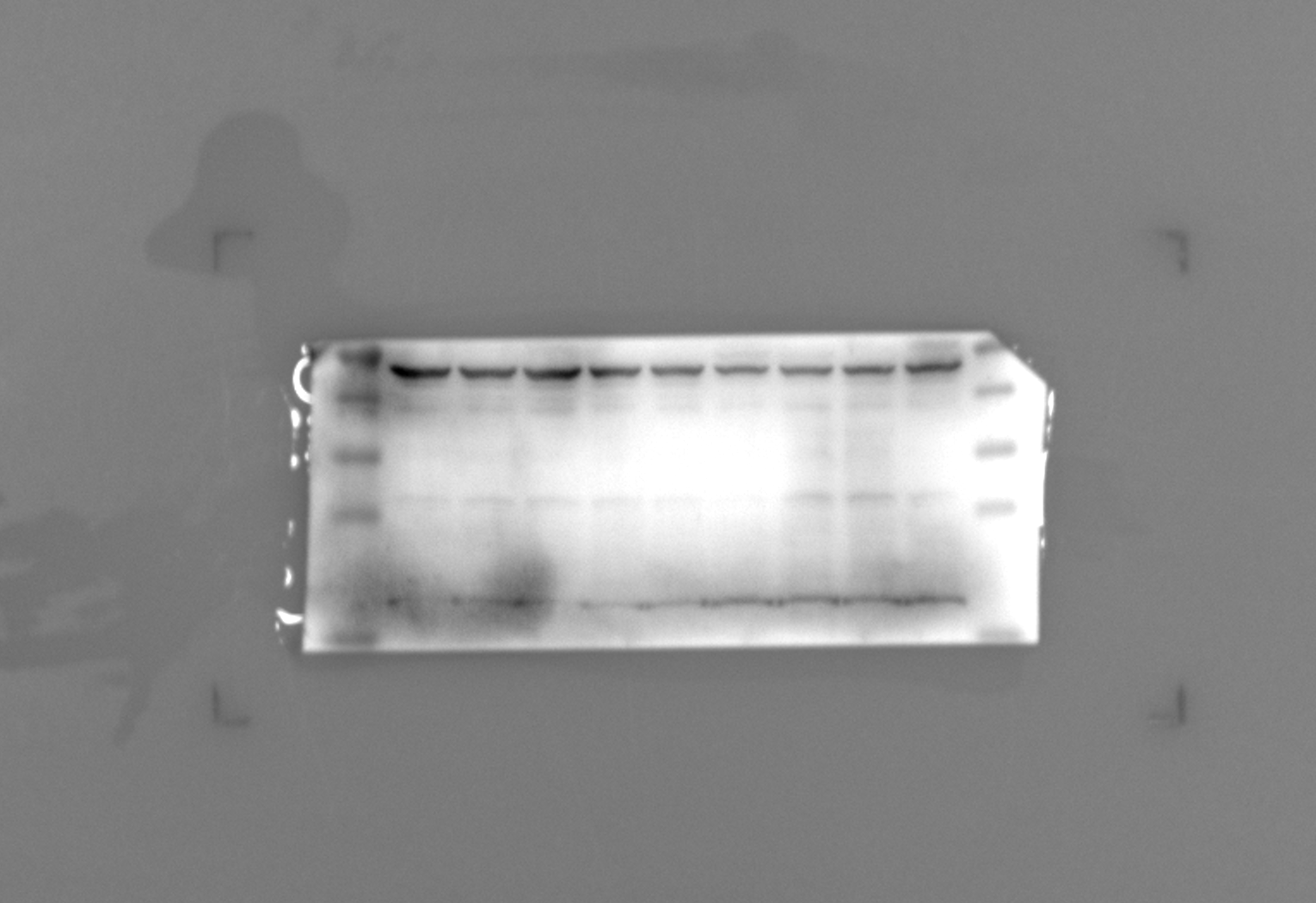

Supplement: Supplementary file 8 [file DataSheet_6.zip › Figure 7/Figure 7F/Caspase-1.Tif]

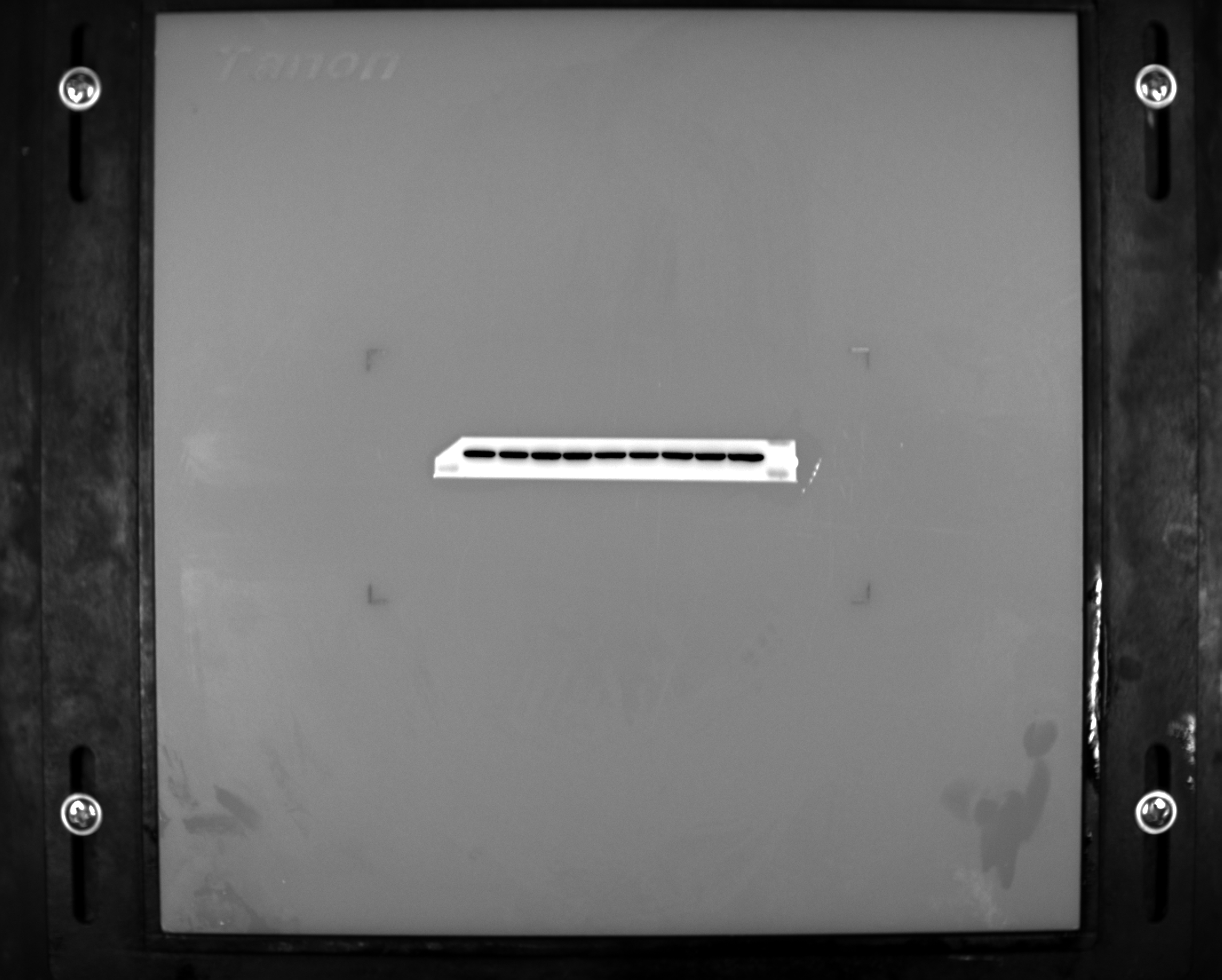

Supplement: Supplementary file 8 [file DataSheet_6.zip › Figure 7/Figure 7F/GAPDH.Tif]

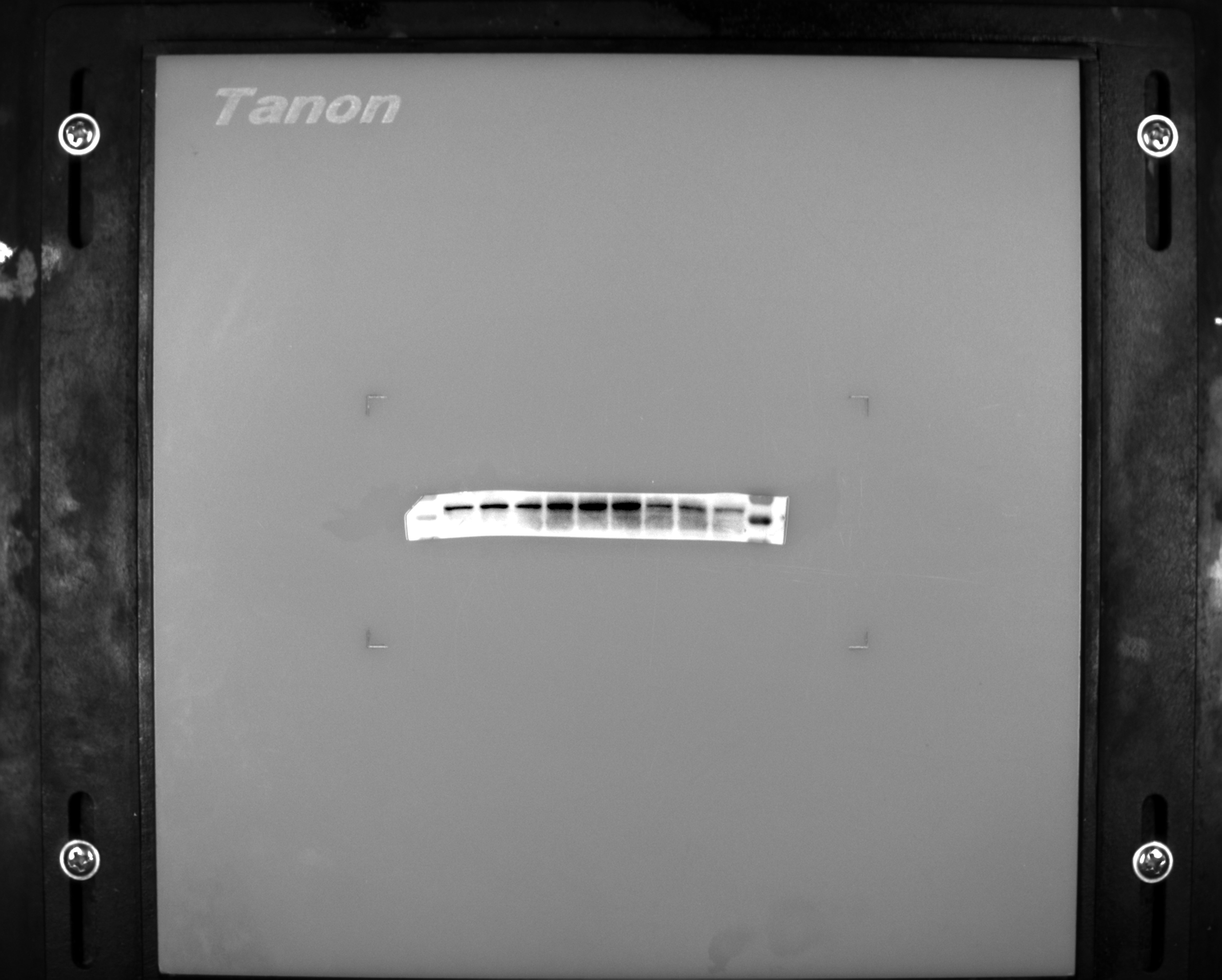

Supplement: Supplementary file 9 [file DataSheet_7.zip › Figure 8/ATG5.Tif]

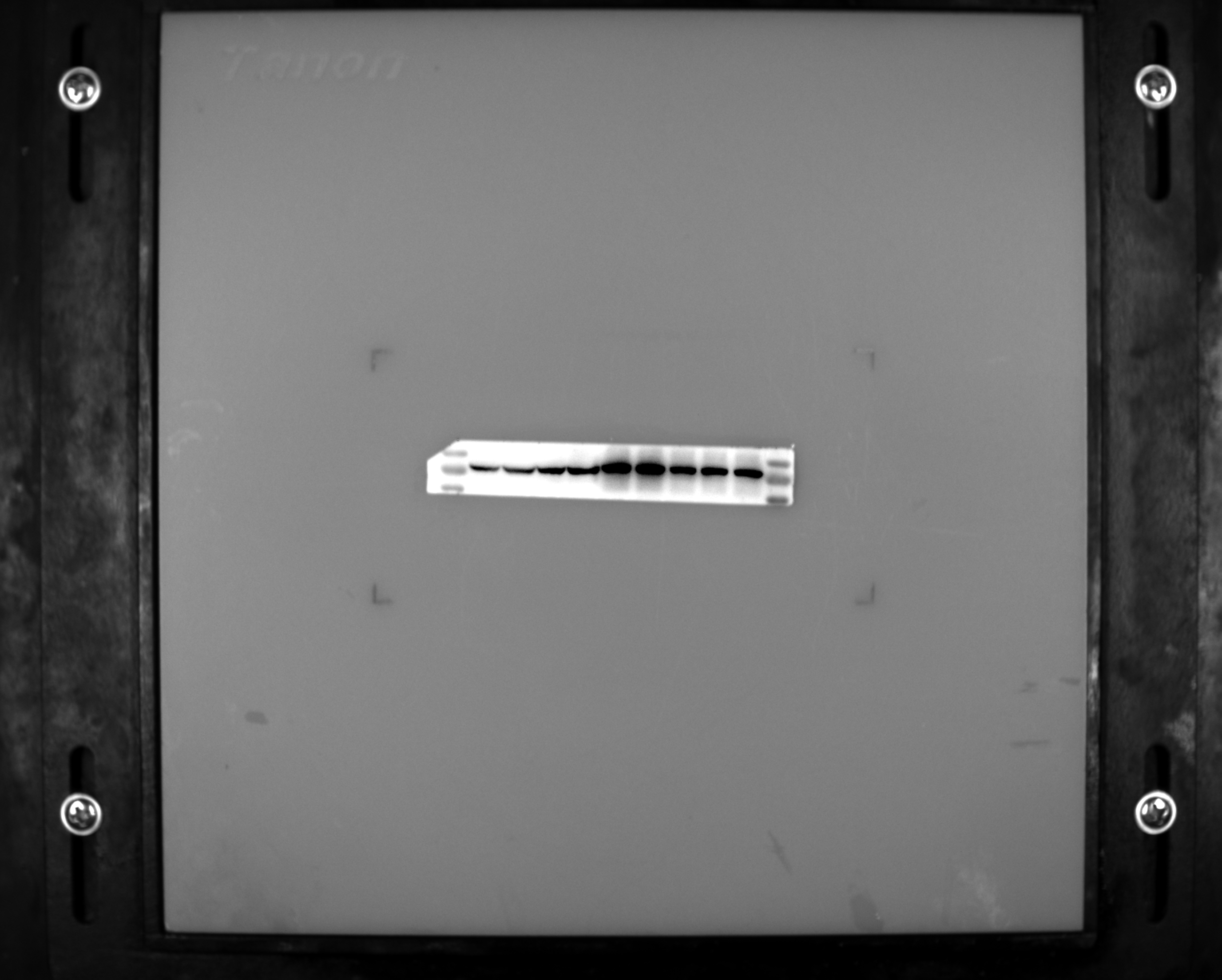

Supplement: Supplementary file 9 [file DataSheet_7.zip › Figure 8/ATG7.Tif]

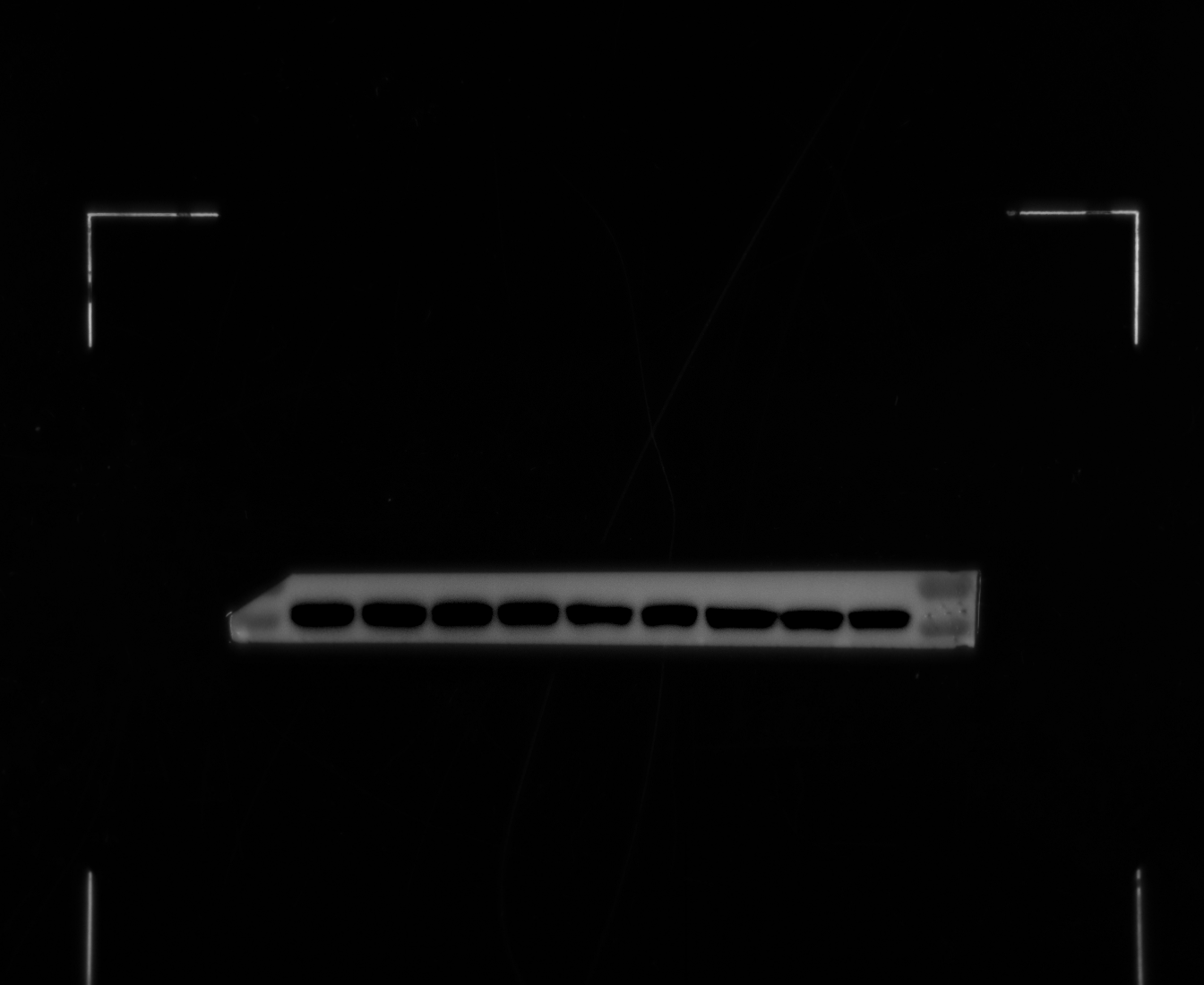

Supplement: Supplementary file 9 [file DataSheet_7.zip › Figure 8/Beclin-1.tif]

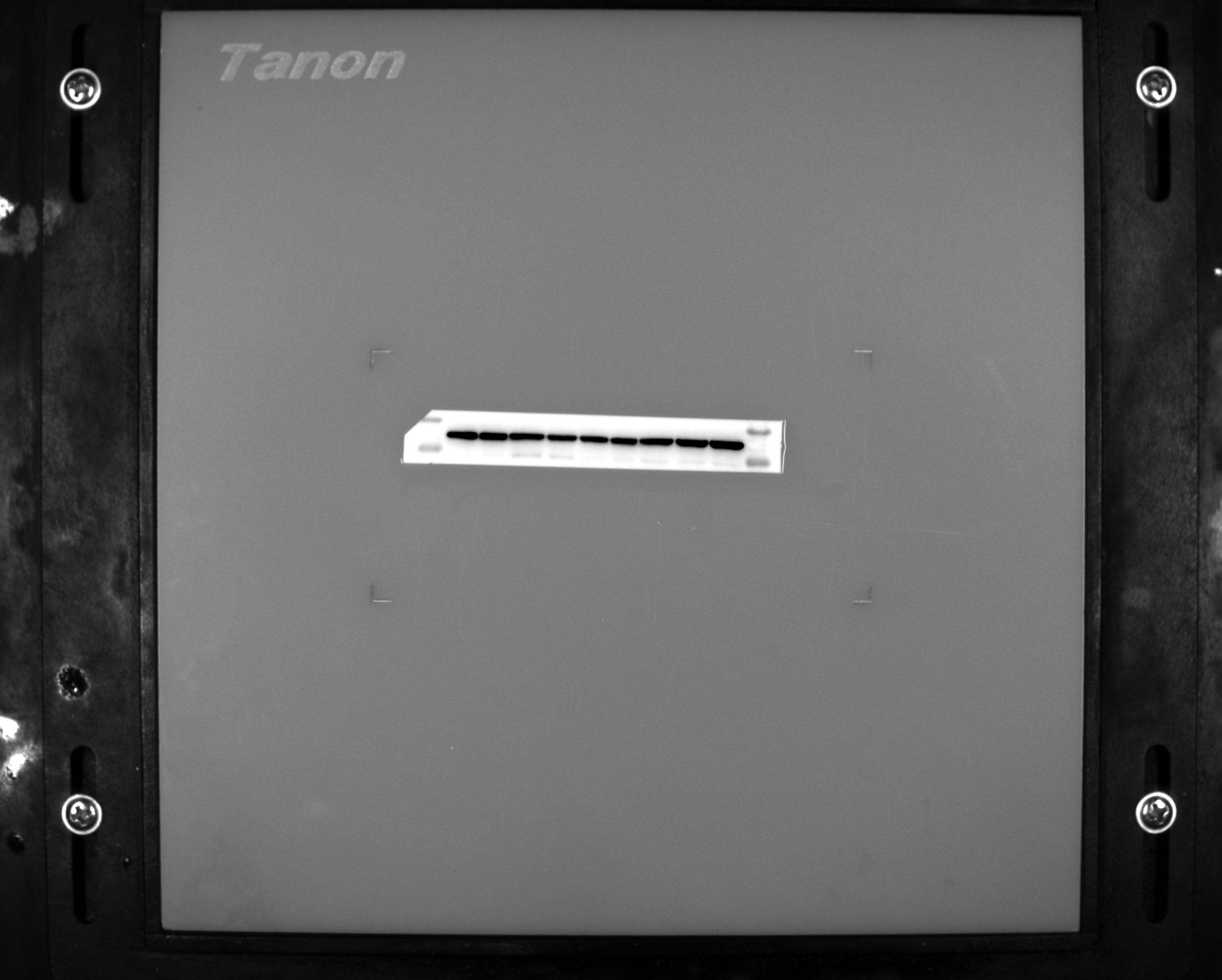

Supplement: Supplementary file 9 [file DataSheet_7.zip › Figure 8/GAPDH.Tif]

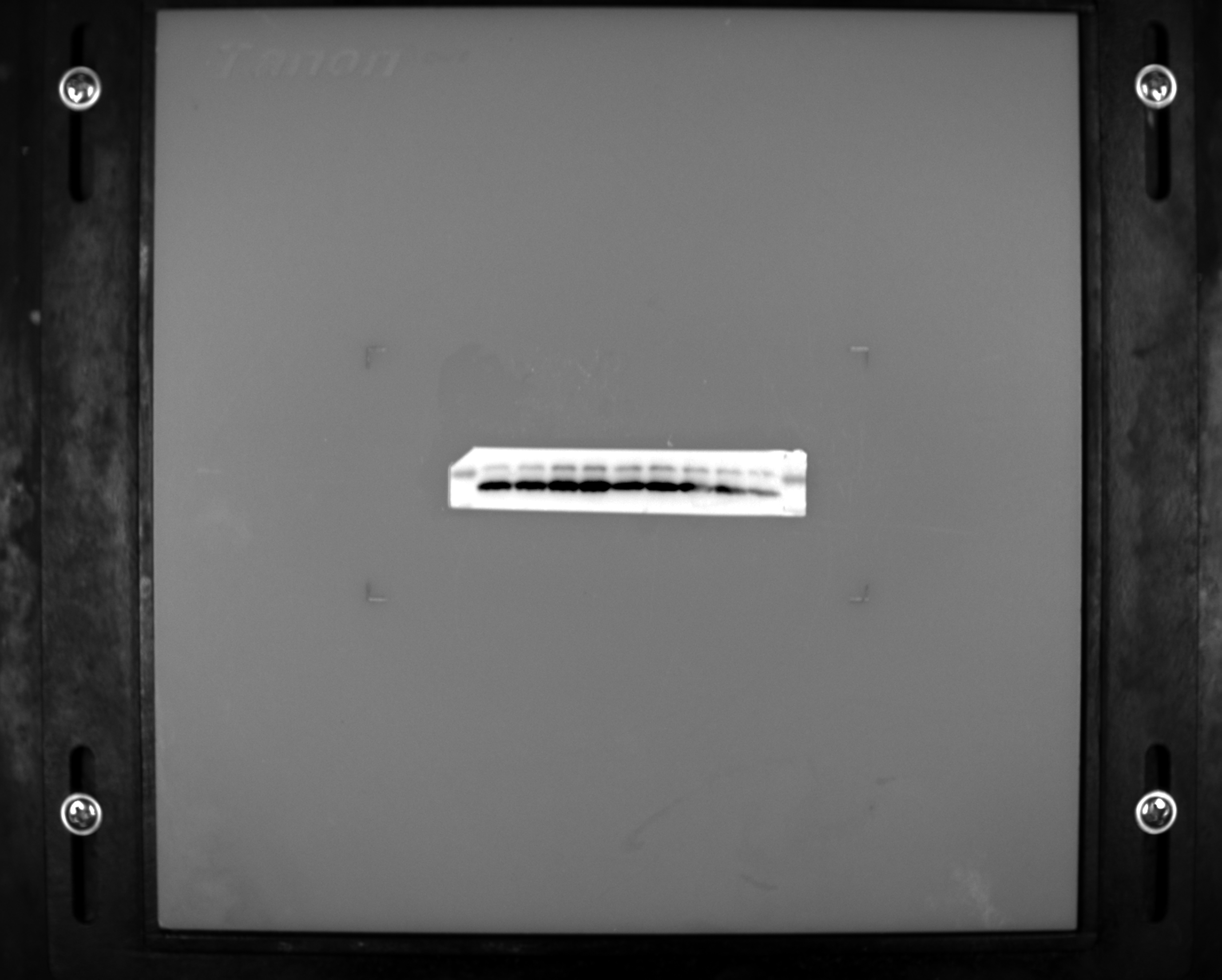

Supplement: Supplementary file 9 [file DataSheet_7.zip › Figure 8/LC3.Tif]

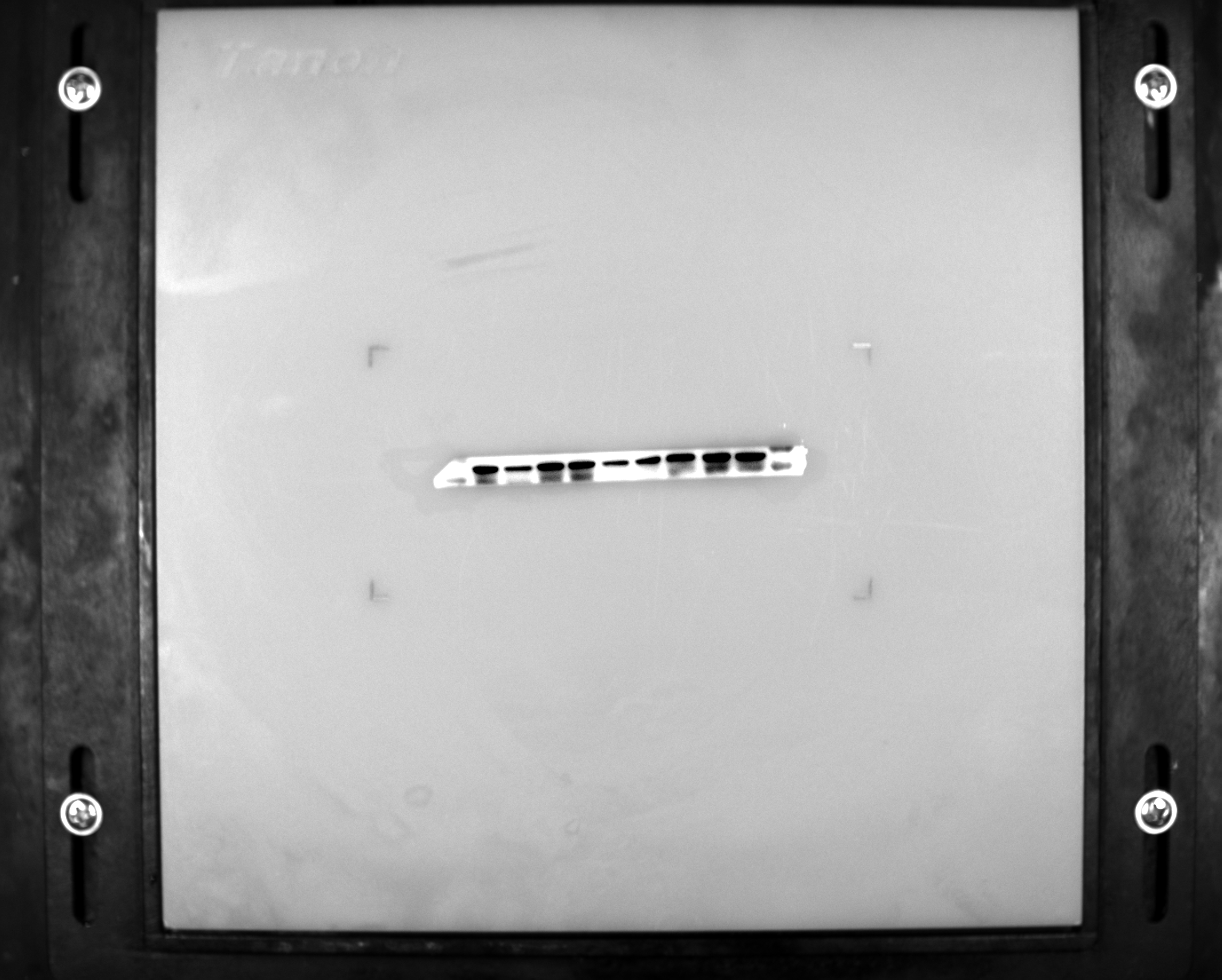

Supplement: Supplementary file 9 [file DataSheet_7.zip › Figure 8/P62.Tif]

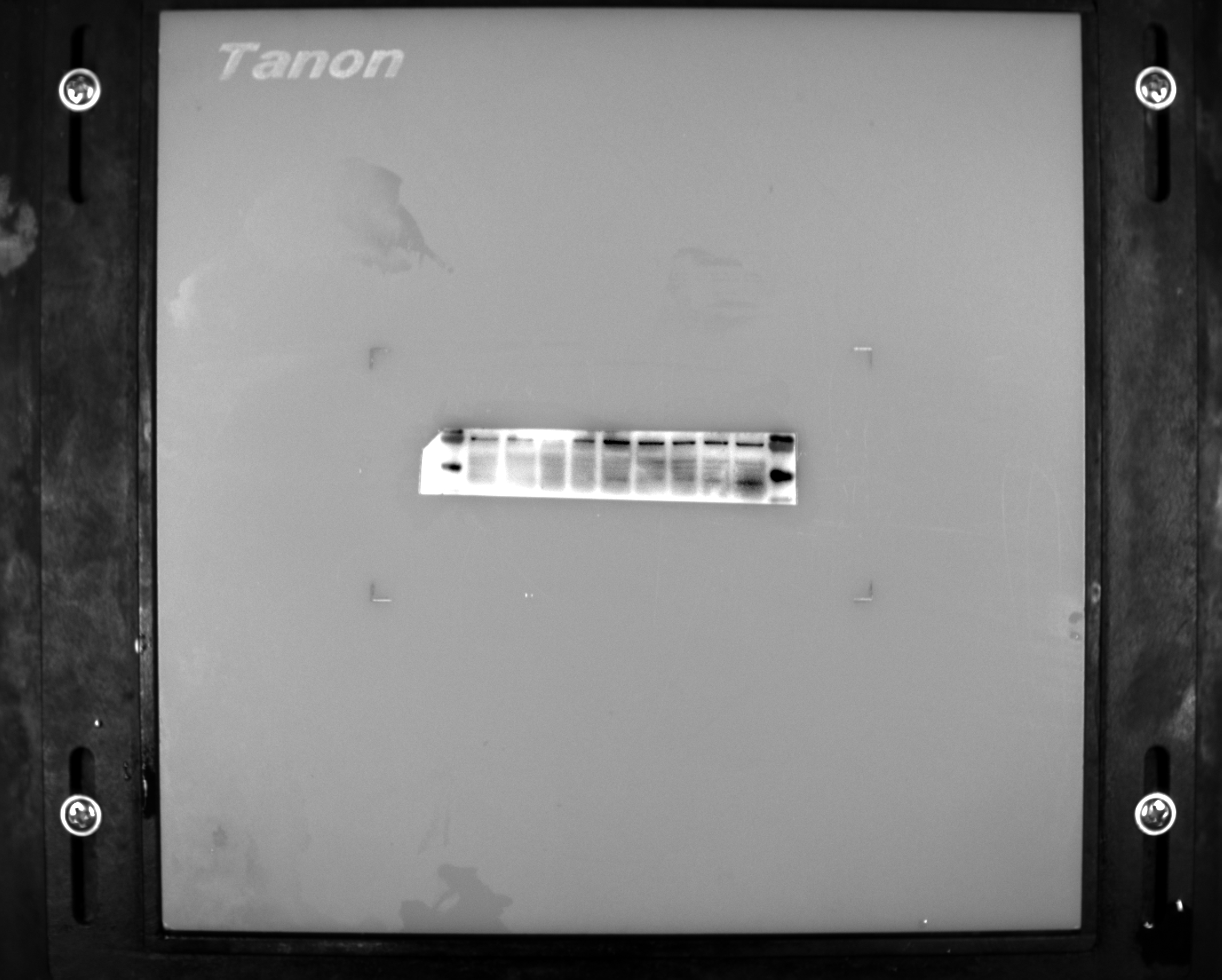

Supplement: Supplementary file 9 [file DataSheet_7.zip › Figure 8/PINK1.Tif]

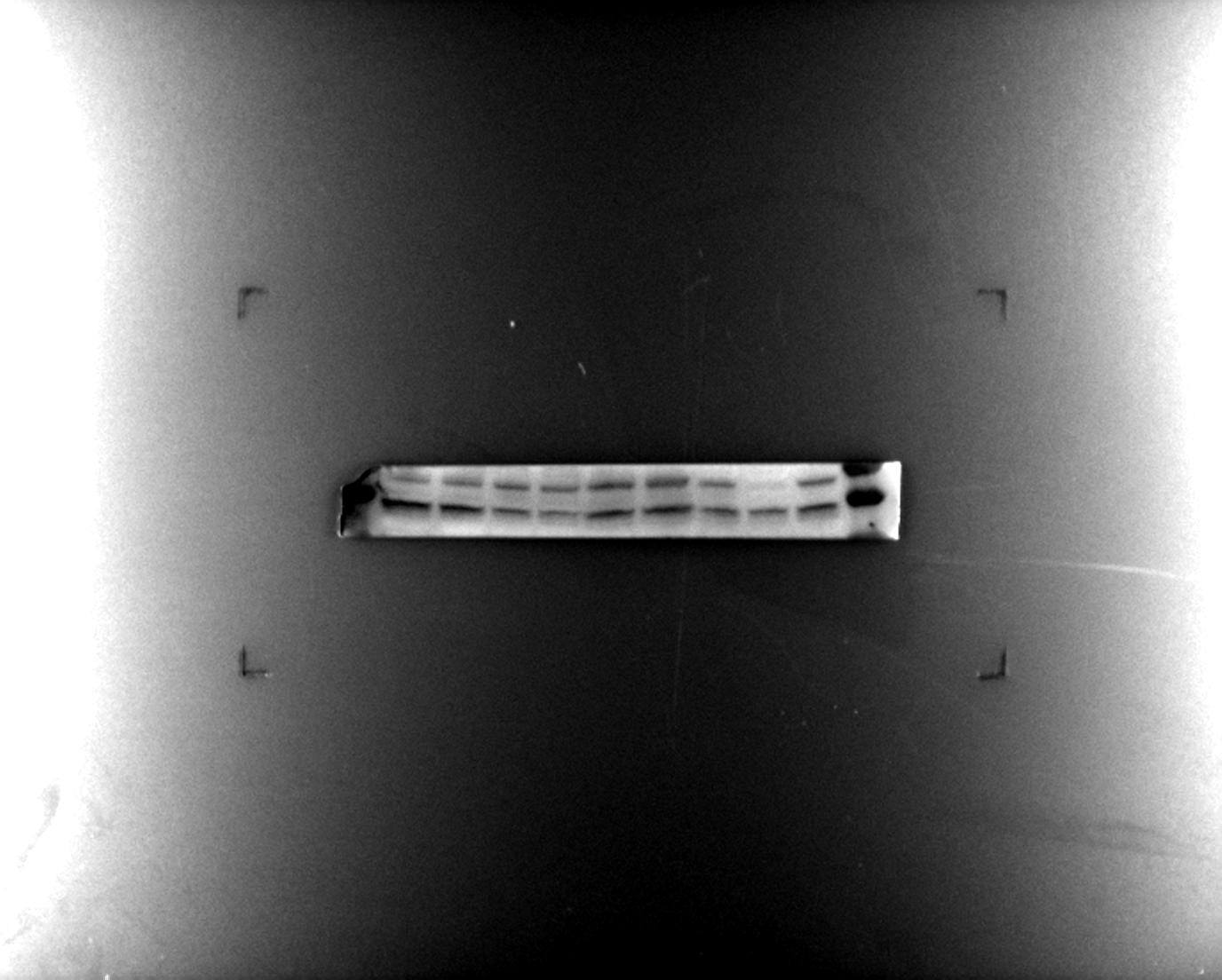

Supplement: Supplementary file 9 [file DataSheet_7.zip › Figure 8/Parkin.Tif]
