# Supplementary material for: NLRP3 Deficiency Protects Against Intermittent Hypoxia-Induced Neuroinflammation and Mitochondrial ROS by Promoting the PINK1-Parkin Pathway of Mitophagy in a Murine Model of Sleep Apnea
Source: Front Immunol. 2021 Feb 24;12:628168. doi: 10.3389/fimmu.2021.628168 (PMC7943742; doi:10.3389/fimmu.2021.628168)
Supplement: Supplementary file 10 [file Table_1.docx]

**Reviewer 1**

This manuscript does not meet the quality standards for publication in this journal. There are significant concerns related to the methods, over interpretation of results and conclusions made that are not supported by the data, and a general lack of coherent rationale with correct references. In addition, important details of the methodology are absent. Individual issues are elaborated below.

1) The rationale and link between OSA/CIH is weak. In the Intro, the first time microglia are mentioned is related to how sustained hypoxia affects them, nothing to do with CIH. Later in the manuscript, 2 papers by Karunakaren and Liu are cited to support the statement that microglia are the predominant contributor to the pathogenesis of CIH-induced neuroinflammation. But the Liu paper was done in vitro (BV-2 cells), and lacked evidence showing that the cells actually became hypoxic with 400s of low O2. Further, the data in that paper are not very convincing- the effects of CIH on p-p38 and IkBa degradation are modest at best. The Karunakaran paper does not even use IH. The authors erroneously leave the reader with the impression that there are data showing that microglia mediate the effects of CIH in the CNS, but that is not correct.

**Response:** Thanks for reviewer’s good comments. We are really sorry for our less rigorous behavior and many thanks for the kind reminder of reviewer. However, actually, CIH was the widely recognized and foremost pathophysiological process of OSA (Prabhakar NR et al. Hypoxia-inducible factors and obstructive sleep apnea. J Clin Invest. 2020). Moreover, it has been proven that microglia cells can be activated in a sustained hypoxic environment. So, the idea that similar results might occur in intermittent hypoxia has drawn the attention of researchers studying microglia in such environments. Besides, we have removed incorrect references and replaced appropriate ones for the whole manuscript.

2) There are many scientific statements that are made that are not referenced- several are not true. Examples include, but are not limited to: Intro 3rd para lines 9-10; Results 1st para lines 14-15; section 3.4 line 5; Discussion para 4 lines 1-2 and 10-11. I got tired of noting all of them so there are more.

**Response:** We are really sorry for our less rigorous behavior and many thanks for the kind reminder of reviewer. We have carefully reviewed our entire manuscript to ensure that each scientific statement was properly quoted. Meanwhile, we also simplified the content of the full text for the convenience of reading and understanding. If there are any deficiencies, we hope that the reviewer can give us a valuable opportunity to further modify them. Once again, thank you very much for your patient reading and valuable suggestions.

3) There are many conclusions and statements made that are not supported by the data. Results section 3.2 last line; section 3.3 lines 13-15; section 3.4 last line; section 3.5 para 2, lines 10 and 15 parkin-mediated mitophagy was not shown in the figures referenced (and when it was, it was in vitro using dubious transient transfection and a CIH protocol that is not physiologically relevant- see below); Discussion para 1, lines 4, 7 and 9- microglial activation was never shown, yet the conclusion is frequently stated. Mitophagy in mouse hippocampus was never shown- it was shown in BV-2 cells; Conclusion lines 1-2- it was not shown that NLRP3 ablation or inhibition orchestrated a reparative inflammatory response. The inflammatory response was tested.

**Response:** Thanks for reviewer’s good comments. We are sorry for the ambiguous explanation of the conclusions in the results sections. According to the reviewer’s suggestion, we have readjusted the conclusion both in the results and discussion sections to make it more accuracy and abundant.

4) There is concern with rigor and quality of several figures. It is stated that at least independent experiments were performed in duplicate, but that is most likely for the tissue culture experiments. How many n were used for the mouse experiments? What sex were they? How old were they? For tissue culture, it is difficult to get sufficient power s and statistical significance with a biological n=3. Hopefully the duplicate values were averaged per experiment and not counted individually.

**Response:** We are very sorry for our less rigorous behavior and thanks for the kind reminder of reviewer. The methodology of in vivo experiments of this study has been thoroughly described in Methods section: WT or NLRP3^−/−^ mice (male, 6–7 weeks old, 20–22 g) were randomly divided into 4 groups of six: the normal air (NA) plus WT mice group, the NA plus NLRP3-/- mice group, the CIH plus WT mice group, the CIH plus NLRP3-/- mice group.

5) The differences in ASC staining in Fig. 1B cannot be discerned. Fig 1C has a blot with 1 band from each of 4 treatments, and no quantification provided even though conclusions are made. The TUNEL and merged IHC images in Fig 1D are very poor and do not allow the reader to see the TUNEL staining even at 400X on the computer screen. The images provided should allow the reader to arrive at the same conclusions as the authors. The graph shows averaged data from the IHC that do not seem to be consistent with the images shown, and the indicate that 1/3 of the neurons are dead or dying in the CIH brain. That magnitude is very high and not consistent with other CIH studies in mouse, rat or human.

**Response:** Thank you very much for your comments. The image in Figure 1B was mainly to show the change of ASC expression in the whole hippocampus via using immunohistochemistry. Therefore, in those images, the stained area in yellow indicated positive expression of ASC protein. Furthermore, the darker the yellow was, the higher the expression of ASC protein was. As illustrated in Figure 1B, ASC was highly expressed in hippocampus of WT mice exposed to CIH. In contrast, the immunohistochemical staining showed that the level of ASC was faintly stained in the hippocampus of NLRP3^−/−^ mice upon CIH treatment. As a consequence, we thought the representative images of immunohistochemistry can also support our conclusions.

Because the images in the Figure1 were too much, we did not further add the quantitative histogram of Figure 1C on it. However, it was true that blots without quantification was less rigorous, so we provided the quantification of Figure 1C in the supplementary materials (Figure S1).

Following the suggestion of the reviewer, we uniformly increased the brightness of the red (TUNEL) and blue signal in raw images of TUNEL staining in all groups to make it more recognizable (Figure 1E). We hope that the current images can meet the requirements of reviewers. Besides, there were some other animal studies that detecting neuronal apoptosis by TUNEL staining in response to hypoxia came to the similar conclusions with us. For example, Halder and his colleagues found that after 14 days chronic mild hypoxia treatment, the approximately 30% of the neurons are dead in the brain (Halder SK et al. Chronic mild hypoxia accelerates recovery from preexisting EAE by enhancing vascular integrity and apoptosis of infiltrated monocytes. Proc Natl Acad Sci U S A. 2020). The relevant pictures are shown as following:


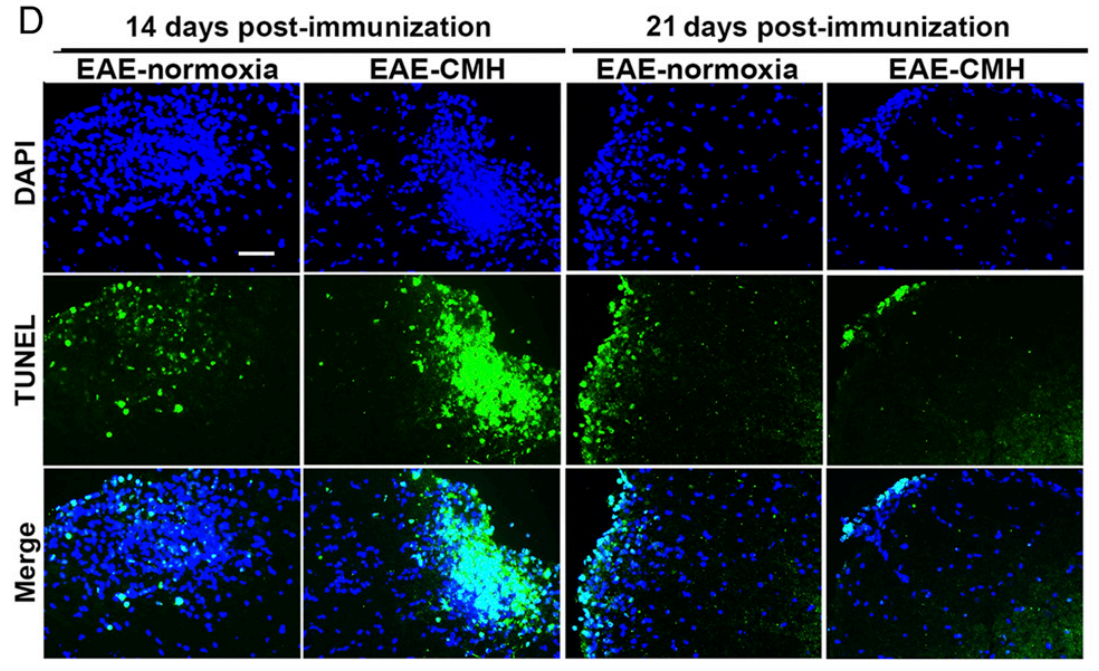


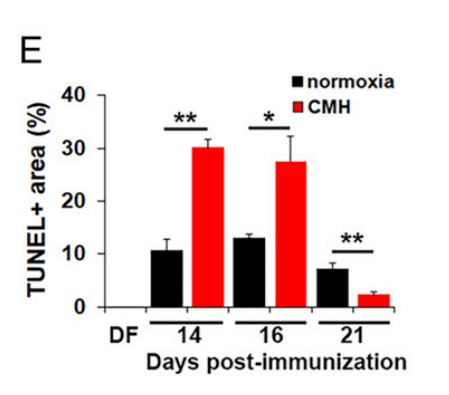


Moreover, similar results were obtained from another experiment about hypoxia-induced neuronal apoptosis (Martinello KA et al. Acute LPS sensitization and continuous infusion exacerbates hypoxic brain injury in a piglet model of neonatal encephalopathy. Sci Rep-Uk, 2019). We hope you'll find our explanation satisfactory. And, the typical images are shown below:


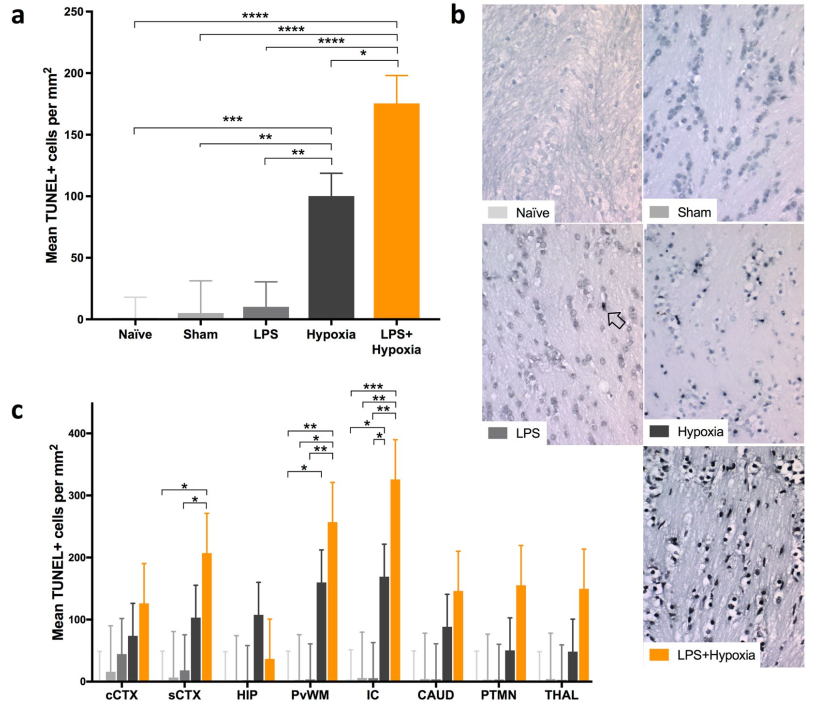


*Acute LPS sensitization and continuous infusion exacerbates hypoxic brain injury in a piglet model of neonatal encephalopathy*

6) In Fig.2A, the Iba1 staining does not look right. Why do the WT slices have hardly any microglia? That shouldn't be. The graph in Fig. 2B suggests that microglia proliferate and somehow cover 6X more area than the 10% they do in the WT and KO controls. That is not right- microglia don't proliferate in the CIH brain. Are the same regions of the CNS being compared in both NA and CIH groups?

**Response:** It is true as reviewer suggested that IBA1 is a marker expressed by microglia both in resting and activated status and should be stained in NA mice microglia. Actually, there were microglia cells that stained with IBA1 of WT mice in the raw images. In generally, the morphology of microglia in the resting condition most frequently showed a small and ovoid shape. While, after being activated, most of the microglial cells had rounded amoeboid morphologies with large and flat shape (Yang X et al. Resveratrol regulates microglia M1/M2 polarization via PGC-1α in conditions of neuroinflammatory injury. Brain, Behavior, and Immunity. 2017). These changes may cause the corresponding green fluorescence signal to be stronger (as shown in Figure 2A and 2B) and then it looks like there are more cells in the picture. The Figure 2B only revealed the fluorescence intensity of IBA1 in each group (microglia were activated after CIH treatment in vivo), but not to represent that microglia cells proliferating in the CIH brain. Moreover, the other studies related to microglia have reached similar results with us (Mao M et al. MicroRNA-195 prevents hippocampal microglial/macrophage polarization towards the M1 phenotype induced by chronic brain hypoperfusion through regulating CX3CL1/CX3CR1 signaling. J Neuroinflamm. 2020; Yang X et al. Resveratrol regulates microglia M1/M2 polarization via PGC-1α in conditions of neuroinflammatory injury. Brain, Behavior, and Immunity. 2017). However, in order to avoid the misunderstanding caused by our improper images, we swapped pictures of both NA group and CIH group for the other pictures of the same group taken at the same time. We hope that the current images can meet the requirements of reviewers. Based on the modifications mentioned above, the quantification of the fluorescent signal has been redone. At last, we compared the same regions of CNS in each group. The first two lines of images are showing the cortical regions of the brain, while the last two rows of images show hippocampus regions.

The relevant pictures for reference in the other articles are shown as follow:


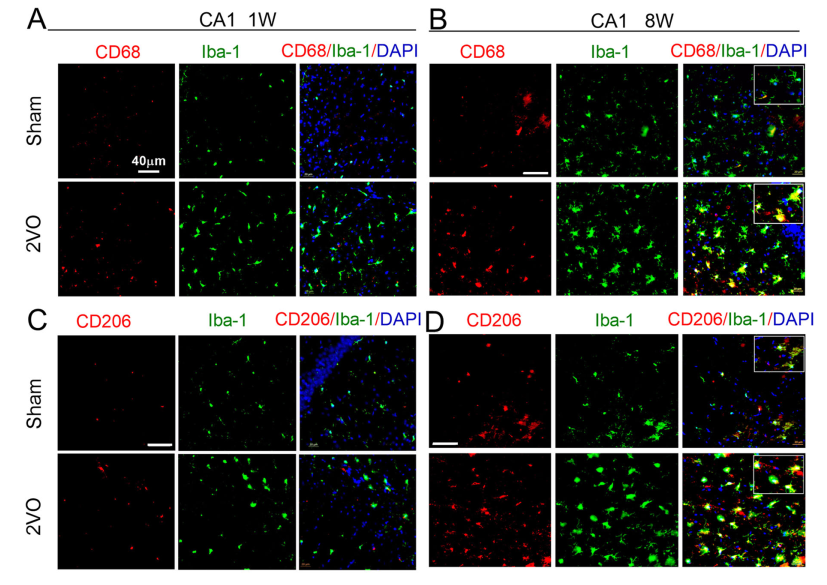


*MicroRNA-195 prevents hippocampal microglial/macrophage polarization towards the M1 phenotype induced by chronic brain hypoperfusion through regulating CX3CL1/CX3CR1 signaling*


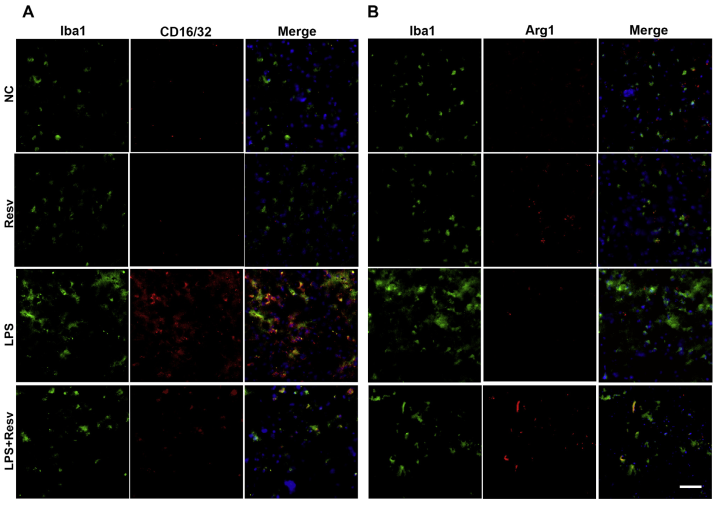


*Resveratrol regulates microglia M1/M2 polarization via PGC-1α in conditions of neuroinflammatory injury*

7) In Fig. 3B, the Iba1 staining doesn't look like it even stains microglia- that signal is not microglial.

**Response:** We are very sorry for our less rigorous behavior and thanks for the kind reminder of reviewer. As there was non-specific staining of IBA1 stained images in previous Figure 3B, so we redid the experiment with the same group of mice tissues (Figure 3B). We hope that the current images can meet the requirements of reviewers.

8) It is difficult to see that NLRP3 increases with CIH in the Western blot in Fig. 5A. The blots are not good quality and they have weird white areas, especially at right edge of ASC blot which could interfere with accurate quantification.

**Response:** Thanks for reviewer’s good comments. It is true as reviewer indicated the increases of NLRP3 protein expression in CIH group compared with NA group are not so much obvious. However, we carefully evaluated this set of blots (Figure 5A) and believed that the previously proposed trend was still valid. Because the molecular weight of the NLRP3 protein is not low (approximately 110KD), the blots of this protein is not easy to get very well. Moreover, the backgrounds of blots in this group were a little bit higher, due to the 3-5% BSA (Blocking buffer) was not reconfigured at that time. However, we thought that this group of blots was clear to identify the tendency we mentioned in the manuscript, so we did not redo this experiment of western blotting. Raw data for all western blots are available if there is any question. Therefore, we sincerely hope that we can get the understanding of editor and reviewers.

9) The utility of the BV-2 cell experiments is unclear. And studying their apoptosis really makes little sense, as they are immortalized (and do not reflect what happens in vivo). Even the authors show here that CIH induces microglial apoptosis in Fig. 5G, yet their in vivo data in Fig. 2B show that the microglia are more in number- definitely not apoptosing. How is this reconciled? Also, while the creation of stable cells lines using LV constructs is reasonable, transiently transfecting microglia is notoriously difficult and results in very poor efficiency. The authors do not show controls for the efficiency of their shRNA knockdown of parkin in Fig. 7. It is doubtful that transfection of shRNA with Lipofectamine 2000 is effective enough to measurably knockdown endogenous gene expression.

**Response:** It is reasonable as reviewer’s concern about the in vitro experiments using BV-2 cells can not completely simulate the pathophysiological changes of those cells in the in vivo experiments. And, this is a challenge for all experiments done using cell lines. Although, we still believe there is some value in using BV2 for in vitro experiments. First, we observed the same phenomenon in vivo and in vitro using both mouse and cell models, suggesting in vitro models can mimic in vivo biological processes to some extent. Second, BV-2 cell line was a widely used mouse microglia cell line, hence this also adds to the credibility of this in vitro experiment.
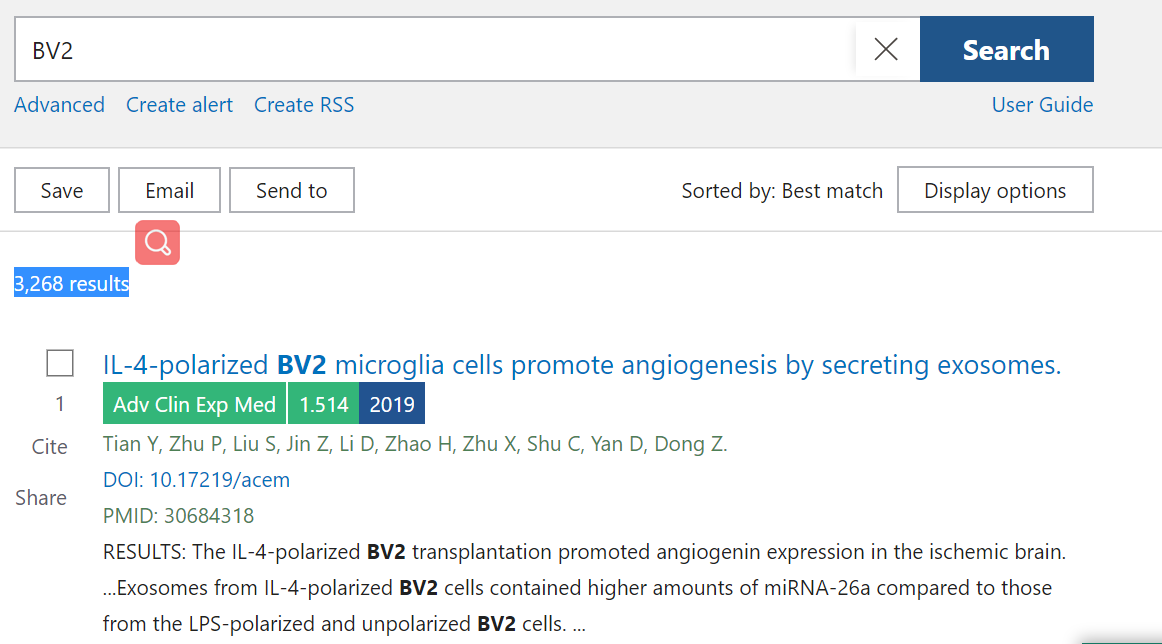
Last, previous study has used BV-2 cells to establish an in vitro IH model (Shi Y et al. DNA binding protein HMGB1 secreted by activated microglia promotes the apoptosis of hippocampal neurons in diabetes complicated with OSA. Brain Behav Immun. 2018), indicating this in vitro model is acceptable to a certain extent. However, the in vitro BV-2 model certainly has some limitations, we will also further optimize this model in future studies. We hope we can get the understanding of reviewers and editors.

Previously, we suggested that mitochondrial (mt) damage was a potential cause of NLRP3 inflammasome activation (Wu X et al. NLRP3 inﬂammasome mediates chronic intermittent hypoxia‐induced renal injury implication of the microRNA‐155/FOXO3a signaling pathway. Journal of Cellular Physiology. 2018). Shimada et al. also demonstrated that NLRP3 inflammation can be activated through mt damage-induced apoptotic cascade (Shimada K et al. Oxidized Mitochondrial DNA Activates the NLRP3 Inflammasome during Apoptosis. Immunity. 2012) . Moreover, it has also been proven that the combined roles for caspase-8 and caspase-1/NLRP3 causing IL-1β maturation (Gurung P et al. NLRP3 inflammasome plays a redundant role with caspase 8 to promote IL-1β–mediated osteomyelitis. Proceedings of the National Academy of Sciences. 2016), indicating the crosstalk between apoptosis and pyroptosis (Zheng et al. The regulation of the ZBP1‐NLRP3 inflammasome and its implications in pyroptosis, apoptosis, and necroptosis (PANoptosis). Immunological Reviews. 2020). According to these results, we detected the apoptosis in CIH-induced BV-2 cells.

As for Figure 2B, it only revealed that the fluorescence intensity of IBA1 in each group (microglia were activated after CIH treatment in vivo), but not to represent the proliferating or apoptosis of microglia cells in the CIH brain.

It is true as reviewer’s concern about transiently transfecting microglia is notoriously difficult. But it's not completely ineffective (Wang Z et al. Critical role of NLRP3-caspase-1 pathway in age-dependent isoflurane-induced microglial inflammatory response and cognitive impairment. J Neuroinflamm. 2018; Zhang Q et al. Activation of the alpha7 nicotinic receptor promotes lipopolysaccharide-induced conversion of M1 microglia to M2. Am J Transl Res 2017). Meanwhile, we showed controls for the efficiency of our shRNA knockdown of parkin in Figure 8A. As its showing, though the Parkin protein expression was not been completely knocked out, there was a significant decrease in protein expression compared to the control group. Therefore, we think that using shRNA strategy is feasible under these conditions.

10) The amount of necessary detail included in the Methods section is absolutely inadequate. The number of days that the mice were exposed to CIH was not included. The reference provided in that paragraph is a number (11) while the references for this journal are alphabetical by author. It is a significant omission to not indicate the fundamental parameters of the CIH exposure. Guessing that the previous study they refer to was Wu 2018, I went to that paper and it referenced Wu 2016. I went to that paper and it cited another paper Fu et al, but at least did mention that it was 7d/week for 5 weeks. Fu also used 5 weeks. It should not be a mystery what the experimental parameters are for an experiment. And the reader should not have to be a detective and be referred through several layers of papers in order to find a critical aspect of the study that is necessary to interpret the data. The same thing happened with the BV2 cell CIH experiments. The authors referenced the Wu paper, which references another paper (Almendros). Again, a reader shouldn't have to go to 3 different places to figure out what was done here. When I finally found what the treatment parameters were they were 1 cycle per hour- normoxia was 30', Hx 30'. I still don't know how many hours of exposure the cells received this. The in vivo AHI was 30, and in tissue culture it is 1. The in vitro system does not accurately model anything in vivo, and certainly not sleep apnea. It is acknowledged that soluble O2 levels don't change as rapidly as inspiring gas in vivo. But this just further underscores the questionable utility of the tissue culture experiments and why most people who properly study sleep apnea don't do it in vitro.

**Response:** We are really sorry for our less rigorous behavior and the methodology has been thoroughly described in this manuscript. We are appreciated the kind reminder of reviewers and editors, such mistakes will also be eradicated by us in the future. And, the hours of exposure the cells received were also added correspondingly.

It is true as reviewer mentioned there were some differences in the CIH model between in vivo and in vitro. However, the model we were currently using was based on previous studies and experiences (Fu C et al. Chronic intermittent hypoxia leads to insulin resistance and impaired glucose tolerance through dysregulation of adipokines in non-obese rats. Sleep and Breathing. 2015; Thomas A et al. Chronic Intermittent Hypoxia Impairs Insulin Sensitivity but Improves Whole-Body Glucose Tolerance by Activating Skeletal Muscle AMPK. Diabetes. 2017; Song Jet al. Heterozygous SOD2 deletion deteriorated chronic intermittent hypoxia-induced lung inflammation and vascular remodeling through mtROS-NLRP3 signaling pathway. Acta pharmacologica Sinica. 2020). Although there may be some controversy, we still believed that the current experimental scheme was relatively credible. And, we will also explore more reliable models and experimental methods in our future study. We hope we can get the understanding of editors and reviewers.

Reviewer 2

**1.** As stated below my recommendation to authors is to reduce the number of figures, maybe preparing supplementary material with the figures that are not key for the manuscript such as those isolating and characterizing the knockouts. Measuring the IL1b particularly in the mice model brain tissue will give some strength to this work as well incorporating some human derived cells.

**Response:** Thanks for reviewer’s good comments. According to the reviewer’s suggestion, we put Figure 9 into the supplementary materials as Figure S2. However, we still thought the characterizing of the gene knockouts was important after our careful discussion. Therefore, we did not simplify the images any further. We hope we can get the understanding of the reviewer. If there are any problems, we will be willing to correct them further.

It is true as reviewer suggested that measuring the IL-1β in the mice model brain tissue will give strength to this work. Hence, we used qRT-PCR to detect the IL-1β mRNA levels in the frozen hippocampus tissues of mice and the data was shown in Figure 1D. And, we also added the contents of clinical studies in OSA patients with their associated cognitive impairment in the discussion part, which we hope we can get the understanding of the reviewer.

**2.** Limitations are that no human cells are used, all the work is done with mouse, mouse-derived tissues or a murine cell line. Another limitation is that the main focus of the work rests in the mitophagy analysis, it will make stronger the work to measuring IL1beta for example. And lastly the manuscript is too long, the number of figures needs to be reduced. Despite all limitations, this work is very good and deserves to be published.

**Response:** Thanks for reviewer’s good comments. It is true as reviewer suggested that including some explicit works done with humans will give strength to this study. Nevertheless, due to the resource limitations, it is not possible to collect human derived materials at this time. Thus, we added the contents of clinical studies in OSA patients with their associated cognitive impairment in the discussion part, which we hope we can get the understanding of the reviewer. Besides, to give more strength to this work in detecting NLRP3-mediated inflammation, we used qRT-PCR to detect the IL-1β mRNA levels in the frozen hippocampus tissues of mice and the data was shown in Figure 1D. At last, we have carefully simplified the content of the full text according to the reviewer’s suggestion. And, if there are any deficiencies, we hope that the reviewer can give us a valuable opportunity to further modify them. Once again, thank you very much for your patient reading and valuable suggestions.

**3.** The methods, results and data interpretation are adequate, missing are labeling of the images in Figure 4 E and F either in the figures or in the legends for them. The discussion needs more work and If experiments including human derived materials are not possible at this time, then explicit work done with humans should be discussed.

**Response:** Thanks for reviewer’s good comments. According to reviewer’s suggestion, we enhanced the labeling of the images in Figure 4E and 4F either in the figures. The red fluorescence represents TOM20, while the green fluorescence represents Parkin. For all images, the bars (white lines) equal to 50 μm. Moreover, in Figure 4F, images in the bottom raw are the higher magnification of the images in the first row, which we hope to demonstrate the morphology of mitochondria more clearly. As we mentioned above, it is true as reviewer suggested that including some explicit works done with humans will give strength to this study. Nevertheless, due to the resource limitations, it is not possible to collect human derived materials at this time. Thus, we added the contents of clinical studies in OSA patients with their associated cognitive impairment in the discussion part, which we hope we can get the understanding of the reviewer.

Reviewer 3

1. For the first time in literature, in a model of sleep apnea, it has been shown that the Parkin system of Mitophagy is releated to the deficiency of NLRP3. Within sophisticated methodologies the authors could respond to all the hypotheses raised in the introduction section.
However, one of the limitations is regarding the conclusion in the end of the Results paragraphs. Some of them do not summarize the main findings.

**Response:** Thanks for reviewer’s good comments. We are sorry for the ambiguous explanation of the conclusions in the results sections. According to the reviewer’s suggestion, we have readjusted the conclusion in the results sections to make it more accuracy and abundant.

2. The conclusions in some results sections are too simplified to resume what that result means. For example, in the 3.3 section, line 13 the authors mentioned the “above results” to mention the 3.2 results with mtROS production. I think this explanation fits better in the section 3.2, because the 3.3 section is only about mitophagy involvement.

**Response:** Thanks for reviewer’s good comments. We are sorry for the ambiguous explanation of the conclusions in the results sections. According to the reviewer’s suggestion, we have readjusted the conclusion in the results sections to make it more accuracy and abundant.

3. In Figure 1B, I suggest a square draw to emphasize what are the differences between the images.

**Response:** It is true as reviewer suggested that to emphasize what are the differences between the images is quite important. Hence, we marked the specific brain regions (CA1 and CA4) in Figure 1B. However, as the image of this part was mainly to show the change of ASC expression in the whole hippocampus, we did not further used squares to point out the specific area of these images. We hope we can get the understanding of reviewers and editors.

4. To explain the Figure 1A, the authors wrote that the comparison between the CIH-group and NA group in the freezing context were different with a p<0.01 but in the description of the figure the authors show a p<0,05. It is confused.

**Response:** We are very sorry for our less rigorous behavior and thanks for the kind reminder of reviewer. The expressions in figure legends of Figure 1A was incorrect, as the comparison between the CIH-group and NA group in the freezing context should be different with a p<0.01. The corresponding contents have been corrected.

5. In figure 3B the authors showed the induction of mitophagy using LC3 marker and Microglia activation usiend IBA1 marker. In the figure 2A the authors concluded that the microglia activation was low in Nlrp3 knockout compared to WT in the CIH-group. Here, in figure 3B, it seems that IBA1 in Nlrp3 knockout CIH-group is higher than WT, contradicting the figure 2A conclusion.

**Response:** Thanks for reviewer’s good comments. And, we are very sorry for our less rigorous behavior. As mentioned above, the morphology of microglia in the resting condition most frequently showed a small and ovoid shape. While, after being activated, most of the microglial cells had rounded amoeboid morphologies with large and flat shape. These changes may cause the corresponding green fluorescence signal to be stronger (as shown in Figure 2A and 2B) and then it looks like there are more cells in the picture. In order to avoid the misunderstanding caused by our improper images, we swapped pictures of Figure 2A for the other pictures of the same group taken at the same time. Meanwhile, as there was non-specific staining of IBA1 stained images in previous Figure 3B, we redid the experiment with the same group of mice tissues, as shown in Figure 3B. We hope that the current images can meet the requirements of reviewers.

6. In the introduction, the first paragraph, line 8, I miss a reference after “Proinflammatory mediators”
**Response:** We are very sorry for our less rigorous behavior and thanks for the kind reminder of reviewer, the appropriate reference has been cited correspondingly.

7. In the introduction, second paragraph, line 7, after “IL-1β” I miss a reference. In this same paragraph the authors explain the formation of the NLRP3 Inflammasome just when NLRP3 and Asc interacts leading to caspase-1 cleavage, but, the NLRP3 inflammasome is formed by NLPR3, Asc and Inactivated Caspase-1, so I suggest this alteration to avoid a misconception.

**Response:** We are sorry for the ambiguous explanation of the formation of the NLRP3 Inflammasome. It is true as reviewer suggested that the NLRP3 inflammasome is formed by NLPR3, Asc and Inactivated Caspase-1, thus we have corrected the corresponding contents of the article. Besides, we are very sorry for our negligence that we missed a reference; the appropriate reference has been cited according to the reviewer’s suggestion.

8 In the last paragraph of the introduction, line 4, the authors used the expression “The present study will reveal…” I suggest the sentence in the present tense, not in future tense.

**Response:** Thanks for reviewer’s good comments. According to reviewer’s suggestion, we changed the corresponding sentence into the present tense.

9. Also in the introduction section I miss a brief of phrases about the role of NLRP3 in apoptosis, because it is well established the role of this protein in pyroptosis, but it is not common to discuss it in other cell death mechanisms.

**Response:** Following the suggestion of the reviewer, we added the contents of the role in NLRP3 in apoptosis in the introduction section in the Paragraph 3, lines 94-99. As the reviewer mentioned, adding this section helped to understand the whole experiments.

10. Line 7 of the 4th paragraph of the Discussion section - the authors wrote “pyropoptosis” but the correct writing is “pyroptosis”.

**Response:** We are sorry for the spelling mistake as we had made and thanks for the kind reminder of reviewer. We have changed the “pyropoptosis” into “pyroptosis”.

11. Also in the discussion first paragraph, line 6, the authors wrote “As respected” but to me the intention is to write “As expected”.

**Response:** We are very sorry for our less rigorous behavior and thanks for the kind reminder of reviewer. The corresponding contents of the article have been corrected.
